# Bull Sperm SWATH-MS-Based Proteomics Reveals Link between High Fertility and Energy Production, Motility Structures, and Sperm–Oocyte Interaction

**DOI:** 10.1021/acs.jproteome.3c00461

**Published:** 2023-10-02

**Authors:** Sabrina Gacem, María Castello-Ruiz, Carlos O. Hidalgo, Carolina Tamargo, Pilar Santolaria, Carles Soler, Jesús L. Yániz, Miguel A. Silvestre

**Affiliations:** † Departamento de Biología Celular, Biología Funcional y Antropología Física, Universitat de València, 46100 Valencia, Spain; ‡ Departamento de Medicina y Cirugía Animal, Universitat Autònoma de Barcelona, 08193 Barcelona, Spain; § Unidad Mixta de Investigación Cerebrovascular, Instituto de Investigación Sanitaria La Fe, Hospital Universitario y Politécnico La Fe, 46026 Valencia, Spain; ∥ Animal Selection and Reproduction Area, Regional Agrifood Research and Development Service (SERIDA), 33394 Deva, Gijón, Spain; ⊥ BIOFITER Research Group, Institute of Environmental Sciences (IUCA), University of Zaragoza, 22071 Huesca, Spain

**Keywords:** flow cytometry, computer-assisted sperm analysis, sperm, bull, fertility, proteome

## Abstract

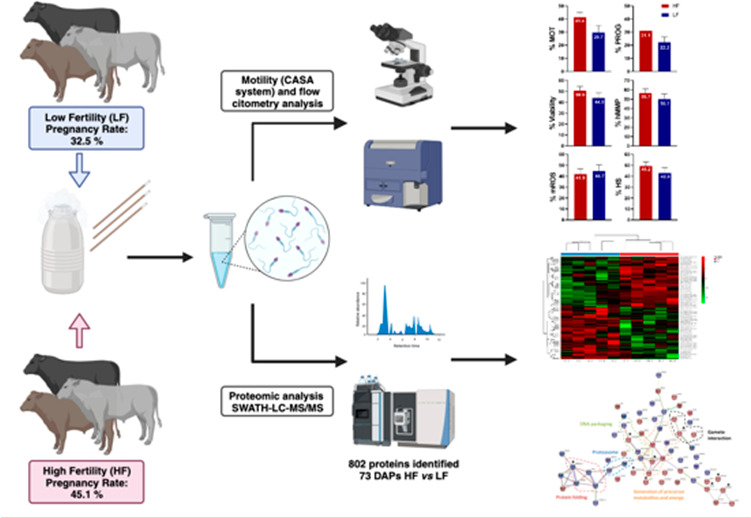

The prediction of male or semen fertility potential remains a persistent challenge that has yet to be fully resolved. This work analyzed several *in vitro* parameters and proteome of spermatozoa in bulls cataloged as high- (HF; *n* = 5) and low-field (LF; *n* = 5) fertility after more than a thousand artificial inseminations. Sperm motility was evaluated by computer-assisted sperm analysis. Sperm viability, mitochondrial membrane potential (MMP) and reactive oxygen species (mROS) of spermatozoa were assessed by flow cytometry. Proteome was evaluated by the SWATH-MS procedure. Spermatozoa of HF bulls showed significantly higher total motility than the LF group (41.4% vs 29.7%). Rates of healthy sperm (live, high MMP, and low mROS) for HF and LF bull groups were 49% and 43%, respectively (*p* > 0.05). Spermatozoa of HF bulls showed a higher presence of differentially abundant proteins (DAPs) related to both energy production (COX7C), mainly the OXPHOS pathway, and the development of structures linked with the motility process (TPPP2, SSMEM1, and SPAG16). Furthermore, we observed that equatorin (EQTN), together with other DAPs related to the interaction with the oocyte, was overrepresented in HF bull spermatozoa. The biological processes related to protein processing, catabolism, and protein folding were found to be overrepresented in LF bull sperm in which the HSP90AA1 chaperone was identified as the most DAP. Data are available via ProteomeXchange with identifier PXD042286.

## 1 Introduction

Fertility has a considerable impact on both dairy and beef cattle production.^1^ The most widely used assisted reproductive technology in the dairy cattle industry is artificial insemination (AI). However, cow pregnancy after AI hardly exceeds 50%.^2,3^ The early detection of infertile and subfertile bulls has the potential to mitigate substantial economic losses arising from reduced fertility efficiency in the dairy industry.^4,5^ Conducting a breeding soundness examination, which includes *in vitro* sperm quality assessment, is imperative to identify infertile or subfertile males. Despite these controls, a subset of AI bulls exhibiting acceptable or normal sperm quality values still produce *in vivo* fertility deviations.^6^ Thus, distinguishing subfertile males could require more complex male fertility prediction models that include multiple *in vitro* sperm quality parameters.^4,7^ In humans, fertility problems affect approximately 15–20% of couples, and a male factor was involved in half of the cases according to the World Health Organization.^8,9^ Despite the economic significance of this species, the bovine model presents a valuable opportunity to investigate sperm-related factors associated with fertility because of the field fertility data available on bulls that have undergone several hundred AIs.

Enhanced protocols for the *in vitro* assessment of sperm quality, coupled with a deeper comprehension of the connection between sperm parameters and field fertility, are imperative to improve the early identification of subfertile males. In this way, computer-aided sperm analysis (CASA) systems and flow cytometry (FC) represent effective tools for evaluating a wide range of *in vitro* sperm quality parameters in a large number of spermatozoa. Although several studies have found a correlation between field fertility and *in vitro* sperm parameters, such as motility, viability, acrosome status, and DNA fragmentation,^6,10−12^ this relationship is not evident and remains poorly understood. Indeed, establishing a consistent correlation between fertility and a single parameter is challenging,^6,13^ and therefore, it is crucial to consider a combination of multiple parameters to elucidate the variability in fertility among semen samples in several livestock species.^7,14,15^ Despite recent advances, current methods of sperm assessment are insufficient to properly explain and predict the fertility potential of individual males. At best, different studies indicated that several *in vitro* sperm parameters, measured by CASA and FC, explained around 40–50% of the variation in bull fertility.^7,11,14^ When investigating the correlation between *in vitro* sperm parameters and fertility, it is crucial to take into account the number of AIs per bull, as this represents a major influencing factor in fertility prediction.^14,16^ Another important factor to consider is that fertility among groups of bulls with high and low fertility should be sufficiently divergent.^14^

In the past decade, the analysis of sperm proteome has progressively emerged as a new strategy to find biomarkers for male fertility potential prediction.^17−19^ Peddinti et al. in 2008 published the first comprehensive study on proteomic sperm analysis.^20^ Since then, some studies comparing bull spermatozoa from different fertility indexes using different proteomic techniques have been published. This led to the identification of several proteins that were either up- or downregulated with respect to fertility. New advances in technology have improved proteomics techniques in the last years, and mass spectrometry (MS) has become the method of choice due to its unrivaled ability to analyze complex protein mixtures like spermatozoa. Data-dependent acquisition (DDA) is a widely used mode of data collection in tandem with mass spectrometry (MS/MS) sperm proteins from Holstein Friesian^21−24^ or crossbreed bulls.^25−27^ In 2013, Gillet et al.^28^ proposed a new proteomic strategy using a data-independent acquisition (DIA) method called SWATH-MS (sequential window acquisition of all theoretical mass spectra). The SWATH-MS method is particularly innovative among the DIA methods and presents several advantages (reproducibility and consistency, data acquisition, and quantifiable proteins) compared with DDA-based methods and targeted proteomics.^29,30^ Sperm proteomic analysis using SWATH-MS provides highly valuable information on cellular components or sperm function, which, together with the data obtained from the *in vitro* parameters obtained by FC or CASA, offers a more complete picture of biological events. However, to the best of our knowledge, there has not been any research combining SWATH proteomic analysis and *in vitro* sperm parameters published. Therefore, this work analyzed several *in vitro* parameters evaluated by CASA and FC and proteome evaluated by SWATH-MS of bull spermatozoa cataloged as high- and low-field fertility after more than a thousand AIs.

## 2 Materials and Methods

### 2.1 Reagents and Media

The chemicals used for the extension of sperm samples and the fluorescent stains employed for the *in vitro* sperm assessment were procured from Sigma-Aldrich (Merck Life Science S.L.U., Madrid, Spain). Tyrode’s medium (TL) containing lactate and pyruvate was used (11.4 mM NaCl, 3.2 mM KCl, 0.4 mM NaH_2_PO_4_·H_2_O, 2 mM Ca_2_Cl·2H_2_O, 0.5 mM MgCl_2_·6H_2_O, 0.03 g/L of penicillin, 2 mL/L of phenol red, 10 mM sodium lactate, 1 mM sodium pyruvate acid, and 25.0 mM NaHCO_3_). Dulbecco′s phosphate-buffered saline (PBS; D8662) was supplemented with 6 g/L of BSA (A7906) to assess sperm motility (PBS-BSA).

### 2.2 Bull Fertility Data and Semen Collection

Frozen semen doses were collected from 10 Holstein Friesian bulls (*Bos taurus*) housed at the Cenero Artificial Insemination Centre (Gijón, Spain). These 10 males were selected from 559 bulls for both their field fertility (pregnancy rate) and high number of AIs (with an average of 2716 ranging from 1264 to 9770 AIs). The bulls were classified into two groups according to their field fertility: five bulls were classified as high-fertility (HF) and the other five were classified as low-fertility (LF). Average pregnancy rate (PR; %) was 45.13 ± 0.47 and 32.55 ± 0.63 for HF and LF, respectively. Field fertility was determined through a retrospective study using data from the first and second AI in cows and heifers. Pregnancy rate was assessed by ultrasonography or rectal palpation 35–45 days after AI. Only ejaculates exhibiting individual sperm motility higher than 70%, as determined by CASA, were selected for cryopreservation. Semen samples were then diluted with the Bioxcell extender (IMV Technologies, L’Aigle, France) to a final concentration of 23 × 10^6^ sperm at room temperature and were frozen in 0.25 mL straws (IMV Technologies, L’Aigle, France) using a programmable freezer following standard procedures previously established.^31^ After the freezing procedure, quality control of the semen doses was performed. Three randomly selected doses of semen from each freezing batch were evaluated by using a CASA system. The cutoff value for progressive motility was greater than 40%.

### 2.3 Preparation of Sperm Samples

All experimental procedures were carried out on individual samples of each bull from the two categories (HF and LF) obtained from three frozen straws. The straws from the same ejaculate were thawed in a water bath at 37 °C for 1 min, and their contents were pooled and centrifuged at 956*g* for 15 min (Minispin, Eppendorf) at room temperature on a Percoll monolayer gradient (45% in TL [v/v], Percoll; P4937) to isolate the spermatozoa from other putative cells and debris.^32^ The sperm pellets were extended in TL and centrifuged again (956*g*, 5 min at rt). For proteomic analysis, the sperm samples were washed five times in PBS (centrifugation at 956*g* for 5 min at rt), and the resulting pellet was stored at −20 °C until further analysis.

### 2.4 Sperm Motility Assessment

After thawing and before any centrifugation, 2 μL of semen sample was diluted in 25 μL of PBS-BSA. The diluted semen sample was placed in a prewarmed ISAS D4C10 chamber (Proiser R+D S.L., Paterna, Spain), and sperm motility was assessed with a computer-assisted sperm analyzer (CASA-Mot; ISAS, version 1.2; PROISER, Paterna, Spain) with a Proiser HS640 M digital camera with 50 frames/s. Spermatozoa were classified as progressive if VCL > 20 μm/s and STR > 80%. The motility variables measured included the total sperm motility (MOT) representing the population of sperm with VCL > 10 μm/s ( %), progressive motility sperm with VCL > 10 μm/s and STR > 80% (PROG, %), curvilinear velocity (VCL, μm s^–1^), straight line velocity (VSL, μm s^–1^), average path velocity (VAP, μm s^–1^), straightness (STR; VSL/VAP × 100), sperm linearity (LIN; VSL/VCL × 100), wobble (WOB), and amplitude of lateral sperm head displacement (ALH, μm).

### 2.5 Viability, Mitochondrial Membrane Potential, and Reactive Oxygen Species of Spermatozoa Assessment

The FC assessments were performed by the core facility of cell culture and flow cytometry in the Central Service for Experimental Research (SCSIE) at the University of Valencia. Multiparametric FC analyses were conducted using a BD LSRFortessa flow cytometer equipped with five lasers emitting UV wavelengths at 355 nm, blue at 488 nm, yellow-green at 561 nm, violet at 405 nm, and red at 640 nm. The system was controlled by using FACSDiva 8 software. A minimum of 7500 cells per replicate were recorded, and flow rate was maintained at 500–1500 cells/s.

**Figure 1:**
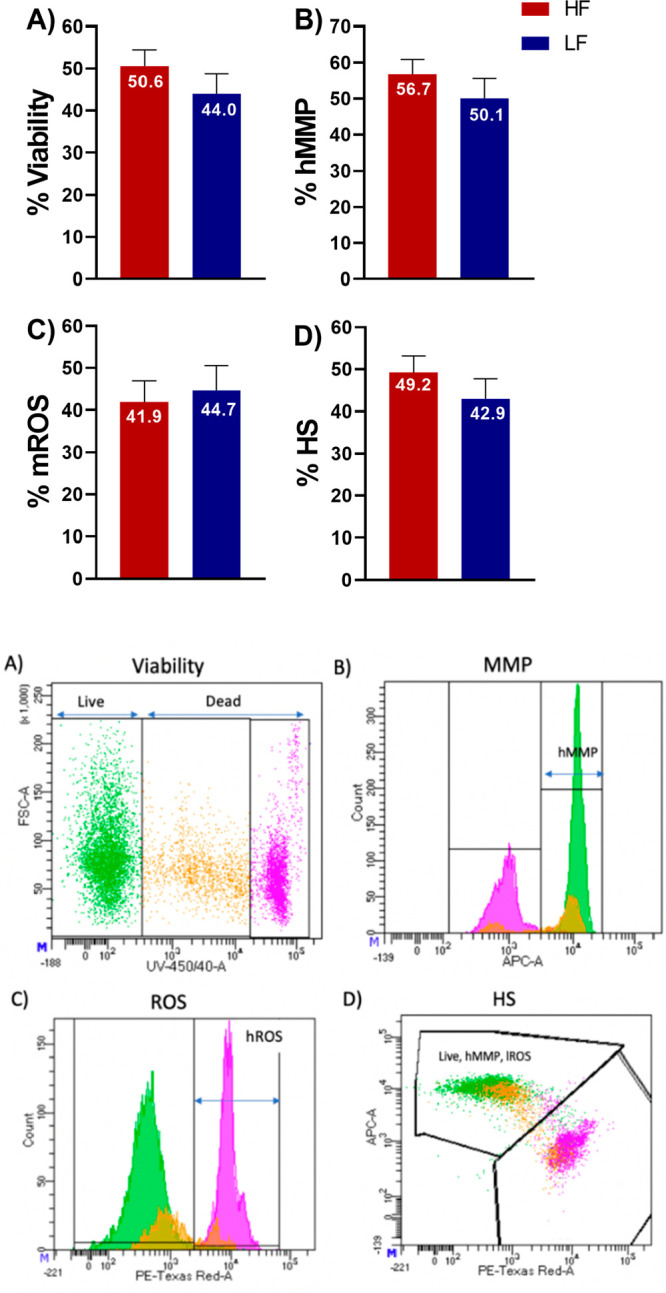
Flow cytometry analysis of the rates of viability, high mitochondrial membrane potential (hMMP), and mitochondrial reactive oxygen species (mROS) of spermatozoa in high-fertility (HF) and low-fertility (LF) bulls. (A) Viability: DAPI-negative cells were considered live sperm (green). DAPI-positive cells were considered dead and dying sperm (pink and orange). (B) MMP: Mitotracker Deep Red positive cells were considered as sperm with high mitochondrial membrane potential (hMMP). (C) ROS: Mitosox Red positive cells were considered as sperm with high amounts of superoxide (mROS). (D) HS: healthy sperm; live sperm with hMMP and low mROS were considered as healthy sperm.

After Percoll centrifugation, viability, mitochondrial membrane potential (MMP), and mitochondrial reactive oxygen species (mROS) of spermatozoa were assessed by FC. To evaluate sperm viability (plasma membrane integrity), stain 4′,6′-diamidino-2-phenylindole (DAPI; D9542; 1 μg/mL) was used. DAPI was detected with peak excitation at 355 nm and emission at 450/40 nm BP. DAPI positive and negative cells were considered as dead and living cells, respectively (Figure 1a).^33,34^ Mitochondrial membrane potential was assessed using Mitotracker Deep Red (MTDR; M22426; Invitrogen, 100 nM). MTDR was detected as a peak excitation at 640 nm and emission at 670/14 nm. MTDR positive and negative cells were considered as sperm with high and low MMP, respectively (Figure 1a).^35^ Mitosox Red (MSOX) was used to study amounts of mROS (superoxide anion radical) produced by mitochondria (M36008; Invitrogen, 1 μM). MSOX was detected at peak excitation at 561 nm and emission at 610/20 nm. MSOX positive and negative cells were considered as sperm with high and low amounts of superoxide, respectively (Figure 1a).^36^ Semen samples were incubated with a mix of fluorescents DAPI/MTDR/MSOX for 15 min at 37 °C in the dark. After incubation, samples were washed and analyzed by FC.

### 2.6 Sperm Proteomics

The proteomics analyses were carried out in the Proteomics Unit at the University of Valencia (member of the PRB2-ISCIII ProteoRed Proteomics Platform).

#### 2.6.1 Protein Extraction

A volume of 50 μL of 1.5× Laemmli Buffer (Bio-Rad, Hercules, CA, United States) was added to the sperm sample pellets to extract protein. The combination was mixed for 30 min, sonicated for 5 min, and centrifuged at 13 000 rpm for 5 min. The supernatant with total protein extract was quantified using Macherey-Nagel quantification reagent (Macherey-Nagel, Düren, Germany) following the manufacturer’s instructions.

#### 2.6.2 In-Gel Digestion Processing

To build the spectral library, aliquots with an equal amount (2.5 μg/sample) of all samples from the HF and LF groups were mixed and charged into one-dimensional sodium dodecyl sulfate–polyacrylamide gel electrophoresis (1D SDS-PAGE) (total amount, 12.5 μg/well). Each individual lane was cut into two pieces, and the contained proteins were reduced for 30 min at 60 °C in dithiothreitol and digested overnight at 37 °C with 250 ng of trypsin (Promega, Madison, WI, United States) following the protocol used by Shevchenko et al.^37^ Trifluoroacetic acid (TFA, 1%) was added to stop the protein digestion, and after a double extraction with acetonitrile (ACN) and drying in a speed vacuum, the peptide mixture was resuspended with 15 μL of 2% ACN and 0.1% TFA. For the SWATH analysis of individual samples, a protein extract (7.5 μg) of each sample (*n* = 5 for HF and LF group) was loaded in a 1D SDS-PAGE. After cutting each individual lane, the proteins were digested, and the peptides were extracted following the same protocol described above.

#### 2.6.3 Liquid Chromatography and Tandem Mass Spectrometry (LC-MS/MS) Analyses

For the spectral library building, 5 μL of the digested peptide mixture samples were examined by liquid chromatography (LC) using an Ekspert nanoLC 425 (Eksigent Technologies, Dublin, CA, USA), which was directly connected to a mass spectrometer nanoESI qQTOF (6600 plus TripleTOF, AB SCIEX, Framingham, MA, USA) following the procedure described by Perez-Patińo et al. with minor modifications in the elution protocol [linear gradient from 7 to 40% B in A for 60 min (A = 0.1% FA; B = ACN, 0.1% FA) at a flow rate of 300 nL/min].^38^ Eluted peptides were ionized in an Optiflow source <1 μL Nano applying 3.0 kV to the spray emitter at 200 °C, and the analysis was carried out in a data-dependent mode (DDA). Ion isolation (MS1) was done with 350–1400 *m*/*z* scans for 250 ms. For the ion fragmentation and spectra generation (MS2), the quadrupole resolution was set to “LOW,” and the acquisition was done from 100–1500 *m*/*z* for 25 ms in “high-sensitivity” mode. The switch criteria used were: charge = 2+ to 4+; minimum intensity; 250 counts per second (cps). Dynamic exclusion was set to 15 s, and up to 100 ions were selected for fragmentation after each survey scan.

#### 2.6.4 Sequential Window Acquisition of All Theoretical Spectra (SWATH) Analysis of Individual Samples

For the SWATH LC-MS/MS analysis, digested samples were individually analyzed operating the TripleTOF 6600plus (SCIEX, Framingham, MA, USA) in SWATH mode. First, 5 μL of each sample was randomly loaded and examined by LC following the protocol described above.^38^ The eluted peptides were then analyzed in a mass spectrometer nanoESI qQTOF, with the tripleTOF operating in SWATH mode, in which a 0.050 s time-of-flight (TOF) MS scan from 350–1250 *m*/*z* occurred. Product ion scans were acquired after 0.080 s in 100 variable windows ranging from 400 to 1250 *m*/*z* with a total cycle time of 2.79 s.

#### 2.6.5 Protein Identification and Quantification

The SCIEX.wiff data files resulting after LC-MS/MS were processed using the paragon algorithm^39^ of proteinPilot v5.0 (AB SCIEX, Framingham, MA, USA) to search against *B. taurus* database with the following parameters: trypsin specificity, IAM Cys-alkylation, taxonomy not restricted, and the search effort set to rapid with false discovery rate (FDR) correction. To avoid having the same spectral evidence for multiple proteins, on the basis of the MS/MS spectra and regardless of the assigned peptide sequence, the protein presented as the major protein of each category was the one that could explain the spectral data with the highest confidence. The SCIEX.wiff data files obtained from the SWATH experiment were analyzed by PeakView (v 2.2, AB SCIEX, Framingham, MA, USA). The processing settings used to quantify one peptide were (1) a peptide confidence threshold of 95%, (2) six transitions per peptide, and (3) an FDR less than one percent. The identified proteins were grouped using the Protein-Pilot Group algorithm. Total protein was calculated by measuring the area under the curve (AUC) of the extracted ion chromatograms. AUCs were normalized using the total sum of the protein quantity, and the sum of all areas was made equal for all samples. The sum of all areas was equal for the entire sample. The MS proteomics data have been deposited to the ProteomeXchange Consortium via the PRIDE partner repository with the data set identifier PXD042286.

### Statistical Analyses

#### 2.7.1 Analysis to Evaluate Field Fertility, Motility and Kinetic Parameters, Mitochondrial Function, Oxidation, and Viability

The Shapiro–Wilk test was performed for assessing normality of variables. Before statistical analysis, the variables that did not present normality were transformed by calculating the arcsine of the square root or the logarithm for percentages or continuous variables. The variables were analyzed using generalized linear models (GLMs) using the SPSS Statistics v.28 program (IBM, Corp; Armonk, NY). A model with one factor (fertility: HF vs LF) was used for sperm parameters. A model with one factor (pregnancy rate) was used for field fertility groups. A probability of *p* < 0.05 was considered statistically different.

#### 2.7.2 Proteomic Data Analysis of Sperm

**Figure 2:**
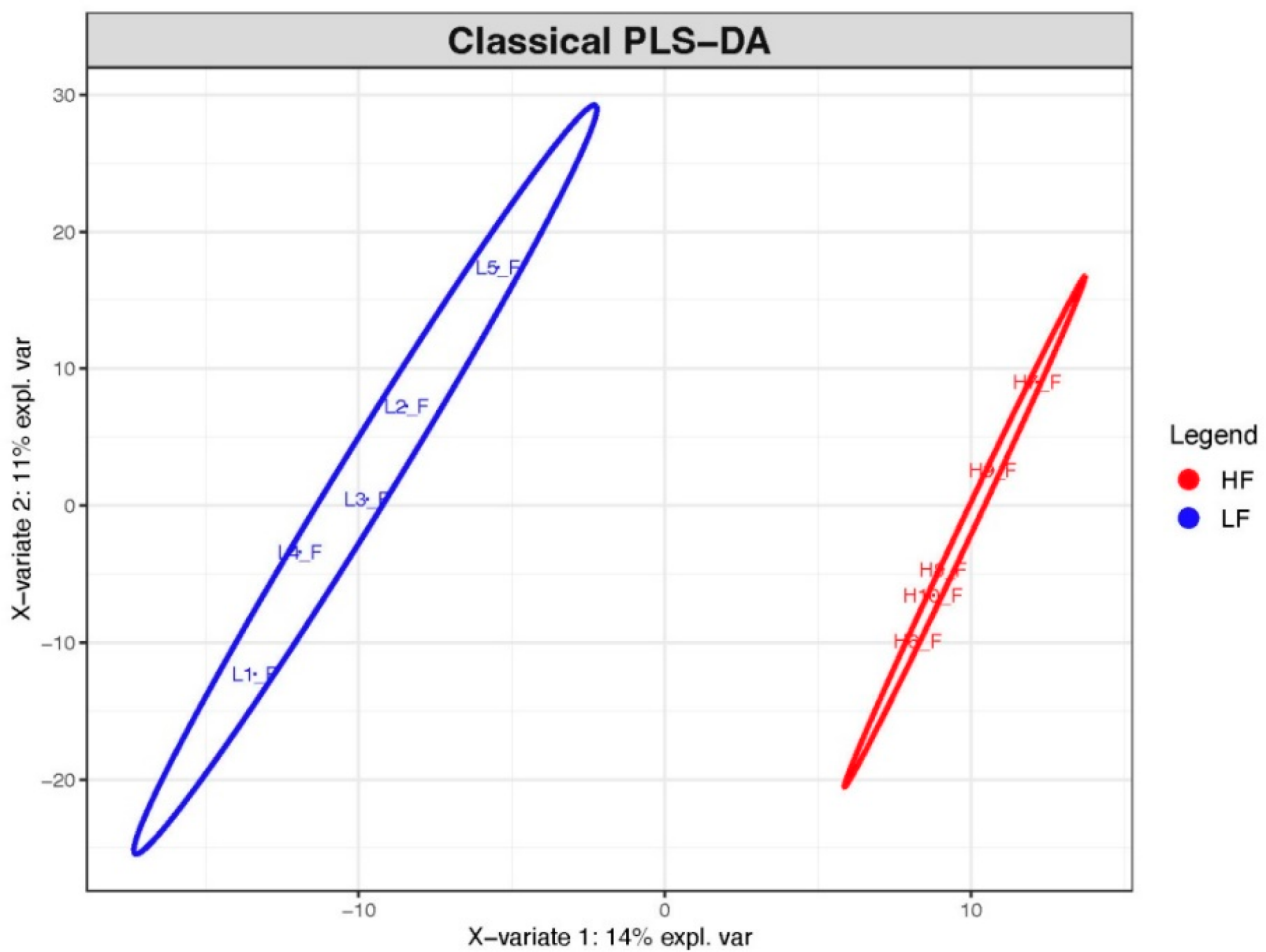
Classical partial least squares discriminant analysis (PLS-DA) of samples from the two fertility groups. H: high-fertility bulls (red); L: low-fertility bulls (blue).

The proteomics data analyses were carried out in the Statistical Unit at the University of Valencia. After proteomic assessment, the results from identified proteins were transformed by calculating log base 2. Transformed results comparing two levels of fertility were analyzed by a logistic regression model with elastic net penalty (ENLR) using the glmnet R package (version 4.1–2) with a train function of the caret package. Train function was utilized to obtain the values for parameters needed for the ENLR and suitable values of lambda and alpha (lambda = 0.232; alpha = 0.2). Moreover, the results were analyzed using the Limma package for R software and by making multiple comparisons with Benjamini and Hochberg (BH or FDR). Combination of the two methodologies was used to obtain a list of relevant differentially abundant proteins (DAPs) between fertility groups. To analyze data variation, partial least squares-discriminant analysis (see PLS-DA graphic in Figure 2) were also performed using the mixOmics package for R. A top 10 list of DAPs, corrected and ordered by *p*-value, was obtained with the Limma package (see volcano plot in Figure 4).

#### 2.7.3 Bioinformatic Analysis

The bioinformatics tool g:Profiler (https://biit.cs.ut.ee/gprofiler/) was used to perform a functional enrichment analysis.^40,41^ The tool g:GOSt analyzed over-representation of all sperm DAPs with the options of *B. taurus*, all genes known, and g_SCS significance threshold method (0.05). Moreover, the differences between high- and low-fertility males were analyzed using the multiquery option, which allows for the comparison of two sets of proteins ordered by FDR value. Kyoto Encyclopedia of Genes and Genomes (KEGG) and Reactome analyses were performed to further clarify cellular biological pathways and functions enriched in the different sperm from the HF and LF groups. Moreover, a CORUM protein database to identify the protein complex was also used to analyze DAPs. All the terms of Gene Ontology (GO) with an FDR less than 0.01 were categorized into three classes: (1) molecular process (MP), (2) cellular component (CC), and (3) biological process (BP). Analyses of the potential protein–protein interactions of the DAPs were obtained using the Search Tool for the Retrieval of Interacting Genes (STRING) software (https://string-db.org). The queries were made with the *B. taurus* database. A list of unrecognized DAPs of *Bos indicus* × *B. taurus* (hybrid) was mapped to find orthologous genes for *B. taurus* using g:Orth option and Uniprot BLAST.

## 3 Results

### 3.1 Sperm Quality *In Vitro* Parameters and Pregnancy Rate

Bulls from HF showed a significantly higher PR than those from the LF group (45.1 ± 0.5 vs 32.6 ± 0.6 for HF and LF, respectively; *p* < 0.05). Spermatozoa from the HF bull group showed a significantly higher MOT than those from the LF group (41.4 vs 29.7 for HF and LF, respectively; *p* < 0.05; Table 1). However, the PROG and kinematic parameters showed no significant difference between the HF and LF group. Results of viability, MMP, and mROS production of spermatozoa in bulls from high and low fertility are shown in Figure 1. Despite the fact that spermatozoa of the HF group showed higher rates of viability, high MMP, and lower rates of mROS, these values did not reach statistical significance (*p* > 0.05). Rates of healthy sperm (live, high MMP, and low mROS) for HF and LF bull groups were 49 and 43%, respectively, but did not reach statistical significance (*p* > 0.05).

**Table 1:** Sperm Motility and Kinematic Parameters of the HF and LF Bull Groups^a^

	bull fertility group	
sperm motility parameters	HF	LF
MOT	41.37 ± 3.55^b^	29.74 ± 5.46^b^
PROG	31.08 ± 3.39	22.17 ± 4.05
VCL	181.42 ± 6.47	172.63 ± 8.16
VSL	84.4 ± 6.07	81.45 ± 6.56
VAP	97.73 ± 5.35	93.03 ± 5.43
STR	86.03 ± 1.82	87.14 ± 2.59
LIN	46.31 ± 1.95	46.92 ± 1.87
WOB	53.74 ± 1.27	53.78 ± 0.65
ALH	4.52 ± 0.13	4.37 ± 0.22

a Mean values and standard errors (mean ± SE) of MOT = total motility (%); PROG = progressive motility (%); VCL = curvilinear velocity (μm s^–1^); VSL = straight line velocity (μm s^–1^); VAP = average path velocity (μm s^–1^); STR = straightness (%; 100 × VSL/VAP); LIN = linearity of forward progression (%; 100 × VSL/VCL); WOB = wobble (%); ALH = amplitude of lateral head displacement (μm). HF: high-fertility group; LF: low-fertility group.

b When the superscripts within each row were significantly different (*p* < 0.05).

### 3.2 Identification of Sperm Proteins

**Figure 3:**
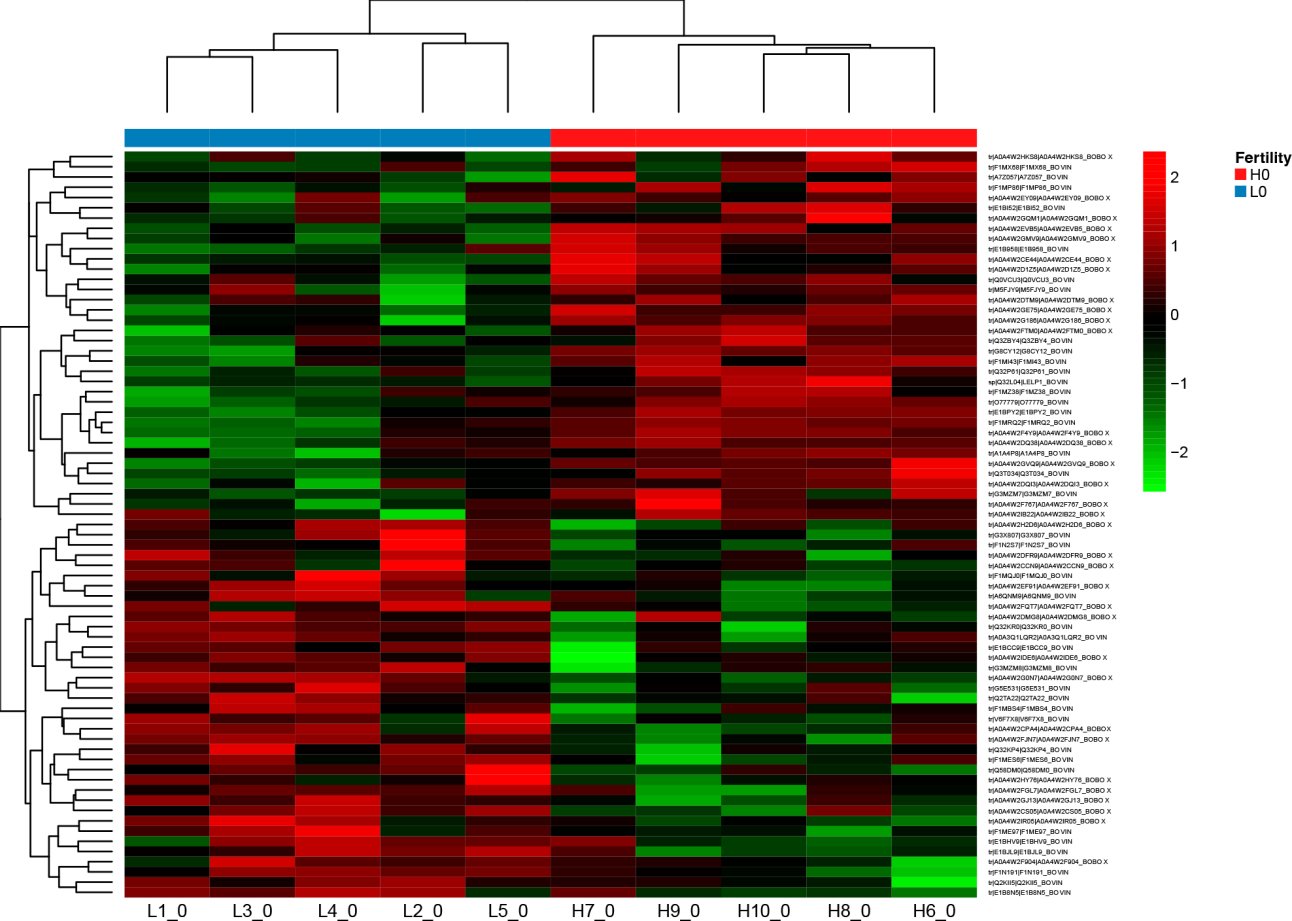
Heatmap analysis of differentially expressed sperm proteins between the high-fertility (H) and low-fertility (L) bulls.

After SWATH-MS proteomic assessment, 802 proteins were detected in sperm samples from both HF and LF bull groups (FDR < 1%), which were aligned to the *B. taurus* proteome database (see Supplementary Table S1). Partial least squares-discriminant analysis (PLS-DA) of the data revealed the sample separation in two groups, which showed samples from the LF group had more variability than the HF group (Figure 2). Statistical analysis of the proteomic data revealed 73 DAPs, of which 36 were differentially over-represented, and 37 were differentially under-represented in sperm from HF bulls compared with LF bulls (Tables 2 and 3). These 73 DAPs are presented by sample and male fertility groups in a heat map (Figure 3), which shows a protein group that is differentially expressed between HF and LF bull groups.

**Table 2:** List of the Differentially by Abundant Proteins in the High Fertility Group Obtained by Using a Multiple Logistic Regression Model with Elastic Net Penalty (lambda = 0.2 and alpha = 0.2) and the Limma Package

accession number	protein names	gene names	length Amino Acids (AA)	mass (KDa)	FC (log2)
A0A4W2IB22	leucine-rich repeat containing 74A	LRRC74A	486	54	0.28
A0A4W2HKS8	actin-like 9	ACTL9	416	46	0.29
A0A4W2EY09	glycerol-3-phosphate dehydrogenase	GPD2	727	81	0.30
A0A4W2DQI3	betaine homocysteine *S*-methyltransferase	BHMT	538	59	0.30
A0A4W2EVB5	chromosome 1 open reading frame 56		350	38	0.30
A0A4W2D1Z5	chromosome 2 open reading frame 16		5914	656	0.30
A0A4W2DTM9	phosphoglycerate kinase	PGK2	417	45	0.32
F1MZ38	succinate–CoA ligase [ADP/GDP-forming] subunit alpha, mitochondria	SUCLG1	346	36	0.32
A0A4W2GQM1	leucine-rich repeat containing 37A	LRRC37A	2532	278	0.33
E1BI52	serine protease 42	PRSS42	337	37	0.35
F1MX68	carboxypeptidase	CPVL	593	66	0.36
F1MP86	tetraspanin	TSPAN8	238	26	0.36
A0A4W2GMV9	cytochrome c oxidase subunit 7A-related protein, mitochondrial	COX7A2L	114	13	0.39
Q0VCU3	cathepsin F	CTSF	460	51	0.41
A0A4W2CE44	heat shock protein family B (small) member 9	HSPB9	157	17	0.45
A1A4P8	family with sequence similarity 209, member A (LOC784495 protein)	FAM209A	168	19	0.47
A0A4W2G186	cytochrome c	CYCS	105	12	0.52
E1B958	ALMS1 centrosome and basal body-associated protein	ALMS1	4331	482	0.59
F1MI43	sperm surface protein Sp17	SPA17	147	17	0.63
G3MZM7	protein phosphatase inhibitor 2	IPP2	520	56	0.78
O77779	fertilin alpha	ADAM 1	812	90	0.80
A0A4W2DQ38	IQ motif containing F1	IQCF1	209	24	0.86
Q32L04	late-cornified envelopelike proline-rich protein 1	LELP1	110	12	0.92
G8CY12	beta-defensin	DEFB	89	9	0.93
Q3T034	serine-rich single-pass membrane protein 1	SSMEM1	241	28	0.96
A0A4W2F767	transmembrane and coiled-coil domains 2	TMCO2	179	20	0.97
Q32P61	histone H2A	H2AL1Q	117	13	1.02
A0A4W2GE75	tubulin polymerization-promoting protein family member 2	TPPP2	171	19	1.21
A0A4W2FTM0	diazepam-binding inhibitorlike 5	DBIL5	87	10	1.31
A7Z057	14-3-3 protein gamma	1433G	247	28	1.38
A0A4W2GVQ9	sperm-associated antigen 16	SPAG16	628	70	1.46
Q3ZBY4	fructose-bisphosphate aldolase	ALDOC	364	39	1.48
A0A4W2F4Y9	cytochrome c oxidase subunit 7C	COX7C	63	7	1.72
F1MRQ2	chromosome 3 C1orf185 homologue	C3H1orf185	208	24	1.92
E1BPY2	equatorin	EQTN	133	15	1.98
M5FJY9	phosphoglycolate phosphatase	PGP	321	34	2.20

**Table 3:** List of the Differentially by Abundant Proteins in the Low Fertility Group Obtained by Using a Multiple Logistic Regression Model with Elastic Net Penalty (lambda = 0.2 and alpha = 0.2) and Limma Package

accession number	protein names	gene names	length AA	mass (KDa)	FC (log2)
Q2KII5	histone H2B	H2B	126	14	–2.55
A0A4W2FGL7	family with sequence similarity 162 member A	FAM162A	156	18	–1.54
A0A4W2IDE6	UBC core domain-containing protein		147	17	–1.48
A0A4W2CCN9	apolipoprotein A-I	APOA1	207	24	–1.34
A0A4W2GJ13	14-3-3 protein theta	1433T	421	47	–1.31
Q32KR0	TBC1 domain family, member 21	TBC1D21	299	35	–1.27
G3 × 807	histone H4	H4C9	98	11	–1.22
A0A4W2IR05	outer dynein arm-docking complex subunit 4	ODAD4	683	78	–1.20
F1MBS4	solute carrier family 25-member 10	SLC25A10	287	32	–1.11
E1BCC9	small nuclear ribonucleoprotein Sm D1 (snRNP core protein D1)	SNRPD1	134	15	–1.10
A0A4W2H2D6	proteasome subunit beta	PSMB4	264	29	–1.06
A0A4W2DMG8	heat shock protein 90 beta family member 1	HSP90B1	808	93	–1.04
A0A4W2CPA4	regulator of G-protein signaling 22	RGS22	1254	146	–0.98
A0A4W2FJN7	NADH dehydrogenase [ubiquinone] 1 beta subcomplex subunit 8, mitochondrial	NDUFB8	186	22	–0.91
A0A4W2G0N7	heat shock protein 90 alpha family class A member 1	HSP90AA1	776	89	–0.86
V6F7 × 8	oligoribonuclease, mitochondrial	REXO2	237	27	–0.83
F1MQJ0	Angiotensin-converting enzyme (ACE)	ACE DCP1	1306	150	–0.83
F1MES6	UBX domain-containing protein 6	UBXN6	441	50	–0.82
A0A4W2HY76	chaperonin-containing TCP1 subunit 6B	CCT6B	518	57	–0.79
A0A3Q1LQR2	tubulin alpha chain	LOC112443216	449	50	–0.78
A0A4W2DFR9	ferritin		157	18	–0.70
E1BJL9	cilia- and flagella-associated protein 210	CFAP210	547	65	–0.67
A0A4W2F904	dynein axonemal intermediate chain 7	DNAI7	721	84	–0.61
F1ME97	5-oxoprolinase	OPLAH	1210	129	–0.61
A0A4W2EF91	eppin-like		138	16	–0.59
A0A4W2CS05	calmodulin-binding transcription activator 1	VAMP3	148	16	–0.57
E1BHV9	ornithine decarboxylase antizyme 3	OAZ3	117	14	–0.54
A6QNM9	SLC25A12 protein (solute carrier family 25 member 12)	SLC25A12	675	75	–0.54
A0A4W2FQT7	proteasome subunit alpha type	PSMA4	261	29	–0.52
Q58DM0	isocitrate dehydrogenase [NAD] subunit, mitochondrial	IDH3G	392	43	–0.46
Q32KP4	abhydrolase domain containing 16B	ABHD16B	470	53	–0.41
Q2TA22	long-chain-fatty-acid–CoA ligase	ACSL6	697	78	–0.41
F1N191	interleukin 4-induced 1	IL4I1	578	64	–0.40
G3MZM8	vitamin-K-epoxide reductase	VKORC1L1	176	20	–0.38
F1N2S7	testis-specific serine kinase 6	TSSK6	273	30	–0.32
G5E531	T-complex protein 1 subunit alpha	TCP1	556	60	–0.29
E1B8N5	sodium/potassium-transporting ATPase subunit alpha	ATP1A4	1030	114	–0.13

**Figure 4:**
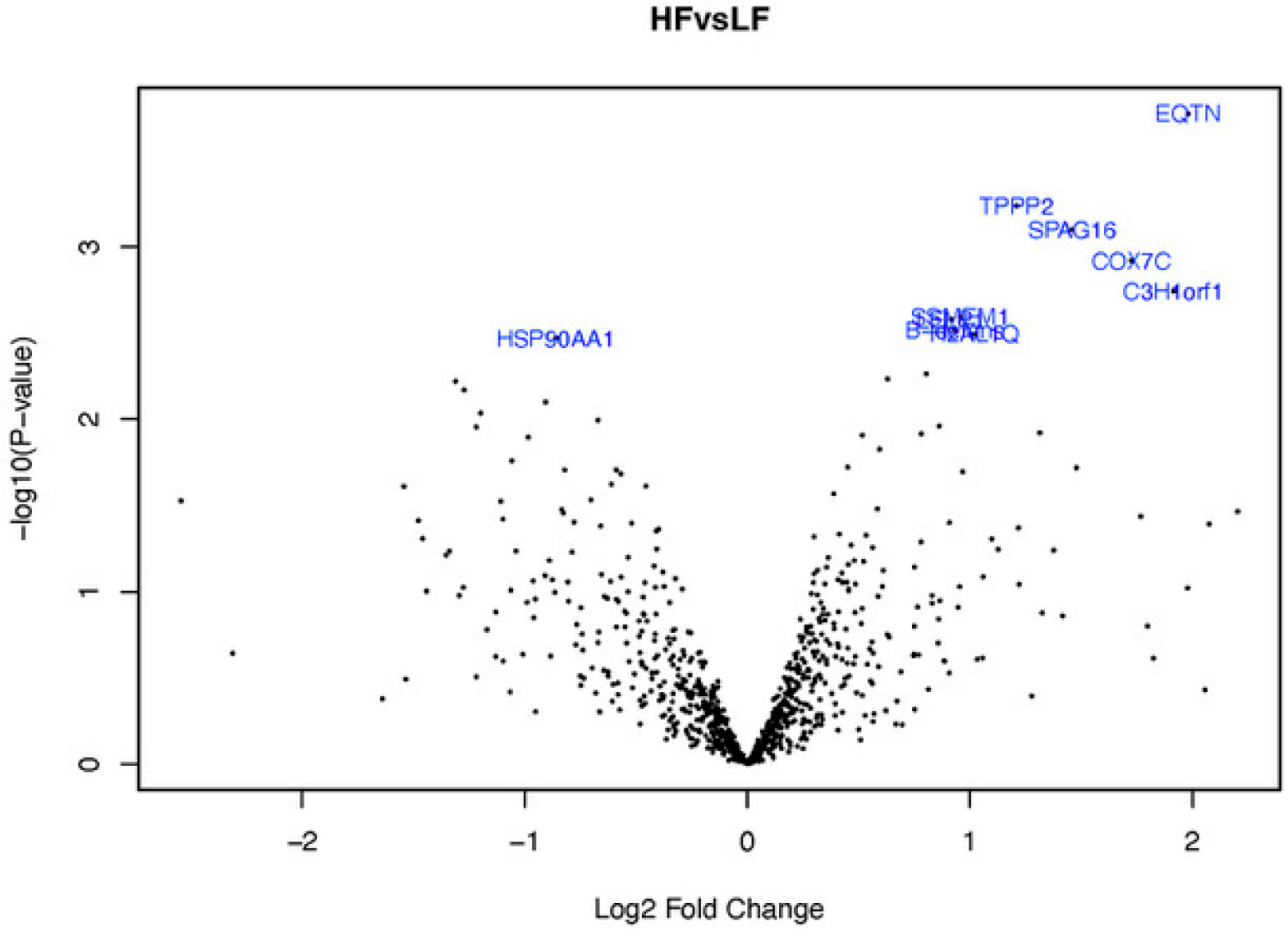
Volcano plot of the sperm proteins of both fertility groups. The *X* axis represents the logarithm of the fold change HF/LF representing the biological impact (log2 fold change), and the *Y* axis logOdds (B-statistic value) represents the statistic impact (−log10 *P* value; cut off ≥ 1.3).

The top ten DAPs in HF and LF bulls are shown in Table 4 and Figure 4. In the HF group, we found an over-representation of the proteins equatorin (EQTN), tubulin polymerization-promoting protein family member 2 (TPPP2), sperm-associated antigen 16 (SPAG16), cytochrome c oxidase subunit 7C, mitochondrial (COX7C), chromosome 3 C1orf185 homologue (C3H1orf185), serine-rich single-pass membrane protein 1 (SSMEM1), late-cornified envelopelike proline-rich protein 1 (LELP1), beta defensin (DEFB), and histone H2A (H2AL1Q). In contrast, in the LF group, only heat shock protein HSP 90-alpha (HSP90AA1) protein was overrepresented.

**Table 4:** List of the Top 10 Differentially Expressed Proteins and Their Functional Role in High- and Low-Fertility Bull Sperm According to UniProt KB Database^a^

bull fertility group	gene names	protein names	function
HF	EQTN	equatorin	involved in single fertilization: fusion of spermatozoa to the oocyte plasmatic membrane
HF	TPPP2	tubulin polymerization-promoting protein family member 2	nonmotor microtubule binding protein
HF	SPAG16	sperm-associated antigen 16	sperm flagellum, sperm axoneme assembly
HF	COX7C	cytochrome c oxidase subunit 7C, mitochondrial	electron transport chain of mitochondria, cytochrome c to oxygen
HF	C3H1orf185	chromosome 3 C1orf185 homologue	
HF	SSMEM1	serine-rich single-pass membrane protein 1	
HF	LELP1	late-cornified envelopelike proline-rich protein 1	
HF	DEFB	beta-defensin	defense response to bacterium
HF	H2AL1Q	histone H2A	chromatin/chromatin-binding or regulatory protein
LF	HSP90AA1	heat shock protein HSP 90-alpha	protein folding chaperone, hydrolase, ATP-dependent activity

a UniProt KB Database (www.uniprot.org).

### Gene Ontology Analysis

#### 3.3.1 Biological Processes, Molecular Function, and Cellular Components

**Figure 5:**
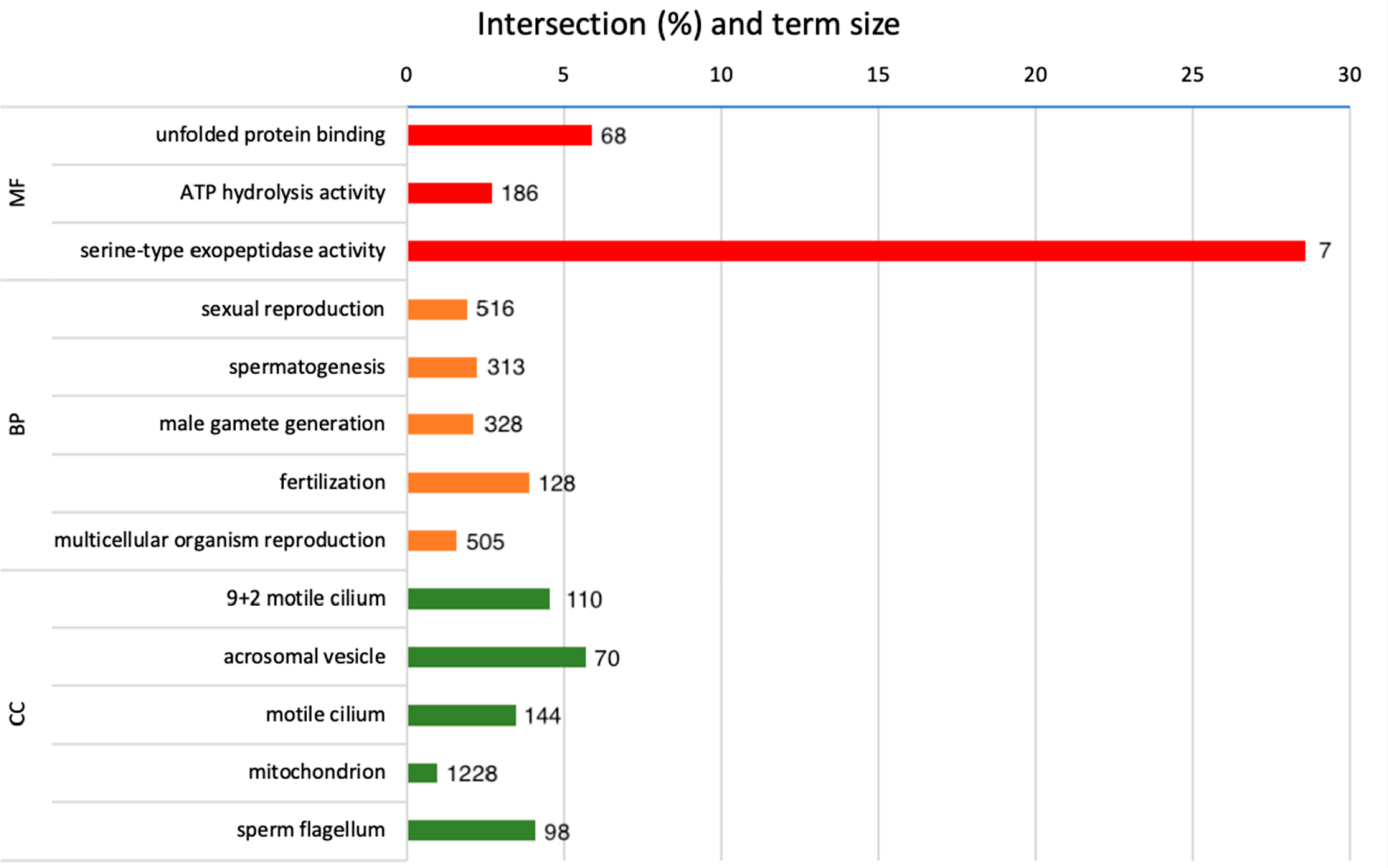
g:Profiler test recognized differentially abundant proteins identified by SWATH and gene ontology analysis of high- and low-fertility (*Bos taurus*) spermatozoa (released 18-02-2023). Gene ontology terms are MF, molecular function (red); BP, biological process (orange); and CC, cellular component (green).

**Figure 6:**
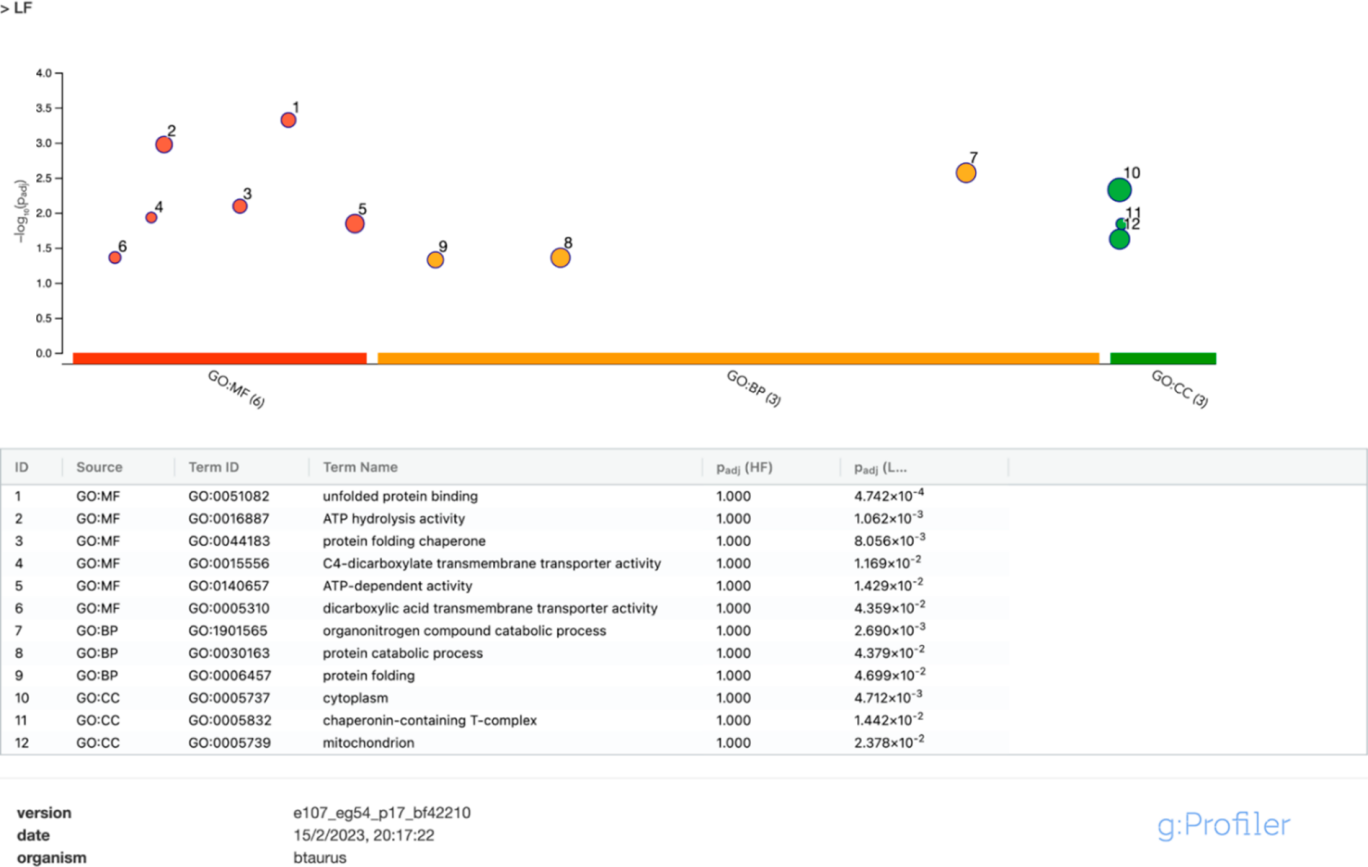

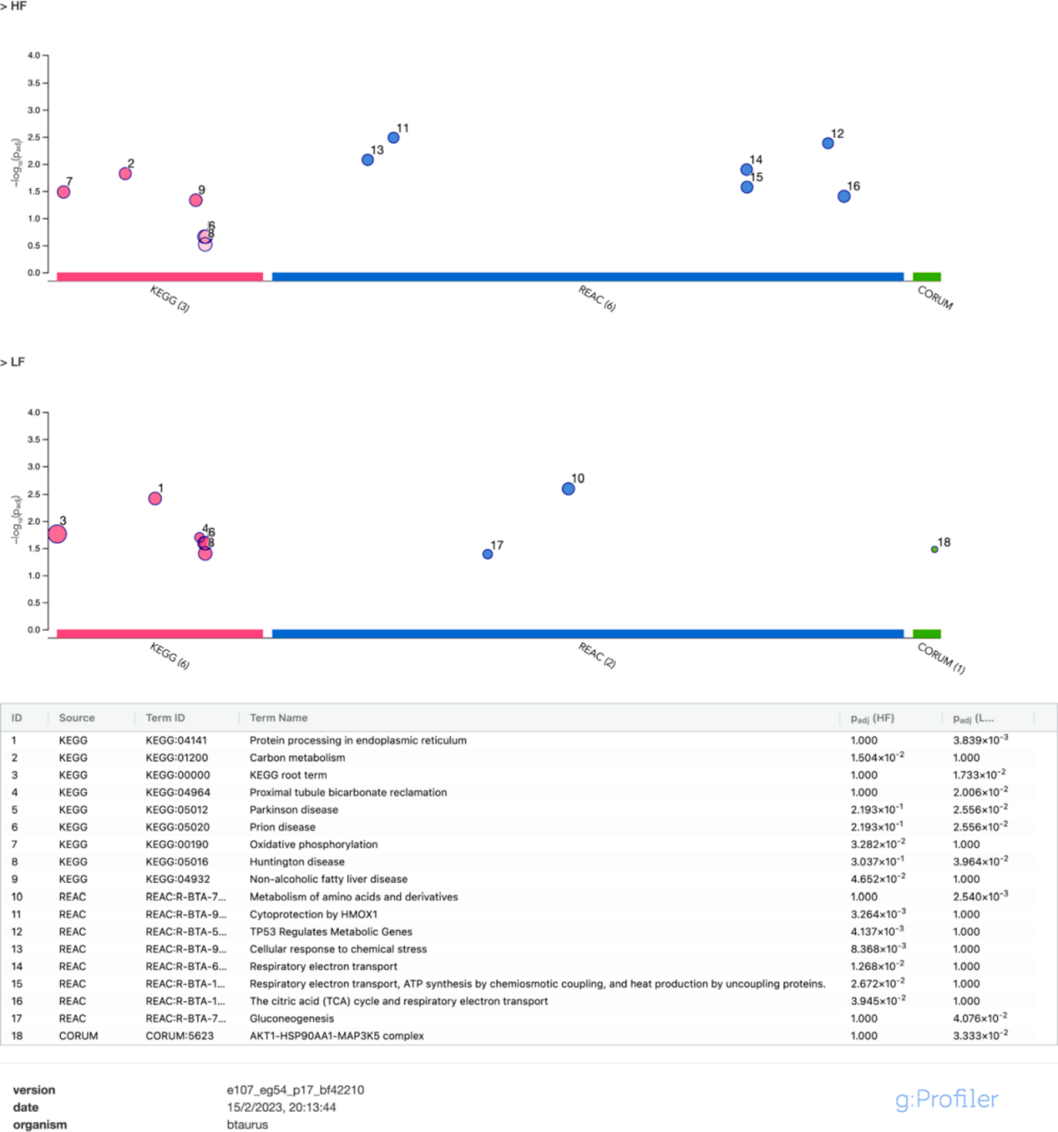
g:GOST multiquery Manhattan plot showing comparative enrichment analysis of bull sperm, differentially abundant proteins obtained from low (LF)- and high (HF)-fertility bulls (released 18-02-2023). The sperm proteomes from each fertility group were queried against the *Bos taurus* proteome database. (A) Gene Ontology (GO) terms: molecular function (MF) is in red, biological process (BP) is in orange, and cellular component (CC) is in green for the low-fertility group. (B) KEGG pathway is in pink, and the Reactome pathway is in blue for LF and HF groups. CORUM protein complexes are in green.

Gene Ontology (GO) analysis results of DAPs are shown in Table 5 and Figure 5. Enriched terms for the BP of DAPs were sexual reproduction, spermatogenesis, male gamete generation, fertilization, and multicellular organism reproduction (Table 4). GO terms for molecular functions (MFs) overrepresented were ATP hydrolysis activity, unfolded protein binding, and serine-type exopeptidase activity (Table 5). For CCs, GO terms overrepresented were related with motility-related structures (9 + 2 motile cilium, motile cilium, and sperm flagellum), acrosomal vesicle, and mitochondrion. After analyzing the DAPs separately by fertility groups, GO terms for MF, BP, and CC were only significantly enriched in the LF group (Figure 6A). The terms that were enriched for LF bulls for MF were unfolded protein binding, ATP hydrolysis and dependent activity, protein folding chaperone mostly enriched by protease, and dicarboxylic acid transmembrane transporter activity. For BPs, LF was enriched by organonitrogen compound catabolic process and protein folding and catabolic process. The GO terms cytoplasm, mitochondrion, and chaperonin-containing T complex were enriched for CC.

**Table 5:** Gene Ontology (GO) Analysis by g:Profiler Software of the Differentially Abundant Proteins of High- and Low-Fertility (*Bos taurus*) Spermatozoa (Released 18-02-2023), With Proteins Categorized on the Basis of Their Molecular Function, Their Role in the Biological Process, and Cellular Component

GO source	GO term_name	id	adjusted *p* value
molecular function	unfolded protein binding	51082	0.009
	ATP hydrolysis activity	16887	0.038
	serine-type exopeptidase activity	70008	0.048
biological process	sexual reproduction	19953	0.001
	spermatogenesis	7283	0.018
	male gamete generation	48232	0.024
	fertilization	9566	0.024
	multicellular organism reproduction	32504	0.049
cellular component	9 + 2 motile cilium	97729	0.002
	acrosomal vesicle	1669	0.006
	motile cilium	31514	0.007
	mitochondrion	5739	0.010
	sperm flagellum	36126	0.024

#### 3.3.2 KEGG, Reactome Pathways, and CORUM

Results of KEGG pathways, Reactome analysis, and CORUM are shown in Figure 6B. Regarding KEGG pathways, carbon metabolism and oxidative phosphorylation were significantly overrepresented in bull semen samples from the HF group, among others, but not for the LF group. However, protein processing in the endoplasmic reticulum KEGG pathway was significantly overrepresented in the LF group but not for the HF group. Concerning Reactome pathways, the LF group was significantly enriched in gluconeogenesis and metabolism of amino acids and derivatives pathways compared with the HF group. However, the HF group was significantly overrepresented in respiratory electron transport (respiratory electron transport; respiratory electron transport; ATP synthesis by chemiosmotic coupling and heat production by uncoupling proteins; the citric acid cycle and respiratory electron transport) and stress response (cytoprotection by HMOX1; cellular response to chemical stress).

### 3.4 String protein–protein interaction

**Figure 7:**
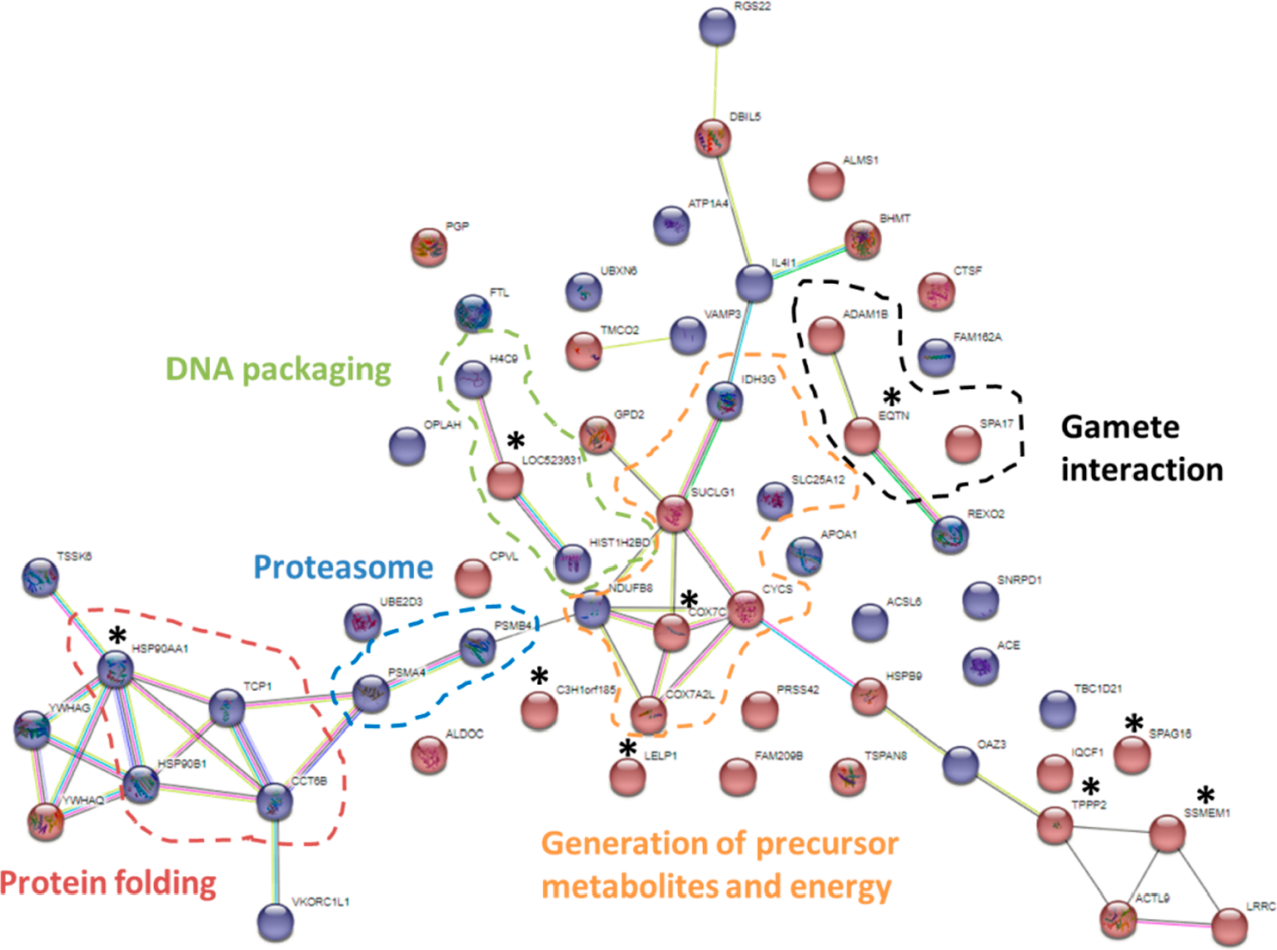
STRING protein–protein interaction network showing the interactions of the differentially abundant proteins in *Bos taurus* sperm of high- and low-fertility bulls. Proteins are linked by confidence network edges, which show putative protein interactions. The red plot belongs to the high-fertility group; the blue plot belongs to the low-fertility group. The top 10 proteins were identified with an asterisk. The proteins having related functions were grouped in discontinued circles: DNA packaging in green, gamete interaction in black, generation of precursor metabolites and energy in orange, proteasome in blue, and protein folding in red.

After analyzing DAPs with STRING software, a total of 62 nodes and 47 edges were identified in *B. taurus* spermatozoa (Figure 7). Among significant BPs, those with the highest strength values were cellular respiration, electron transport chain, generation of precursor metabolites, and energy. To identify DAPs of different fertility groups, DAPs for the HF and LF group were represented in red and blue, respectively. Five protein clusters with biological or cellular functions were defined: (1) protein folding, (2) proteasome, (3) generation of precursor metabolites and energy, (4) gamete interaction, and (5) DNA packaging. Moreover, the top 10 proteins are identified with an asterisk in Figure 7.

## 4 Discussion

Predicting the fertility potential of males or seminal samples is a challenge that has not yet been resolved. Despite improvements in recent years in the *in vitro* analysis of semen, both in the standardization and inclusion of multiple variables in the mathematical models, the predictive capacity of these *in vitro* assessments continues to be limited.^42^ On the one hand, both CASA and FC procedures allow us to analyze a large number of spermatozoa in a more objective way in order to explain part of fertility variation.^7^ On the other hand, proteomic analyses allow us to identify potential biomarkers associated with sperm quality and fertility. Several previous studies have used a DDA method for proteomic data collection;^21−23^ however, few fertility markers identified were common in all of them. These discrepancies could be due to different animal samples, different procedures, or the data reproducibility of procedures. In fact, the lack of reproducibility in analytical procedures is a challenge that scientists must face. As previously noted, SWATH-MS method allows us to collect data using a DIA method that presents more sensitivity (more quantified proteins) and reproducibility compared with DDA-based methods and targeted proteomics.^29,30^ Recently, Barkovits et al. found that DIA methods showed greater reproducibility and accuracy than DDA methods^43^ in terms of protein quantification, especially when a sample had low protein amounts. For all the above reasons, we analyze *in vitro* parameters evaluated by CASA and FC and proteome evaluated by SWATH-MS of bull spermatozoa cataloged as high- and low-field.

The use of CASA and FC systems to assess sperm functionality and their link with fertility has been widely studied in bulls and in different species.^7,11,12,15,44^ However, no consistent results were obtained among different studies.^13^ Regarding CASA parameters, only sperm MOT was significantly different between HF and LF bulls, with it being higher in HF males. This result is in line with some other research studies^11,45,46^ but not with others.^12,47^ Kinetics of sperm motility, such as velocities, were similar in both fertility groups.^11,12,14,46,48^ Motility is one of the most important parameters in evaluating sperm quality. Sperm motility is generated by flagellum movement with high energy consumption after ATP hydrolysis. Thus, motility can be understood as a result of a complex process involving both flagellum movement and ATP hydrolysis, cell and organelle membrane integrity, and adequate oxidative status. In this sense, we found that HF bull sperm showed higher viability and higher hMMP and lower mROS; however, these differences did not reach statistical significance. Nonsignificant differences in various sperm parameters are sometimes explained by little difference in bull fertility rates^14,49^ or a low number of AI per bull.^14^ However, we found that field fertility rates from HF and LF bulls differed significantly in more than 12 percentage points (45.1% for HF and 32.6% for LF), and the minimum number of AIs per bull was 1264. Our study agrees with other research that has found no differences in viability^12,22,46^ or hMMP^11,12,22,48^ or mROS.^14,22,48^ In contrast, some other studies found significant differences between fertility groups in viability.^11,14,31,48^ Since fertility is a multiparametric process, a single sperm quality parameter is not sufficient to assess the overall fertility potential of a semen sample. This justifies the combined study of multiple parameters, including protein biomarkers.

By using the SWATH-MS technique, we identified 802 proteins, of which 73 were DAPs between high- and low-fertility bulls whose differential analysis points to functional differences that could lead to the identification of fertility markers. Our results showed an enrichment of metabolic pathways related to energy metabolism, especially the oxidative phosphorylation process in the HF group, as well as biological processes overrepresented in the LF group related to protein processing, catabolism, and protein folding. To be functional in the fertilization process, mammalian sperm depend on the proper functioning of different biological processes, such as motility, capacitation, hyperactivation, and gamete interaction, including sperm–zona pellucida, acrosome reaction, and sperm–oocyte plasma membrane adhesion to fusion. Since all of these processes are ATP-dependent, energy production mechanisms are important for the normal male gamete function. For ATP production in bovine sperm, both oxidative phosphorylation (OXPHOS) and anaerobic glycolytic pathways are implicated.^50^ The OXPHOS process takes place in the inner mitochondria membrane and involves the respiratory chain and ATP synthase,^51^ which provides 15 times more ATP than glycolysis. This energetic process seems to play a key role in the capacitation process; in fact, bull spermatozoa depend on OXPHOS to support capacitation.^51,52^ Motility is another important and energy-intensive process in fertilization. However, the predominant mechanism for ATP production is not clear and seems to be species-dependent: species like human, sheep, or cattle support motility with glycolysis, but other species like boar or horse mainly use OXPHOS.^53−55^ Tourmente and colleagues,^56^ showed a direct relationship between the respiratory activity, the sperm ATP content, and the improvement of different motility parameters. Additionally, MMP is described as a sperm motility indicator, and its reduction diminishes sperm motility and fertilization capacity.^51^ In HF bulls, our proteomic results showed an enrichment in proteins related to the OXPHOS pathway that, together with the observed increasing trend of the hMMP, could partially explain the improvement in sperm motility; this could confer a functional advantage to HF over LF bulls in their fertility capacity. Other studies found similar results, although with different proteins. D’Amours et al. found the most of the DAPs in HF bull sperm were related to energy metabolism, specifically, the glycolytic pathway member TPI1; adenylate kinase-8, which is involved in ATP production; the transaminase OAT, which enables ATP production through the entry of its product (glutamate) into the tricarboxylic citric acid cycle; and the protein DBIL5, which has been shown to have structural and binding similarity to acetyl CoA binding protein (ACBP).^22^ In addition, Saraf et al. found that OXPHOS and the citrate cycle were the most significantly affected pathways in low-fertility crossbred bull sperm.^27^

The hallmarks in LF bull spermatozoa are isoforms of the chaperone HSP90 (HSP90AA1 and HSP90B1), members of the chaperonin containing the TCP1 complex, and components of the 20S core proteasome complex (PSMA4 and PSMB4). These results could probably indicate defects in protein folding and proteostasis for this group. In fact, the only top 10 protein differentially expressed in the LF group was HSP90AA1. Protein turnover regulation is crucial to maintain protein homeostasis in cells. Under stress, the cell ability to fold proteins can be overwhelmed by the high levels of unfolded or misfolded proteins, and this, generally, culminates in the transcription of molecular chaperones, which assist in the proper folding of proteins.^57^ Heat shock protein 90 (HSP90) is a chaperone that facilitates the final folding of client proteins. This protein has two major cytoplasmic isoforms: HSP90A (inducible) and HSP90B (constitutive). HSP90 has been shown to play a role in the regulation of motility and capacitation of spermatozoa.^58,59^ Pharmacological inhibition of HSP90 reduces porcine sperm motility,^60^ and its loss during the freezing correlates with a diminution in motility of sperm in bovine.^61^ Kasimanickam et al. found that HSP90 was overexpressed in HF bull sperm,^24^ whereas our results showed a negative association between the overexpression of HSP90 isoforms and motility in the LF group. These results are in line with those found by D’Amours and colleagues who described a significant abundance of HSP90AA1, HSPA2, and TCP1 in spermatozoa of low-fertility bulls.^22^ We can hypothesize that this increase in chaperone protein levels in LF bulls, particularly in the inducible form of HSP90 together with the increase of some proteasomal proteins (which could indicate an increase in protein degradation), could be due to the presence of damage or misfolded proteins that may be affecting spermatozoa functionality. Thus, further investigations are needed to clarify the role of chaperones in male fertility.

Analysis of the top 10 DAPs revealed that while nine proteins were more abundantly expressed in the HF group (related to capacitation, motility, energy production, and DNA packaging processes), only one protein was more differentially expressed in LF bulls, and it was related to protein folding deficiencies. Sperm motility is a fertility parameter directly linked to fertilization success that is mainly dependent on normal energy production and its specific structures. Our results show in the list of the top 10 DAPs the presence of proteins related to both energy production (COX7C) and to the development of structures linked to the motility process [tubulin polymerization-promoting protein 2 (TPPP2); serine-rich single-pass membrane protein 1 (SSMEM1); and sperm-associated antigen 16 (SPAG16)]. Cytochrome oxidase subunit c (COX7C) is a protein that takes part in the final stage of the electron transport chain that results in the generation of ATP in the inner mitochondrial membrane. Recent evidence has shown that sperm motility in bulls may be affected by the disruption of the mitochondrial electron transport chain function.^62^ Additionally, Card et al. found an abundant expression of transcripts for COX7C in frozen–thawed semen from low-fertility bulls and hypothesized that this abundant expression might be correlated with a transcript′s inefficient translation, which in turn would affect mitochondrial function during the latter stages of spermatogenesis.^63^ TPPP2 is one of the three family members of the tubulin polymerization-promoting proteins (TPPPs), and it has been found that it is expressed in the middle piece of the human mature sperm tail. Its blockage decreases sperm motility by affecting energy production but not the capacitation or the acrosome reaction.^64^ Ultrastructure analysis of sperm from Tppp2–/– mice has shown normal microtubules and outer dense fibers but an impaired mitochondria structure with a significantly increased proportion of sperm lacking inner mitochondrial membrane cristae.^64^ Moreover, a recent study of phosphorylated proteins in capacitated sperm has shown that this post-translational modification of TPPP2 may also be involved in the acquisition of sperm fertilization capacity.^65^ SSMEM1 is a transmembrane protein found in the human^66^ and mice^67^ testis, but to our knowledge it has not been described in mature spermatozoa, nor is its function clear yet; Nozawa et al. found that KO mice lacking SSMEM1 are sterile because of abnormal sperm head morphology and reduced motility.^67^ SPAG16 is considered a tissue-specific gene expressed primarily in the testis and tissues with flagellated cells or motile cilia.^68^ It is a critical structural component of motile cilia and flagella essential for normal spermatogenesis and sperm motility.^69^ It has been reported that the loss of axonemal central apparatus proteins would be associated with severe sperm motility defects.^70^

Another important process related to fertility is the sperm–oocyte interaction, in which several proteins are involved. Equatorin (EQTN) is a transmembrane protein located in plasma and inner and outer acrosomal membranes that is likely involved in sperm–oocyte adhesion^71^ and acrosomal reaction.^72^ In mice, EQTN deficiency in spermatozoa showing normal motility and morphology was related to low fertility^71,72^ However, porcine spermatozoa of low prolificity showed higher levels of EQTN.^73^ We found no information about EQTN in similar proteomic studies comparing different bull fertility. Moreover, we observed that sperm fertilin α (ADAM1)^20^ and autoantigen protein 17 (SPA17) were overrepresented in HF bulls and that these have a role on sperm–plasma membrane adhesion and binding to zona pellucida during fertilization.^74−76^

During spermiogenesis, nucleosome-core histones H2A, H2B, H3, and H4 are first replaced by transition proteins and then by leaving tightly compacted sperm DNA, which forms a structure called toroidal model.^77^ Despite the major presence of protamines in the sperm nucleus, a histone retention rate of about 15% in humans and 1 to 10% in mice has been described in mature sperm,^78^ which provides less condensed regions where the accessibility of transcription factors to paternal genes would be higher after fertilization.^79^ Thus, while proper histone retention is vital for gene activation in early embryonic development, excessive histone retention points to sperm chromatin immaturity, which can lead to sperm dysfunction and male infertility.^80^ Our proteomic results have shown an overexpression of the histone H2A in the HF group and of H2B and H4 in the LF group. In contrast, other proteomic analyses found an overexpression of the linker histone H1 in HF bull spermatozoa,^24^ and other works found no relationship between bull fertility and expression levels of histones H2B, H3.3, and H4.^81^ Although the specific contributions of these histones to reproduction is not well known, it has been shown that knockout mice with a variant of histone H2A are subfertile and have a high proportion of abnormal sperm^82^ Similarly, H2B variants have been shown to be higher in the spermatozoa of infertile men.^83^ Additionally, normal sperm function and fertility seem to be affected by histone post-translational modifications (PTMs), such as phosphorylation, methylation, or acetylation. Schon et al. showed that sperm with abnormalities in motility or morphology had differences in the amount of histone PTMs compared to normozoospermic sperm samples,^84^ and Kutchy et al. demonstrated that alterations in histone H3 methylation or acetylation were associated with bull infertility.^85^ So, further proteomic enrichment studies to identify histone PTMs could be of translational importance to describe their relationship to alterations in fertility.

DEFB is another of the top 10 proteins overrepresented in the HF group. DEFBs are secreted as host defense peptides with antimicrobial, antifungal, and antiviral functions. It has been reported that DEFBs are secreted in mammalian epididymis and bind to the sperm plasma membrane to form sperm glycocalyx.^86^ Additionally, DEFB-15 has been reported to have a critical role in the rat sperm motility;^87^ DEFB126 has been associated with both sperm motility and fertility in humans^86^ and bovine,^88^ and the porcine DEFB129 has been found to play a role in the capacitation process.^89^ All of this evidence suggests that some members of this protein family are involved in important sperm functional activities, thus making them suitable molecular markers for male fertility.

Taken together, our results describe novel potential biomarkers of bull fertility with important roles in mature and functional sperm. In line with our results, Pang et al. describe that a combination of EQTN and zona pellucida sperm-binding protein (ZP4) would be a fertility prediction model,^90^ and other proteomic studies have identified other proteins, such as protamine 1 (PRM1), outer dense fiber of sperm tails 2 (ODF2), and postacrosomal assembly of sperm head protein (PAWP), as potential fertility markers.^91,92^ However, further studies are needed to define the relationship between the proteome and sperm fertility and to quantify and clarify how these specific proteins related to sperm physiology are true markers of male fertility in bovine species.

## 5 Conclusion

In the search for biomarkers, it is crucial to ensure that the evaluation of physiological parameters and biological processes is objective and reproducible. In this work, we evaluated different sperm parameters using CASA, FC, and proteome using SWATH-MS, with the aim of enhancing the reliability and reproducibility of the results. Our investigation revealed that spermatozoa from HF bulls showed higher total motility and greater abundance of proteins linked to both energy production (mainly the OXPHOS pathway) and structures related to the motility process. Furthermore, we observed that EQTN, along with other proteins related to the interaction with the oocyte, was overrepresented in the spermatozoa of HF bulls. However, the biological processes related to protein processing, catabolism, and protein folding were overrepresented in LF bull sperm. In addition, enrichment in chaperone HSP90 was observed. Given that various metabolic pathways in sperm could influence fertility, the identification of a single biomarker to explain variability in fertility among males is nowadays challenging. Therefore, the optimization of mathematical models incorporating multiple variables, including physiological parameters and proteins associated with different biological processes, must be considered to be essential to elucidating this variability.

## Supporting Information Available

Supplementary Table S1: Full list of the 802 identified proteins after the proteomic analysis by SWATH LC/MS-MS of the sperm samples of high- (H) and low- (L) fertility bulls (PDF)

## Abbreviations

HF: high fertility
LF: low fertility
MMP: mitochondrial membrane potential
mROS: mitochondrial reactive oxygen species
DAP: differentially abundant proteins
AI: artificial insemination
CASA: computer-assisted sperm analysis
FC: flow cytometry
DIA: data-independent acquisition
DDA: data-dependent acquisition
SWATH-MS: sequential window acquisition of all theoretical mass spectra
TL: Tyrode’s medium
TM: total motility
PM: progressive motility
VCL: curvilinear velocity
VSL: straight line velocity
VAP: average path velocity
LIN: linearity of forward progression
STR: straightness
WOB: wobble
ALH: amplitude of lateral head displacement
DAPI: 4′,6′-diamidino-2-phenylindole
MTDR: Mitotracker Deep Red
MSOX: Mitosox Red
TFA: trifluoroacetic acid
ACN: acetonitrile
FA: formic acid
LC-MS/MS: liquid chromatography and tandem mass spectrometry
BP: biological process
CC: cellular component
MP: molecular process
PROG: progressive motility sperm
FDR: false discovery rate
OXPHOS: oxidative phosphorylation pathway
HSP90AA1: heat shock protein-alpha 90
EQTN: equatorin
TPPP2: tubulin polymerization-promoting protein 2
SSMEM1: serine-rich single-pass membrane protein 1
SPAG16: sperm-associated antigen 16
COX7C: cytochrome oxidase subunit c
LELP1: late-cornified envelopelike proline rich 1
DEFB: beta defensing
H2AL1Q: histone H2A
PR: pregnancy rates
