# Supplementary material for: Bull Sperm SWATH-MS-Based Proteomics Reveals Link between High Fertility and Energy Production, Motility Structures, and Sperm–Oocyte Interaction
Source: J Proteome Res. 2023 Oct 2;22(11):3607–24. doi: 10.1021/acs.jproteome.3c00461 (PMC10629479; doi:10.1021/acs.jproteome.3c00461)
Supplement: Supplementary file 1 — pr3c00461_si_001.pdf [file pr3c00461_si_001.pdf]

Bull sperm SWATH-MS-based proteomics reveals link between high fertility and energy production, motility structures, and sperm-oocyte interaction

Sabrina Gacem[1,2], *María Castello-Ruiz*[1,3]*, *Carlos O. Hidalgo*[4], *Carolina Tamargo*[4], *Pilar Santolaria*[5], *Carles Soler*[1], *Jesús L. Yániz*[5], *Miguel A. Silvestre*[1]

[1]Departamento de Biología Celular, Biología Funcional y Antropología Física, Universitat de València, 46100 Valencia, Spain. E-mail: maria.castello@uv.es

[2]Departamento de Medicina y Cirugía Animal. Universitat Autònoma de Barcelona, 08193 Barcelona, Spain.

[3]Unidad Mixta de Investigación Cerebrovascular, Instituto de Investigación Sanitaria La Fe, Hospital Universitario y Politécnico La Fe, 46026 Valencia, Spain.

[4]Animal Selection and Reproduction Area, Regional Agrifood Research and Development Service (SERIDA), 33394 Deva, Gijón, Spain.

[5]BIOFITER Research Group, Institute of Environmental Sciences (IUCA), University of Zaragoza, 22071 Huesca, Spain.

Table of contents:

Supplementary Table S1: Full list of the 802 identified proteins after the proteomic analysis by SWATH LC/MS-MS, of the sperm samples of high (H) and low (L) fertility bulls.

Supplementary Table S1. Full list of proteins identified in high fertility(H) and low fertility(L) group

N	Accesion	Protein names	Gene Names	Length	Mass	Peptides (95%)	HF (Mean of normalised abundant value)	LF (Mean of normalised abundant value)	FC (HF/LF)	p-value
1	A0A3Q1LNB4	Dynein axonemal heavy chain 10	DNAH10	4573	525861	10	194692,0603	192846,6674	0,99052148	0,83840432
2	A0A3Q1LPJ9	ADAM metallopeptidase domain 20		730	82420	16	120174,5158	157435,2421	1,31005514	0,21532793
3	A0A3Q1LQR2	Tubulin alpha chain	LOC112443216	449	49845	68	151797,6075	96154,01664	0,63343565	0,03939215
4	A0A3Q1LUM9	methylcrotonoyl-CoA carboxylase (EC 6.4.1.4) (3-methylcrotonyl-CoA ca	MCCC2	562	61254	2	25850,14032	22740,28254	0,87969668	0,72945253
5	A0A3Q1LYJ4	Histone H2A	H2AL3	117	13368	9	105581,5496	127251,3469	1,20524227	0,34725896
6	A0A3Q1M0U9	Transitional endoplasmic reticulum ATPase (EC 3.6.4.6) (15S Mg(2+)-AT	VCP	796	88783	12	355080,94	337719,9432	0,95110693	0,84787307
7	A0A3Q1M2V9	Transmembrane protein 11	TMEM11	202	22684	3	513884,9341	554165,0259	1,07838348	0,73364007
8	A0A3Q1M7D1	Dynein axonemal heavy chain 12	DNAH12	3962	454302	12	105841,0148	140610,9674	1,32851114	0,2508943
9	A0A3Q1MA90	Transmembrane protein 190	TMEM190	258	28311	11	14443578,12	14149079,69	0,97961042	0,89500547
10	A0A3Q1MGG8	IQ motif containing N		3540	363545	187	8956856,327	9578028,251	1,06935156	0,5886157
11	A0A3Q1MNG4	BRCA2 and CDKN1A-interacting protein	BCCIP	306	35193	1	190927,7607	230892,9014	1,20932074	0,78235026
12	A0A3Q1MNP8	Dynein light chain roadblock-type 2	DYNLRB2	174	19545	2	295375,9517	244554,6704	0,82794374	0,24833699
13	A0A3Q1MNS9	Theg spermatid protein like	THEGL	450	50856	2	113408,577	125166,1097	1,10367411	0,52375927
14	A0A3Q1N631	Angel homolog 2	ANGEL2	522	59738	1	14202,38251	23302,85447	1,6407708	0,85231388
15	A0A3Q1ND36	Disintegrin and metalloproteinase domain-containing protein 1a-like		822	91364	1	23197,96986	36902,61354	1,5907691	0,05138509
16	A0A3S5ZPN7	Coiled-coil domain containing 116	CCDC116	669	74149	2	112539,1753	116065,2175	1,03133169	0,93646602
17	A0A452DI02	palmitoyl-protein hydrolase (EC 3.1.2.22)	LYPLA1	219	23575	3	379220,5772	324505,1606	0,85571612	0,3953731
18	A0A4W2BMT5	Protein interacting with cyclin A1	PROCA1	376	42242	4	20545,37462	35540,75549	1,72986651	0,03970366
19	A0A4W2BMW7	IQ domain-containing protein F5	LOC113880958 I(	149	18051	2	1844593,614	1447274,813	0,78460361	0,09316436
20	A0A4W2BP90	Jagunal homolog 1	JAGN1	183	21102	2	28301,38327	32254,29972	1,1396722	0,76019132
21	A0A4W2BQ23	Lysozyme like 4	LYZL4	145	16293	11	1019948,095	285717,4277	0,28012938	0,22799159
22	A0A4W2BRF2	Mitochondrial import inner membrane translocase subunit Tim21		244	27962	1	29293,69356	35472,77158	1,21093544	0,44504667
23	A0A4W2BT41	Epididymal-specific lipocalin-5-like	LOC113901868	180	19569	2	45345,22378	43830,44882	0,96659461	0,73833285
24	A0A4W2BT96	ATP synthase subunit alpha		588	63130	91	150891,8748	159547,6884	1,05736435	0,94927689
25	A0A4W2BTQ2	Kinesin-like protein		854	97297	2	38097,45857	41641,25112	1,09301913	0,61050377
26	A0A4W2BU14	Glycerophosphodiester phosphodiesterase 1	GDE1	331	37653	3	363375,3975	320038,174	0,88073705	0,38080066
27	A0A4W2BU38	Ubiquinol-cytochrome c reductase, Rieske iron-sulfur polypeptide 1		464	50631	9	1202846,588	1381509,514	1,14853343	0,30236773
28	A0A4W2BUW4	VAMP associated protein A	VAPA	294	32454	14	459024,4646	395318,0706	0,86121351	0,22652698
29	A0A4W2BV22	Septin 4	SEPTIN4	477	54875	3	107652,6233	120045,6686	1,1151207	0,47326006
30	A0A4W2BVV1	Adenylate kinase 8 (EC 2.7.4.6)	AK8	498	56728	10	782458,9436	711474,6323	0,90928047	0,46917062
31	A0A4W2BVW5	Proteasome assembly chaperone 2	PSMG2	264	29224	2	27544,48319	30801,28824	1,11823802	0,55985748
32	A0A4W2BWR0	Chromosome 26 C10orf82 homolog	C26H10orf82	283	31820	9	1015345,614	974138,9647	0,95941614	0,69854527
33	A0A4W2BWY0	Voltage dependent anion channel 3	VDAC3	283	30739	64	36874664,88	33722329,72	0,91451217	0,49103722
34	A0A4W2BZ57	carnitine O-palmitoyltransferase (EC 2.3.1.21)	CPT1B	771	88512	21	764237,0068	649673,1115	0,85009376	0,38312569
35	A0A4W2BZV4	Radial spoke head component 9	RSPH9	276	31294	12	1203752,476	971998,4725	0,80747371	0,26548157
36	A0A4W2C0V1	Cytochrome c1, heme protein, mitochondrial	LOC113903907 C	325	35297	21	1879109,804	1888357,045	1,00492108	0,92878547
37	A0A4W2C0W3	EF-hand domain containing 1	EFHC1	640	74004	23	1157569,748	1217221,778	1,05153213	0,64553496
38	A0A4W2C225	WBP2 N-terminal like		313	31966	16	2249351,318	1918807,638	0,85304933	0,73240424
39	A0A4W2C4M9	Aspartate aminotransferase (EC 2.6.1.1)	GOT1	413	46399	4	23393,0254	24000,39604	1,02596375	0,48412093
40	A0A4W2C4X4	Dynein axonemal heavy chain 17	DNAH17	4462	509199	81	15468,50381	36928,41333	2,38732936	0,15840353
41	A0A4W2C5D2	CDGSH iron sulfur domain 1	CISD1	106	11983	3	30799,33291	35750,125	1,16074348	0,33959932
42	A0A4W2C6J1	Leucine zipper protein 2	LUZP2	340	38973	55	2870837,307	4290346,187	1,49445814	0,05525953
43	A0A4W2C6Q1	Cytochrome c oxidase subunit (Cytochrome c oxidase polypeptide VIa)	GATC	126	14105	3	63861,8827	70115,46107	1,09792349	0,55068599

44	A0A4W2C6R9	Adenine phosphoribosyltransferase (EC 2.4.2.7)		246	26442	2	10872,15088	7964,500094	0,73255975	0,17110253
45	A0A4W2C6V1	Rh family C glycoprotein		598	65278	2	81549,85363	57083,44221	0,69998215	0,60034449
46	A0A4W2C855	Patched domain-containing protein 3-like	LOC113906256	933	103629	5	43993,0197	35827,12673	0,81438208	0,38827327
47	A0A4W2C8I6	Phosphatidylethanolamine binding protein 1	PEBP1	187	20986	10	604980,6978	743666,9958	1,22924086	0,23590128
48	A0A4W2CCN9	Apolipoprotein A1		207	23868	1	22206,56291	6178,013932	0,27820667	0,05825752
49	A0A4W2CDD2	Glucose-6-phosphate isomerase (EC 5.3.1.9)	GPI	565	63636	14	558379,181	536164,6279	0,96021601	0,77756988
50	A0A4W2CDK1	ATP synthase subunit gamma	ATP5F1C	298	33072	29	2836975,745	2900464,217	1,02237893	0,79997007
51	A0A4W2CE44	Heat shock protein family B (small) member 9	HSPB9	157	16784	15	2209747,385	3052646,599	1,38144596	0,01892325
52	A0A4W2CEF3	Glyceraldehyde-3-phosphate dehydrogenase (EC 1.2.1.12)	GAPDHS	403	44104	141	30449684,32	30853859,08	1,01327353	0,89711401
53	A0A4W2CF85	Calpain small subunit 1	CAPNS1	263	27931	1	45701,93566	31494,88635	0,68913681	0,33806485
54	A0A4W2CG68	Leucine-rich repeat-containing protein 51	LRRC51	192	22107	3	83886,53294	69266,21633	0,82571319	0,29301438
55	A0A4W2CHI3	Cytochrome c oxidase subunit	LOC113875989 C	88	10533	10	2748560,14	3023183,176	1,09991524	0,40159222
56	A0A4W2CHJ7	Septin 10		487	56824	4	92594,4814	92276,40933	0,99656489	0,51940354
57	A0A4W2CIX0	Fibronectin type-II domain-containing protein		183	21292	16	3515759,178	2762301,823	0,78569142	0,48027816
58	A0A4W2CJE1	Chromosome 1 open reading frame 158		196	23234	3	36177,17122	42920,78163	1,18640513	0,25706278
59	A0A4W2CKE1	Protein FAM24A		97	10404	3	72195,38548	116359,6055	1,6117319	0,07177695
60	A0A4W2CMS0	Luteinizing hormone subunit beta	LHB	338	37441	2	19973,26764	26302,6919	1,31689478	0,94875182
61	A0A4W2CN20	Beta-galactosidase		1073	120740	105	451399,7022	521211,0824	1,15465535	0,34718578
62	A0A4W2CP64	Destrin, actin depolymerizing factor	DSTN	165	18506	1	81444,82901	69478,22753	0,85307107	0,30449383
63	A0A4W2CPA4	Regulator of G protein signaling 22	RGS22	1254	145932	4	20358,03066	10231,29626	0,50256807	0,0127063
64	A0A4W2CPR2	ATP-dependent RNA helicase (EC 3.6.4.13)	EIF4A1	406	46154	2	54834,99772	37482,21221	0,68354543	0,11342291
65	A0A4W2CQX1	ATP-dependent (S)-NAD(P)H-hydrate dehydratase (EC 4.2.1.93) (ATP-de	NAXD CARKD	345	36103	2	72060,17544	95015,83611	1,31856238	0,26141578
66	A0A4W2CR59	Proteasome subunit beta	PSMB6	239	25542	5	275791,0295	293435,4339	1,06397744	0,68444659
67	A0A4W2CS05	Calmodulin binding transcription activator 1	VAMP3	148	15514	2	108658,2341	74728,71835	0,68774096	0,02070081
68	A0A4W2CSR1	Cytochrome c oxidase subunit 6C (Cytochrome c oxidase polypeptide V	LOC113904380	74	8610	6	3945733,482	4867329,714	1,23356779	0,19341668
69	A0A4W2CTQ1	Aspartate aminotransferase (EC 2.6.1.1)	GOT2	454	50363	14	1309778,824	1532269,902	1,1698692	0,33385086
70	A0A4W2CUA3	GTP:AMP phosphotransferase AK3, mitochondrial (EC 2.7.4.10) (Adenyl	AK3	227	25671	6	403509,9898	368017,4509	0,91204049	0,66376366
71	A0A4W2CUP5	Ubiquitin C		1146	128851	10	708039,4799	749645,3084	1,05876202	0,84508719
72	A0A4W2CVV1	Mannose-6-phosphate isomerase (EC 5.3.1.8)	MPI	423	46369	4	107602,9481	74567,67416	0,69298914	0,10632406
73	A0A4W2CWK5	ATP synthase F1 subunit epsilon	ATP5F1E	51	5783	3	318044,1468	347229,5673	1,09176531	0,71954839
74	A0A4W2CXU5	Casein kinase 2 alpha 1	CSNK2A1	391	45144	11	317792,6939	291587,0362	0,91753851	0,48483908
75	A0A4W2CYV7	NADH dehydrogenase [ubiquinone] 1 alpha subcomplex subunit 7 (Com	NDUFA7	113	12677	2	31632,92403	32053,0567	1,0132815	0,88276605
76	A0A4W2CZ27	Coiled-coil-helix-coiled-coil-helix domain containing 3	CHCHD3	232	26685	2	7415,163966	7045,449834	0,9501408	0,69747708
77	A0A4W2CZJ1	LY6/PLAUR domain containing 4	LYPD4	246	26824	3	23959,90937	27341,9631	1,1411547	0,88600106
78	A0A4W2D0C7	Protein kinase cAMP-dependent type I regulatory subunit alpha	PRKAR1A	380	42893	11	1153274,879	1052575,461	0,91268394	0,46984422
79	A0A4W2D0I6	Sperm acrosome associated 1	SPACA1	297	32850	26	9485702,249	11826240,23	1,24674377	0,1972812
80	A0A4W2D0I8	H1.7 linker histone		265	29159	4	180320,7268	164660,7121	0,91315466	0,55390463
81	A0A4W2D126	Superoxide dismutase [Mn], mitochondrial (EC 1.15.1.1)		556	59294	13	428865,3838	469579,0688	1,09493348	0,56854577
82	A0A4W2D1Z5	Uncharacterized protein		5914	656236	54	685039,4432	843610,8704	1,23147781	0,07873497
83	A0A4W2D277	Clusterin	CLU	439	51114	21	1668596,459	1498460,419	0,89803644	0,93437932
84	A0A4W2D2C2	NADH-ubiquinone oxidoreductase 75 kDa subunit, mitochondrial		875	96701	23	2426379,88	2355671,163	0,97085835	0,75996641
85	A0A4W2D2D8	Transmembrane protein 38B	TMEM38B	291	32451	12	1253101,137	892235,6861	0,71202209	0,08219288
86	A0A4W2D3D4	Calmodulin 1	CALM1	365	38625	17	160814,7587	157588,1584	0,97993592	0,68147063
87	A0A4W2D4I1	Calcium/calmodulin dependent protein kinase IV	CAMK4	463	49902	3	80204,50585	64151,8111	0,79985296	0,21584482
88	A0A4W2D4S5	Lon protease homolog, mitochondrial (EC 3.4.21.53) (Lon protease-like	LONP1	1027	113918	19	895786,0842	987579,2088	1,10247215	0,47712212
89	A0A4W2D592	Fibrous sheath interacting protein 2		6744	759927	129	25860,32397	23262,17803	0,89953158	0,38939496
90	A0A4W2D5V1	NADH dehydrogenase [ubiquinone] 1 alpha subcomplex subunit 12		220	25453	5	91825,03154	79967,13496	0,87086423	0,36897882
91	A0A4W2D819	Tyrosine 3-monooxygenase/tryptophan 5-monooxygenase activation p	YWHAZ	229	26085	12	567080,9906	476432,5636	0,84014906	0,17321

92	A0A4W2D8B4	SPATA31/FAM205 domain-containing protein		1254	138951	9	16944,36268	14743,3807	0,87010535	0,63068593
93	A0A4W2D8G0	Sperm equatorial segment protein 1	SPESP1	345	38997	39	3295309,693	3665402,734	1,11230903	0,37083503
94	A0A4W2D8T3	Albumin	ALB	655	74084	202	107680,1795	107071,301	0,99434549	0,78465834
95	A0A4W2D9A8	SPATA31 domain-containing protein		1523	173961	23	613352,8116	627870,7659	1,02366983	0,83647606
96	A0A4W2D9T4	Testis expressed 46	TEX46	162	18816	1	29856,53323	27965,14948	0,93665093	0,87545885
97	A0A4W2DBJ7	dTMP kinase (EC 2.7.4.9)	DTYMK	228	25491	1	39513,53977	22033,93264	0,55762994	0,3956885
98	A0A4W2DDE0	Ring finger protein 130	RNF130	462	50538	1	22721,82022	18401,56201	0,80986302	0,86691145
99	A0A4W2DER0	High mobility group box 4	HMGB4	247	28855	4	88810,30471	68621,2677	0,77267236	0,47640913
100	A0A4W2DFR9	Ferritin		157	17860	13	2947661,038	1807247,907	0,61311253	0,0293047
101	A0A4W2DHN1	Medium-chain specific acyl-CoA dehydrogenase, mitochondrial (EC 1.3.	ACADM	421	46573	31	1744468,02	1798520,454	1,03098505	0,76533629
102	A0A4W2DIS9	Keratin 75		561	60110	9	219149,2508	497049,7945	2,26808804	0,10508249
103	A0A4W2DLZ3	Actin-like protein 7A	ACTL7A	438	49016	43	2912881,789	2576964,631	0,88467875	0,58560457
104	A0A4W2DMG8	Heat shock protein 90 beta family member 1		808	92862	3	479689,7459	293152,6127	0,61112962	0,05812982
105	A0A4W2DNN5	Isoaspartyl peptidase/L-asparaginase (EC 3.4.19.5) (EC 3.5.1.1) (Asparaş	ASRGL1	308	32050	27	5161743,766	4912512,61	0,95171571	0,84594272
106	A0A4W2DNW3	Calcium binding tyrosine phosphorylation regulated		382	41488	26	123951,4564	119347,0899	0,96285347	0,65808051
107	A0A4W2DQ38	IQ motif containing F1	IQCF1	209	24194	14	179465,2844	305592,9067	1,70279677	0,01094425
108	A0A4W2DQI3	Betaine--homocysteine S-methyltransferase	BHMT	538	59271	2	43664,26539	53220,29284	1,21885236	0,0883168
109	A0A4W2DRC1	Spermatid-specific manchette-related protein 1		166	19396	15	215810,0954	246110,9497	1,14040518	0,3668477
110	A0A4W2DS83	Nucleoside diphosphate kinase (EC 2.7.4.6)	LOC113883266 D	187	20457	3	21433,92549	23376,01531	1,09060822	0,57315398
111	A0A4W2DSH2	NADH dehydrogenase [ubiquinone] 1 alpha subcomplex subunit 10, mi	NDUFA10	343	39278	11	795760,0369	750085,6821	0,94260285	0,66064907
112	A0A4W2DSL6	Arylsulfatase A	ARSA	507	53807	8	220749,4335	317620,085	1,43882627	0,56855863
113	A0A4W2DTA2	Solute carrier family 25 member 22	SLC25A22	322	34531	3	82207,37531	79992,14995	0,9730532	0,93031206
114	A0A4W2DTM9	Phosphoglycerate kinase (EC 2.7.2.3)		417	44781	40	2724992,298	3352040,982	1,23011026	0,10411154
115	A0A4W2DTV6	S-formylglutathione hydrolase (EC 3.1.2.12)	ESD	282	31548	1	70555,36033	70609,40594	1,000766	0,79236596
116	A0A4W2DTX9	Secretoglobin family 1D member		123	13632	2	18066,07296	19471,26491	1,07778071	0,7859356
117	A0A4W2DVI7	NADPH:adrenodoxin oxidoreductase, mitochondrial (EC 1.18.1.6)		463	50858	2	78882,32541	58119,76302	0,73679069	0,19711189
118	A0A4W2DW14	Multifunctional fusion protein [Includes: Delta-1-pyrroline-5-carboxylat	ALDH4A1	563	61495	10	538002,3306	592436,8853	1,10117903	0,42045459
119	A0A4W2DY45	Protein kinase cAMP-dependent type II regulatory subunit alpha	PRKAR2A	401	45094	21	2172425,847	2425729,122	1,11659927	0,43526268
120	A0A4W2DYA7	Triosephosphate isomerase (EC 5.3.1.1)	TPI1	286	30577	55	12430003,37	13249309,53	1,06591359	0,58195526
121	A0A4W2DYH1	Isocitrate dehydrogenase [NADP] (EC 1.1.1.42)	IDH2	477	53617	17	961735,3932	947857,3149	0,98556975	0,88732908
122	A0A4W2DYQ2	Actin beta	ACTB	375	41737	23	1401964,33	1313284,949	0,93674634	0,76418434
123	A0A4W2DZI5	EF-hand calcium binding domain 6	EFCAB6	1497	172742	41	2001979,445	2024115,548	1,01105711	0,94182945
124	A0A4W2E067	Serine/threonine-protein phosphatase (EC 3.1.3.16)	PPP1CC	337	38548	35	341034,076	403449,6923	1,18301871	0,34076398
125	A0A4W2E1I7	T-complex 11		473	52661	3	89097,48533	79391,52504	0,89106359	0,49430019
126	A0A4W2E5S0	folate gamma-glutamyl hydrolase (EC 3.4.19.9)	GGH	318	35683	2	2046,407687	1829,497966	0,89400464	0,60384569
127	A0A4W2E5V3	Family with sequence similarity 71 member A		585	63860	20	880889,7026	839692,4313	0,9532322	0,67856612
128	A0A4W2E851	Coiled-coil-helix-coiled-coil-helix domain containing 6	CHCHD6	337	37062	2	43237,56941	32683,29394	0,75590035	0,40445542
129	A0A4W2E8C9	Peroxiredoxin like 2B	PRXL2B	210	23526	1	17043,29008	20076,9275	1,177996	0,72981612
130	A0A4W2E975	Cilia and flagella associated protein 20	CFAP20	193	22748	13	1638721,232	1578344,677	0,9631563	0,71579975
131	A0A4W2E9B9	Tubulin beta chain	TUBB4B	445	49831	199	1063312,211	2123557,557	1,99711574	0,13788829
132	A0A4W2E9V4	ADAM metallopeptidase domain 20		744	83691	6	169933,3537	223456,1129	1,31496324	0,09848086
133	A0A4W2EA76	IF rod domain-containing protein		428	47264	7	23013,94629	11909,07432	0,51747207	0,38681503
134	A0A4W2ECX5	Fibronectin type-II domain-containing protein		356	41036	13	2691094,185	2193876,318	0,8152358	0,62791827
135	A0A4W2EDB5	NADH dehydrogenase [ubiquinone] iron-sulfur protein 6, mitochondria	NDUFS6	124	13413	6	18153,83553	19799,02072	1,09062466	0,55297331
136	A0A4W2EE11	Beta-defensin		101	11774	2	16927,61734	22757,20231	1,34438308	0,38457554
137	A0A4W2EEF6	Aconitate hydratase, mitochondrial (Aconitase) (EC 4.2.1.3)	ACO2	780	85359	61	5086372,569	5166379,911	1,01572974	0,80732622
138	A0A4W2EF91	BPTI/Kunitz inhibitor domain-containing protein		138	15824	3	616117,8388	412751,6146	0,66992317	0,01961867
139	A0A4W2EHT0	T-complex protein 1 subunit beta (CCT-beta)	CCT2	526	56405	10	225078,9551	143580,2316	0,63791051	0,04155914

140	A0A4W2EL77	ATP synthase subunit beta (EC 7.1.2.2)	ATP5F1B	528	56284	105	55095462,73	50386248,51	0,91452628	0,4335244
141	A0A4W2ELI9	Tripeptidyl-peptidase 2 (EC 3.4.14.10)		1297	143824	3	26464,65857	66090,96132	2,49732908	0,09333829
142	A0A4W2ELT9	NADH dehydrogenase [ubiquinone] 1 alpha subcomplex subunit 8 (Con	NDUFA8	278	31103	7	645715,9811	556909,3368	0,86246795	0,24233814
143	A0A4W2EMT1	Coiled-coil domain containing 65	CCDC65	498	58814	2	20833,0864	15478,85571	0,74299388	0,58679009
144	A0A4W2EMY6	Potassium voltage-gated channel interacting protein 3	LOC113900915	341	37632	5	415025,8355	341305,8335	0,8223725	0,1673304
145	A0A4W2ENE8	Serine/threonine-protein phosphatase (EC 3.1.3.16)	PPP1CB	445	49621	29	32404,04268	24242,07396	0,74811881	0,2306312
146	A0A4W2EPP2	glutamate dehydrogenase [NAD(P)(+)] (EC 1.4.1.3)	GLUD1	561	61631	2	56068,0457	57632,48944	1,02790259	0,98554619
147	A0A4W2EPS9	Acyl-CoA synthetase bubblegum family member 2		678	75715	9	346153,9456	418989,3799	1,21041342	0,50568335
148	A0A4W2ERA1	Actin like 7B	ACTL7B	417	45563	14	973171,639	976415,3644	1,00333315	0,88415819
149	A0A4W2ERC1	Acyl-CoA thioesterase 4	ACOT4	452	50576	6	350056,6619	383694,1836	1,09609165	0,58112233
150	A0A4W2ERW4	Multifunctional fusion protein [Includes: Poly [ADP-ribose] polymerase	PARP6	926	102497	42	905230,0236	928239,431	1,0254183	0,59042702
151	A0A4W2ERZ2	ATP synthase subunit b	ATP5PB	256	28806	17	1074464,479	1338911,975	1,24612028	0,19021066
152	A0A4W2EU79	Proteasome subunit alpha type	PSMA3	266	29875	7	154059,3994	129915,6216	0,84328267	0,40544368
153	A0A4W2EUH8	Cytochrome c 2	LOC113901903	105	11740	8	964526,9754	1134967,697	1,17670913	0,68998524
154	A0A4W2EV06	ATP synthase F1 subunit delta	ATP5F1D	168	17612	12	571994,6992	917214,9921	1,60353757	0,08183834
155	A0A4W2EVB5	Chromosome 1 open reading frame 56		350	37917	46	19966792,89	24586003,68	1,23134465	0,04799039
156	A0A4W2EX92	F-actin-capping protein subunit beta	CAPZB	301	33741	62	2910306,859	3074473,947	1,05640886	0,68595929
157	A0A4W2EY09	Glycerol-3-phosphate dehydrogenase (EC 1.1.5.3)	GPD2	727	80797	42	4145116,164	5001681,623	1,2066445	0,12601957
158	A0A4W2EYG8	Cytochrome b-c1 complex subunit 6	LOC113889946	91	10624	4	236600,7741	240077,9501	1,01469638	0,99186713
159	A0A4W2EYI0	ATP synthase subunit O, mitochondrial (ATP synthase peripheral stalk s	ATP5PO	213	23320	24	1332180,838	1420472,231	1,06627583	0,68644076
160	A0A4W2EZ36	Superoxide dismutase [Cu-Zn] (EC 1.15.1.1)		330	35056	4	368270,4625	364247,3927	0,98907577	0,74714358
161	A0A4W2F0A5	NAD(P)H-hydrate epimerase (EC 5.1.99.6) (Apolipoprotein A-I-binding p	APOA1BP AIBP	363	38374	3	102506,5105	92319,60857	0,9006219	0,72125757
162	A0A4W2F1D4	cAMP-dependent protein kinase (EC 2.7.11.11)	PRKACA	343	39889	14	955000,3999	1015698,421	1,06355811	0,60965439
163	A0A4W2F2N0	NADH dehydrogenase [ubiquinone] flavoprotein 3, mitochondrial (Com	NDUFV3	447	48351	5	37546,76102	56369,01758	1,50130174	0,1162922
164	A0A4W2F3T3	Phosphate carrier protein, mitochondrial (Phosphate transport protein	SLC25A3	361	40004	10	742950,6901	660083,3054	0,8884618	0,40180063
165	A0A4W2F4K2	ATP synthase subunit d, mitochondrial	ATP5PD	161	18692	14	2555947,801	2394774,471	0,93694185	0,55350207
166	A0A4W2F4Y9	Cytochrome c oxidase subunit 7C, mitochondrial (Cytochrome c oxidase	LOC113900450	63	7331	1	9011,594736	26051,64061	2,89090237	0,00119925
167	A0A4W2F5L1	Galactokinase 1	GALK1	392	42265	3	274385,8967	186349,7294	0,679152	0,53662976
168	A0A4W2F767	Transmembrane and coiled-coil domains 2	TMCO2	179	19952	3	3639,979652	7164,39474	1,96825132	0,02015952
169	A0A4W2F7C1	NADH dehydrogenase [ubiquinone] 1 beta subcomplex subunit 11, mito	NDUFB11	154	17573	3	61699,09432	71001,78569	1,15077517	0,39022065
170	A0A4W2F836	Sodium/potassium-transporting ATPase subunit beta	ATP1B3	279	31521	3	370720,9352	315751,7202	0,85172347	0,34645819
171	A0A4W2F8A2	NADH dehydrogenase [ubiquinone] 1 alpha subcomplex subunit 6 (Con	NDUFA6	154	17799	1	22157,51937	22638,99025	1,02172946	0,94611234
172	A0A4W2F904	Dynein axonemal intermediate chain 7	DNAI7	721	83769	2	83164,64705	55338,3622	0,66540729	0,08711828
173	A0A4W2F919	GLIPR1 like 1	GLIPR1L1	241	27110	12	233500,7676	279036,0722	1,19501137	0,23624216
174	A0A4W2F9T8	RIB43A domain with coiled-coils 2	RIBC2	381	45169	6	193430,1333	165181,6032	0,85396003	0,4307402
175	A0A4W2F9Z4	Adenylate kinase 9	AK9	1902	220085	4	132181,4797	145803,144	1,10305274	0,6901099
176	A0A4W2FA84	Prohibitin	PHB1 PHB	272	29804	18	3602988,315	3043722,383	0,8447772	0,17101177
177	A0A4W2FA94	Complex III subunit 9	UQCR10 LOC113	64	7458	3	870137,2257	1099280,715	1,26334178	0,16343577
178	A0A4W2FB35	Dynein axonemal heavy chain 1	DNAH1	4266	487581	14	287008,3931	300197,2863	1,04595299	0,80927883
179	A0A4W2FBC3	Phosphoinositide phospholipase C (EC 3.1.4.11)	PLCZ1	637	74100	19	2566315,915	3191061,987	1,24344083	0,31707487
180	A0A4W2FCM2	NADH dehydrogenase [ubiquinone] iron-sulfur protein 7, mitochondria	NDUFS7	216	23771	4	114435,7864	140839,0011	1,23072516	0,14297185
181	A0A4W2FCS9	10 kDa heat shock protein, mitochondrial (10 kDa chaperonin) (Chapero	LOC113905824	259	27646	10	943685,8054	1092801,682	1,15801433	0,2719659
182	A0A4W2FD88	FAM161 centrosomal protein A	FAM161A	721	84200	8	214700,1183	210773,1643	0,98170959	0,85738229
183	A0A4W2FDF0	T-complex protein 1 subunit delta	CCT4	545	58389	14	613325,3306	448035,8476	0,73050276	0,07094405
184	A0A4W2FDX9	T-complex protein 1 subunit eta (TCP-1-eta) (CCT-eta)	CCT7	543	59443	10	115821,6171	80734,29043	0,69705719	0,10728168
185	A0A4W2FE87	Malate dehydrogenase (EC 1.1.1.37)	MDH1	245	27009	2	306991,5539	350134,9246	1,14053602	0,29494411
186	A0A4W2FED5	Cytochrome b-c1 complex subunit 8 (Complex III subunit 8) (Complex II	UQCRQ LOC1138	82	9720	3	296098,0434	249461,5183	0,84249634	0,41183708
187	A0A4W2FEJ5	Cytochrome c oxidase subunit 4	COX4I1 LOC1138	169	19572	14	6846768,458	8852662,958	1,29296952	0,1762314

188	A0A4W2FEV1	IQ motif containing F2	IQCF2	163	19536	1	198823,9331	192429,319	0,9678378	0,92968875
189	A0A4W2FF31	Mitochondria-eating protein (Spermatogenesis-associated protein 18)	SPATA18	557	62902	23	4289766,601	3770239,477	0,87889152	0,45422246
190	A0A4W2FF64	Armadillo like helical domain containing 1	ARMH1	442	49408	3	32706,47221	31073,24523	0,9500641	0,64233604
191	A0A4W2FFW8	Reactive oxygen species modulator 1 (Protein MGR2 homolog)	ROMO1	79	8183	2	57842,58072	62127,75637	1,07408341	0,49167987
192	A0A4W2FG39	OCIA domain-containing protein 1	OCIAD1	220	24540	3	194371,0619	162031,6151	0,83362005	0,32882975
193	A0A4W2FG68	Malate dehydrogenase (EC 1.1.1.37)	MDH2	338	35668	41	14308937,48	14836756,3	1,03688735	0,72791121
194	A0A4W2FGL7	Family with sequence similarity 162 member A	FAM162A	156	17737	1	66015,27691	29061,82179	0,44022873	0,02448074
195	A0A4W2FHP1	Cytochrome c oxidase subunit 5A, mitochondrial (Cytochrome c oxidase	LOC113879686	152	16735	20	1268441,01	1430761,601	1,12796858	0,60268089
196	A0A4W2FHT1	Cytochrome b-c1 complex subunit 7 (Complex III subunit VII) (Ubiquino	LOC113904403	160	19070	9	520142,8082	534998,6884	1,02856116	0,78473647
197	A0A4W2FIJ7	Protein FAM205A-like	LOC113897149	1287	142386	8	476657,531	470493,952	0,98706917	0,98128023
198	A0A4W2FIR4	SPATA31/FAM205 domain-containing protein		1388	153579	28	3315032,659	3732156,584	1,125828	0,31332659
199	A0A4W2FJA4	ADP/ATP translocase (ADP,ATP carrier protein)	SLC25A31	323	35754	41	85281,94665	50486,13603	0,5919909	0,09803355
200	A0A4W2FJN7	NADH dehydrogenase [ubiquinone] 1 beta subcomplex subunit 8, mitoc	NDUFB8	186	21684	4	37485,17768	21189,21515	0,56526917	0,00794545
201	A0A4W2FL75	Cilia and flagella associated protein 77	CFAP77	284	32907	7	47782,72672	48529,61173	1,01563086	0,85759486
202	A0A4W2FNN4	NADH dehydrogenase [ubiquinone] 1 alpha subcomplex subunit 11 (Co	NDUFA11	141	14788	2	459038,6235	412404,7994	0,8984098	0,44635672
203	A0A4W2FPB2	Hydroxysteroid 17-beta dehydrogenase 10	HSD17B10	288	30151	13	820337,0242	735609,0393	0,89671564	0,3518797
204	A0A4W2FPY7	Ubiquitin carboxyl-terminal hydrolase (EC 3.4.19.12)	UCHL1	339	37946	3	12235,37977	12009,08002	0,98150448	0,95989733
205	A0A4W2FQC5	Glutathione peroxidase	GPX5	219	25049	10	214021,7194	98145,0973	0,45857541	0,08051892
206	A0A4W2FQT7	Proteasome subunit alpha type	PSMA4	261	29484	5	202765,4712	140761,6587	0,69420922	0,03997485
207	A0A4W2FQT8	Electron transfer flavoprotein-ubiquinone oxidoreductase (ETF-QO) (EC	ETFDH	617	68612	10	234296,8113	263532,7939	1,12478182	0,44217414
208	A0A4W2FRD0	Thioredoxin domain containing 8	TXNDC8	127	14647	4	1422375,577	1608083,18	1,13056158	0,32005709
209	A0A4W2FS86	Peroxiredoxin-5 (EC 1.11.1.24)	PRDX5	219	23253	19	3553904,432	3283446,544	0,92389838	0,63375742
210	A0A4W2FTM0	Diazepam-binding inhibitor-like 5	LOC113877332	87	9788	12	60779,81213	138370,4251	2,27658527	0,01197623
211	A0A4W2FUK5	Adenylate kinase 2, mitochondrial (AK 2) (EC 2.7.4.3) (ATP-AMP transpl	AK2	308	33534	2	40073,6759	39519,30595	0,98616623	0,8502221
212	A0A4W2FUX7	Serine/threonine-protein phosphatase 2A activator (EC 5.2.1.8) (Phospl	PTPA	295	33617	1	8996,218138	7418,30962	0,82460313	0,54394233
213	A0A4W2FV49	Voltage dependent anion channel 2	VDAC2	294	31620	62	38105809,78	41124414,01	1,07921638	0,77661225
214	A0A4W2FVY1	ATP synthase subunit f, mitochondrial (ATP synthase membrane subun	ATP5MF	88	10297	8	1229993,527	1291977,211	1,0503935	0,61019715
215	A0A4W2FY09	ADP/ATP translocase (ADP,ATP carrier protein)	SLC25A4	298	32967	16	286598,5578	275180,311	0,96015944	0,65671342
216	A0A4W2FY37	AU RNA binding methylglutaconyl-CoA hydratase	AUH	339	36204	5	834934,9091	860169,7011	1,03022366	0,79730225
217	A0A4W2FY99	Cilia and flagella associated protein 70	CFAP70	1124	126320	4	53591,75832	55358,95195	1,0329751	0,78017105
218	A0A4W2FYF3	Cilia and flagella associated protein 44	CFAP44	1852	213456	6	46191,32755	61246,80914	1,32593741	0,30268171
219	A0A4W2FZN9	C-type natriuretic peptide	NPPC	193	20294	7	77622,1764	148098,7024	1,90794318	0,05661821
220	A0A4W2G035	EF-hand domain containing 2	EFHC2	747	87007	17	1559102,305	1375319,807	0,88212287	0,25376422
221	A0A4W2G038	Chromosome 4 C7orf31 homolog	C7orf31	583	67483	14	176187,5906	172185,0843	0,9772827	0,69630941
222	A0A4W2G0J9	Chromosome 8 C9orf135 homolog	CFAP95 C8H9orf	218	25092	2	197358,9081	219150,5424	1,11041627	0,55556509
223	A0A4W2G0N7	Heat shock protein 90 alpha family class A member 1	HSP90AA1	776	89100	5	576278,3658	312118,3454	0,54161038	0,00337358
224	A0A4W2G182	Tektin	TEKT3	490	56700	45	754590,0423	801511,1362	1,06218091	0,80542501
225	A0A4W2G186	Cytochrome c	LOC113891379	105	11704	4	83037,82735	117199,5899	1,4114	0,0123413
226	A0A4W2G1A4	Keratin, type II cytoskeletal 2 oral-like	KRT76	597	62847	2	136214,8924	478659,9625	3,5140061	0,04057013
227	A0A4W2G1U8	Abhydrolase domain containing 10	ABHD10	330	36403	9	597637,4872	703645,6393	1,17737869	0,35608126
228	A0A4W2G4G1	2-iminobutanoate/2-iminopropanoate deaminase (EC 3.5.99.10) (Trans		137	14517	3	168492,7634	173488,7086	1,0296508	0,70966283
229	A0A4W2G5C6	Dynein axonemal heavy chain 3	DNAH3	4069	466147	13	196652,7625	212960,9335	1,08292877	0,81205736
230	A0A4W2G5W8	Dynein regulatory complex subunit 7	DRC7	807	95871	2	36160,71544	28960,83109	0,80089209	0,2429258
231	A0A4W2G637	Solute carrier family 16 member 1	SLC16A1 LOC113	501	54297	6	24649,22636	27094,86486	1,09921766	0,79116163
232	A0A4W2G685	Chromosome 10 C15orf65 homolog	PIERCE2 C10H15	120	13771	1	18950,36487	15870,85653	0,83749609	0,75703385
233	A0A4W2G6F8	thioredoxin-disulfide reductase (EC 1.8.1.9)	TXNRD2	488	52514	3	26424,04326	26847,04066	1,01600805	0,80567596
234	A0A4W2G6V9	NADH dehydrogenase [ubiquinone] 1 beta subcomplex subunit 5, mitoc	NDUFB5	189	21589	1	20701,72427	14269,95991	0,68931263	0,37728664
235	A0A4W2G9X2	Cancer/testis antigen 47A-like	LOC113888212	281	30261	4	120557,7269	122546,3654	1,01649532	0,96551352

236	A0A4W2GAI0	Cytochrome b-c1 complex subunit 2, mitochondrial	LOC113883210	453	48149	57	4485094,827	4524615,842	1,00881163	0,9049328
237	A0A4W2GC72	NADH dehydrogenase [ubiquinone] 1 beta subcomplex subunit 7 (Com	NDUFB7	137	16398	2	112417,4191	130064,6817	1,15697979	0,37102903
238	A0A4W2GCD2	NADH dehydrogenase [ubiquinone] iron-sulfur protein 2, mitochondria	NDUFS2	510	57393	4	199985,7476	118104,9516	0,59056684	0,0864451
239	A0A4W2GCX1	Coiled-coil domain containing 113	CCDC113	323	37773	3	66789,86242	82070,64813	1,228789	0,28573142
240	A0A4W2GDN1	Acetyl-CoA acetyltransferase 1	ACAT1	422	44889	19	927723,6351	806597,6988	0,86943748	0,4451806
241	A0A4W2GDV6	NADH-cytochrome b5 reductase (EC 1.6.2.2)	CYB5R1	304	33723	17	5151143,304	4189221,709	0,81326056	0,36238864
242	A0A4W2GE75	Tubulin polymerization promoting protein family member 2	TPPP2	171	18564	9	171172,7159	392984,282	2,29583482	0,00057773
243	A0A4W2GES6	aldehyde dehydrogenase (NAD(+)) (EC 1.2.1.3)	ALDH7A1	539	58596	2	66058,99358	61748,9526	0,93475467	0,8797945
244	A0A4W2GEV4	Glyceraldehyde-3-phosphate dehydrogenase (EC 1.2.1.12)	GAPDH	333	35868	18	5237458,376	7330088,135	1,39955062	0,58412367
245	A0A4W2GFX5	Milk fat globule-EGF factor 8 protein	MFGE8	572	63527	16	2303661,254	2083488,963	0,90442506	0,39961818
246	A0A4W2GG79	Fibronectin type-II domain-containing protein		151	17304	53	42818711,73	22999374,18	0,53713373	0,21776875
247	A0A4W2GGE1	Proteasome subunit alpha type	PSMA2	234	25899	4	113918,9787	118542,9307	1,04058983	0,72780991
248	A0A4W2GGZ0	NADH dehydrogenase [ubiquinone] flavoprotein 1, mitochondrial (EC 7	NDUFV1	464	50652	15	805154,7645	714788,7784	0,88776569	0,38674356
249	A0A4W2GHS6	Proteasome 26S subunit, non-ATPase 2	PSMD2 LOC1138	1688	188624	1	3224,700484	2654,966982	0,82332204	0,59202576
250	A0A4W2GIZ5	A-kinase anchoring protein 3	AKAP3	810	89355	134	1497013,05	1259442,392	0,84130355	0,32128415
251	A0A4W2GJ13	14-3-3 protein theta	YWHAQ	421	47052	2	23521,76904	10198,52725	0,43357824	0,00601817
252	A0A4W2GL58	Family with sequence similarity 24 member A		89	9388	2	2596434,044	2053930,874	0,79105837	0,34316798
253	A0A4W2GL67	Membrane cofactor protein		335	37318	3	37736,95551	36014,61985	0,95435944	0,6647011
254	A0A4W2GLB6	NADH dehydrogenase [ubiquinone] 1 alpha subcomplex subunit 5 (Con	NDUFA5	116	13316	10	230965,0303	194124,46	0,84049286	0,34465906
255	A0A4W2GMB7	Cilia and flagella associated protein 65	CFAP65	1740	196959	2	32191,84334	28888,13367	0,89737432	0,68206242
256	A0A4W2GMQ8	STI1 domain-containing protein		238	26971	1	28107,99437	22880,61102	0,81402503	0,63460243
257	A0A4W2GMV9	Cytochrome c oxidase subunit 7A-related protein, mitochondrial	COX7A2L LOC11	114	12544	2	32784,71713	42733,76562	1,30346605	0,02705586
258	A0A4W2GNK5	Transmembrane protein 256	TMEM256	113	11775	1	12394,17221	14828,39973	1,19640098	0,60384636
259	A0A4W2GPB3	SPEM family member 2	SPEM2	497	56428	1	13343,85138	17455,65782	1,3081424	0,19544292
260	A0A4W2GQJ6	Nucleoside diphosphate kinase 7 (EC 2.7.4.6)	NME7	377	42599	16	888640,5963	801750,9709	0,90222186	0,38253668
261	A0A4W2GQM1	Leucine rich repeat containing 37A	LRRC37A	2532	278113	72	5216374,377	6663611,774	1,27744124	0,11549595
262	A0A4W2GQY8	NADH dehydrogenase [ubiquinone] 1 alpha subcomplex subunit 2		92	10600	4	402773,2735	461634,1185	1,1461389	0,27913988
263	A0A4W2GRQ0	MICOS complex subunit	APOO	210	24002	5	203703,0505	143861,5588	0,70623174	0,13604902
264	A0A4W2GRR1	Fascin	FSCN3	498	56194	28	1596760,98	1529478,052	0,95786287	0,65834651
265	A0A4W2GSC1	Cytochrome c oxidase subunit 5B, mitochondrial (Cytochrome c oxidase	LOC113894051	129	13834	12	1795561,714	2043682,743	1,13818574	0,88680043
266	A0A4W2GUI0	L-lactate dehydrogenase (EC 1.1.1.27)	LDHA	332	36598	29	9630995,258	10614861,74	1,10215626	0,9775027
267	A0A4W2GVQ9	Sperm associated antigen 16	SPAG16	628	70485	1	10481,99887	29133,4203	2,7793764	0,00079217
268	A0A4W2GW60	Histone H2A/H2B/H3 domain-containing protein		136	15678	2	61558,24147	38379,69869	0,62346971	0,11037522
269	A0A4W2GWM3	Dihydrolipoyllysine-residue succinyltransferase component of 2-oxoglu	DLST	529	56572	20	706956,9036	734743,0191	1,03930383	0,79520435
270	A0A4W2GWX1	Polyamine modulated factor 1 binding protein 1	PMFBP1	1006	117813	3	8288,711392	5805,802859	0,70044698	0,35921855
271	A0A4W2GWX2	Dynein regulatory complex protein 10 (IQ domain-containing protein D	IQCD	444	51570	2	27090,18183	23543,65456	0,86908441	0,31676689
272	A0A4W2GXD6	Proteasome subunit alpha type	PSMA6	203	22842	2	8587,083442	9031,759137	1,05178425	0,68310932
273	A0A4W2GXF1	Glucose-6-phosphatase (EC 3.1.3.9)	G6PC3	346	38742	2	18366,74988	18182,23606	0,98995392	0,9445032
274	A0A4W2GXI2	Crystallin zeta	CRYZ	330	35382	3	163735,4311	140239,4773	0,85650049	0,45245146
275	A0A4W2GXN7	Sperm acrosome associated 4	SPACA4	126	12964	3	298922,2207	388515,4528	1,29972088	0,33266935
276	A0A4W2GYF8	Peptidase, mitochondrial processing beta subunit	PMPCB	491	54266	2	57390,91435	71000,23856	1,23713378	0,21256515
277	A0A4W2GZC3	Propionyl-CoA carboxylase subunit beta	PCCB	544	58644	1	30894,8019	29214,52936	0,9456131	0,34908831
278	A0A4W2GZU2	Acetyl-CoA acyltransferase 2	ACAA2	645	68438	19	556476,8512	549454,0504	0,98737989	0,98268466
279	A0A4W2H0C9	LanC like 1	LANCL1	399	45309	6	622975,2453	571857,2598	0,9179454	0,53274226
280	A0A4W2H0D3	Aldehyde dehydrogenase 2 family member	ALDH2	520	56653	19	1408174,771	1579930,923	1,12197076	0,3821682
281	A0A4W2H1Z3	Aldose reductase	LOC113891189	303	34500	1	28022,97979	27704,83711	0,98864708	0,78121162
282	A0A4W2H1Z5	T-complex protein 1 subunit theta (CCT-theta)	CCT8	553	60203	6	344372,8723	216916,1881	0,62988756	0,1137795
283	A0A4W2H2D6	Proteasome subunit beta	PSMB4	264	29031	1	7531,310721	3939,30598	0,52305716	0,01736808

284	A0A4W2H537	Tektin	TEKT2	430	49886	36	7587333,156	7637977,889	1,00667491	0,95064382
285	A0A4W2H5E7	Cytochrome c oxidase subunit 7A2		83	9283	6	2597632,53	2916622,386	1,12280022	0,48864967
286	A0A4W2H5R5	NADH dehydrogenase [ubiquinone] 1 beta subcomplex subunit 9 (Com	NDUFB9	179	21789	4	114442,5576	121534,8947	1,0619729	0,59044109
287	A0A4W2H658	Pyruvate dehydrogenase E1 component subunit beta (EC 1.2.4.1)	PDHB	359	39098	40	4165973,084	4891160,587	1,17407398	0,46037027
288	A0A4W2H6M4	Actin like 10	ACTL10	383	41133	3	86744,44624	83371,60856	0,96111754	0,8684529
289	A0A4W2H7K9	Family with sequence similarity 166 member C	FAM166C	201	23321	2	39388,5512	37731,98071	0,95794284	0,53923673
290	A0A4W2H888	Methionine adenosyltransferase 1A	DYDC1	175	20730	1	26911,49864	30168,8353	1,12103884	0,84134278
291	A0A4W2H8Q1	SAMM50 sorting and assembly machinery component	SAMM50	469	52042	8	262299,9369	264832,6139	1,00965565	0,9942525
292	A0A4W2H983	NAD(+) kinase (EC 2.7.1.23)	NADK2	555	61200	3	76409,73079	75489,57722	0,98795764	0,76460693
293	A0A4W2HAB9	Spermatogenesis associated 19	SPATA19	154	17899	8	2186560,914	2345861,26	1,07285429	0,61651972
294	A0A4W2HBQ2	Alpha-L-fucosidase (EC 3.2.1.51)	FUCA1	444	51405	1	32783,03786	38992,81969	1,18942057	0,8135249
295	A0A4W2HCD2	Cilia and flagella associated protein 57	CFAP57	1279	148428	6	139923,8068	136551,1272	0,97589631	0,73172348
296	A0A4W2HCZ3	Fibrous sheath interacting protein 2	FSIP2	6725	757955	128	146693,5421	175135,6768	1,19388812	0,58840789
297	A0A4W2HD14	Outer dense fiber of sperm tails 3B	ODF3B	395	42380	2	78338,42145	80640,35706	1,0293845	0,85764438
298	A0A4W2HD58	ATP-binding cassette sub-family A member 17-like	LOC113883545	1334	150599	2	15899,46687	12352,53105	0,7769148	0,75802595
299	A0A4W2HDC1	Calicin	CCIN	588	66890	64	47284611,14	44091518,81	0,93247079	0,48562773
300	A0A4W2HDX7	Cytochrome b-c1 complex subunit 10	UQCR11 LOC113	56	6520	2	37756,41904	49346,84427	1,30697893	0,24628605
301	A0A4W2HF26	Glycerol-3-phosphate dehydrogenase [NAD(+)] (EC 1.1.1.8)		351	38301	3	59093,27908	66691,43251	1,12857898	0,66852744
302	A0A4W2HFN3	Cullin associated and neddylation dissociated 1	CAND1	1223	135587	3	93743,90548	64057,6712	0,68332625	0,11084248
303	A0A4W2HG11	AFG3 like matrix AAA peptidase subunit 2	AFG3L2	805	89388	11	838441,3903	1119186,113	1,3348412	0,08500163
304	A0A4W2HG44	Histidine triad nucleotide binding protein 2	HINT2	165	17317	6	195313,8458	258661,4139	1,32433731	0,08776537
305	A0A4W2HGQ2	Sperm acrosome-associated protein 5	LOC113886686	156	17580	3	70929,81799	116407,699	1,64116732	0,48040314
306	A0A4W2HH02	IZUMO family member 2	IZUMO2	221	25293	1	41509,70195	61005,10437	1,46965894	0,65115601
307	A0A4W2HH43	Electron transfer flavoprotein subunit alpha (Alpha-ETF)	ETFA	311	32704	16	17117,46513	10974,48456	0,64112791	0,44407548
308	A0A4W2HHB8	proton-translocating NAD(P)(+) transhydrogenase (EC 7.1.1.1)	NNT	1034	108491	8	530261,8471	502503,6745	0,94765195	0,67964036
309	A0A4W2HI57	Tektin	TEKT1	418	48719	16	480959,647	566878,8506	1,17864119	0,14422094
310	A0A4W2HIR1	HtrA serine peptidase 2	HTRA2	361	38786	4	162106,5422	184863,7919	1,14038452	0,56547423
311	A0A4W2HIT4	Dynein axonemal heavy chain 6	DNAH6	4154	475638	14	235762,762	213455,3143	0,9053818	0,41612594
312	A0A4W2HIX9	Hydroxysteroid dehydrogenase like 2	HSDL2	418	45135	10	1278947,271	1231166,054	0,9626402	0,78461962
313	A0A4W2HJ36	Heat shock protein family A (Hsp70) member 4 like	HSPA4L	1023	115519	5	78455,61579	65642,04682	0,83667748	0,3543384
314	A0A4W2HJ53	NADH dehydrogenase [ubiquinone] 1 alpha subcomplex subunit 13	GATAD2A	174	19597	2	484659,579	457007,2235	0,94294479	0,58421126
315	A0A4W2HJC0	Testis expressed 51		168	18788	1	11347,62736	18647,74492	1,64331664	0,19849697
316	A0A4W2HK76	Carboxypeptidase Q (Plasma glutamate carboxypeptidase)	CPQ	484	53024	12	11897248,1	15062631,07	1,2660601	0,13476126
317	A0A4W2HKM7	NADH dehydrogenase [ubiquinone] 1 beta subcomplex subunit 4 (Com	NDUFB4	129	15184	4	495286,1538	462773,9412	0,93435671	0,58466611
318	A0A4W2HKS8	Actin like 9	ACTL9	416	45540	23	1783217,02	2182245,904	1,22376911	0,10255887
319	A0A4W2HL85	Myeloid leukemia factor 1	MLF1	270	31227	7	108781,3667	125979,0793	1,15809429	0,31826418
320	A0A4W2HLP1	FUN14 domain containing 2	FUNDC2	190	20577	6	316658,0152	363109,6452	1,14669337	0,43688663
321	A0A4W2HLQ2	Tubulin beta chain	TUBB3	512	56029	104	89672,98977	182911,0686	2,03975655	0,04932613
322	A0A4W2HM97	L-serine ammonia-lyase (EC 4.3.1.17)	SRR	339	36681	4	39595,25647	35763,19111	0,90321908	0,47559661
323	A0A4W2HME8	Spermatogenesis associated 48	SPATA48	440	49710	3	69875,61459	83559,66826	1,19583447	0,61600254
324	A0A4W2HN15	Sorbitol dehydrogenase (SDH) (XDH) (EC 1.1.1.14) (EC 1.1.1.9) (L-iditol 2	SORD	356	38127	3	134761,7294	116264,4562	0,8627409	0,32583258
325	A0A4W2HP47	Proteasome subunit alpha type	PSMA5	241	26411	4	212554,0154	225547,4667	1,06113011	0,63729274
326	A0A4W2HPH7	ADP/ATP translocase (ADP,ATP carrier protein)	SLC25A6	410	44462	20	1542812,619	1428463,709	0,92588283	0,56792582
327	A0A4W2HPP0	Histone H2A		238	26496	8	444589,7754	341802,3891	0,76880398	0,31950158
328	A0A4W2HQP5	Nucleoredoxin (EC 1.8.1.8)	GLOD4	594	65077	1	23266,71825	19586,93315	0,8418434	0,29930618
329	A0A4W2HQW9	Stress-70 protein, mitochondrial (75 kDa glucose-regulated protein) (H	HSPA9	679	73772	19	1931228,684	1874522,799	0,97063741	0,80028109
330	A0A4W2HRF0	Citrate synthase	CS	466	51773	41	10278665,6	10228335,76	0,99510347	0,86501319
331	A0A4W2HRM3	Leucine-rich repeat and IQ domain-containing protein 4-like	LOC113888572	565	64870	1	27653,71627	16502,35209	0,59674989	0,14109104

332	A0A4W2HRY1	EF-hand calcium binding domain 5	EFCAB5	1505	172053	4	24082,62548	18765,14713	0,77919856	0,55904665
333	A0A4W2HS31	3-hydroxyisobutyrate dehydrogenase, mitochondrial (EC 1.1.1.31)	HIBADH	336	35410	20	938652,6041	1101883,561	1,17389922	0,18099919
334	A0A4W2HSU6	T-complex protein 1 subunit gamma	CCT3	507	56483	12	1388038,356	1007204,727	0,72563177	0,09407357
335	A0A4W2HSV6	Rab GDP dissociation inhibitor	GDI1	447	50566	2	96771,51962	91234,5605	0,94278317	0,81002075
336	A0A4W2HT18	Actin related protein T2	ACTRT2	377	41913	56	11343764,94	12147537,31	1,07085587	0,6139985
337	A0A4W2HTE9	H(+)-transporting two-sector ATPase (EC 7.1.2.2)	ATP6V1A	618	68475	2	51112,49326	42760,683	0,83659944	0,28502442
338	A0A4W2HTI2	Histone H2B subacrosomal variant	LOC113879192	122	14238	20	29251487,54	26267647,6	0,89799357	0,52577928
339	A0A4W2HU94	Serpin A3-1 (Serpin family A member 5)	SERPINA5 LOC11	374	41701	2	68286,91725	63771,90259	0,9338817	0,57453664
340	A0A4W2HVH6	Outer dense fiber of sperm tails 2	ODF2	701	80750	294	889093,0831	766609,1222	0,86223719	0,3571311
341	A0A4W2HVJ6	Electron transfer flavoprotein subunit beta	ETFB	357	38331	6	622745,3218	587254,944	0,9430098	0,66314443
342	A0A4W2HVK1	pyruvate carboxylase (EC 6.4.1.1)	PC	1266	139249	3	23473,7703	24521,12874	1,04461825	0,71265616
343	A0A4W2HVV4	Chromosome 5 C12orf10 homolog	MYG1	381	42967	7	282695,1247	311150,6895	1,10065814	0,48924749
344	A0A4W2HW44	Cytosol aminopeptidase (EC 3.4.11.1) (EC 3.4.11.5) (EC 3.4.13.23) (Cyst	LAP3	519	56289	24	1331944,65	1347888,986	1,01197072	0,92745757
345	A0A4W2HWC1	Dihydrolipoamide acetyltransferase component of pyruvate dehydroge	PDHX	501	53886	13	535456,5238	630121,4483	1,17679292	0,31414196
346	A0A4W2HWK9	Isocitrate dehydrogenase [NAD] subunit, mitochondrial	IDH3B	385	42497	4	163111,7133	197887,4184	1,21320177	0,16051024
347	A0A4W2HWP1	Renin receptor (ATPase H(+)-transporting lysosomal accessory protein	ATP6AP2	377	42449	1	44566,17039	29936,68168	0,67173557	0,27652982
348	A0A4W2HWQ4	Coiled-coil domain containing 81	CCDC81	565	66189	3	90086,4404	75172,39108	0,83444735	0,23365973
349	A0A4W2HXB2	Seminal plasma protein PDC-109	LOC113876423	134	15580	25	13496380,86	8083728,625	0,59895528	0,23746183
350	A0A4W2HXI7	Protein SCO1 homolog, mitochondrial	SCO1	305	33717	2	34589,24056	31475,87337	0,9099903	0,99503274
351	A0A4W2HXJ5	Adenylate kinase isoenzyme 1 (AK 1) (EC 2.7.4.3) (EC 2.7.4.6) (ATP-AMF	AK1	211	23442	21	11936892,78	13543453,37	1,13458784	0,27482379
352	A0A4W2HXU7	Radial spoke head 3	RSPH3	606	68696	3	188067,6659	167561,5406	0,89096411	0,56021574
353	A0A4W2HY76	Chaperonin containing TCP1 subunit 6B	CCT6B	518	56605	8	23453,99399	12497,65132	0,53285813	0,05883556
354	A0A4W2HY90	DnaJ heat shock protein family (Hsp40) member C11	DNAJC11	559	63237	2	48852,11253	29667,03308	0,6072825	0,10496673
355	A0A4W2HYH2	protein deglycase (EC 3.5.1.124) (Maillard deglycase) (Parkinsonism-ass	PARK7	189	20035	10	468845,1469	551101,0754	1,1754437	0,26529007
356	A0A4W2HYH3	Dynein light chain Tctex-type 1	DYNLT1	113	12452	3	30535,38881	24412,08602	0,79946865	0,25518546
357	A0A4W2HYZ9	Mitochondrial carrier 2	MTCH2	303	33280	49	25605879,77	24516929,06	0,95747263	0,76924753
358	A0A4W2HZD8	Meiosis-specific nuclear structural protein 1	MNS1	526	64310	3	31310,99931	28339,84985	0,90510844	0,424992
359	A0A4W2HZR1	Mitochondrial glutathione transporter SLC25A40 (Solute carrier family	LOC113891614	339	38277	2	119808,5889	116069,6464	0,96879237	0,92908176
360	A0A4W2HZT3	Testis expressed 49	TEX49	131	15447	1	73881,55817	61023,9253	0,82596966	0,19972847
361	A0A4W2I018	Solute carrier family 2, facilitated glucose transporter member 5 (Fruct	SLC2A5	508	56009	1	612257,6983	569695,9952	0,930484	0,60635758
362	A0A4W2I0K8	Prohibitin	PHB2	299	33357	14	1047179,325	981020,5499	0,93682192	0,67407358
363	A0A4W2I1H5	Histidine triad nucleotide binding protein 1	HINT1	126	13779	1	12036,80117	15119,88085	1,25613779	0,34408882
364	A0A4W2I1K4	Coiled-coil domain containing 63	CCDC63	558	65966	7	108288,3821	89174,63144	0,82349214	0,38636409
365	A0A4W2I1X5	Succinate dehydrogenase cytochrome b560 subunit, mitochondrial	SDHC	135	14561	1	182419,2296	269970,0584	1,47994298	0,12283922
366	A0A4W2I292	hexokinase (EC 2.7.1.1)	HK1	921	102497	63	16578554,08	15291533,27	0,92236833	0,46276144
367	A0A4W2I2Q2	Heat shock 70 kDa protein 1-like	HSPA1L LOC1138	641	70363	34	2747360,852	2054025,851	0,74763599	0,08848345
368	A0A4W2I344	Cytochrome b-c1 complex subunit 1, mitochondrial	UQCRC1	490	53827	37	5795285,023	6850980,405	1,18216453	0,37435474
369	A0A4W2I3C7	Hyaluronidase (EC 3.2.1.35)	LOC113891262	554	62449	46	26756,24338	90056,3633	3,36580745	0,24314133
370	A0A4W2I4L1	Aspartyl aminopeptidase	DNPEP	433	47465	6	362676,3355	294904,9845	0,81313545	0,53358398
371	A0A4W2I5J8	ATP-dependent 6-phosphofructokinase (ATP-PFK) (Phosphofructokinas	PFKM	850	93427	5	206367,3277	246597,0667	1,19494238	0,21239082
372	A0A4W2I5U3	Apolipoprotein A-II (Apolipoprotein A2)	APOA2	100	11202	2	4701,18315	6399,069795	1,36116156	0,22986715
373	A0A4W2I6G9	Sperm acrosome associated 3	SPACA3	163	18099	14	10767314,32	13597619,81	1,26286086	0,14965881
374	A0A4W2I6S5	Peptidase S1 domain-containing protein		350	38411	7	727835,2424	534141,1247	0,73387642	0,19115701
375	A0A4W2I8C2	Dynein axonemal intermediate chain 1	DNAI1	734	83124	4	96337,36552	108124,5499	1,12235319	0,39550897
376	A0A4W2I8F3	NADH dehydrogenase [ubiquinone] iron-sulfur protein 8, mitochondria	NDUFS8	195	22231	3	426853,4352	412359,1219	0,96604382	0,95461429
377	A0A4W2I8P6	Acyl-CoA dehydrogenase very long chain	ACADVL	677	72731	39	4133288,368	4598815,102	1,11262866	0,45418626
378	A0A4W2I8R7	Dihydroxyacetone phosphate acyltransferase (DAP-AT) (DHAP-AT) (EC 2	GNPAT	680	77682	11	163063,44	146258,7661	0,89694395	0,39391665
379	A0A4W2I8V6	Mitochondrial chaperone BCS1 (BCS1-like protein)	BCS1L LOC11387	419	47504	4	185847,1107	163104,0417	0,87762484	0,27337111

380	A0A4W2I9C4	Enoyl-CoA hydratase, short chain 1	ECHS1	290	31178	5	115460,318	92581,4781	0,80184673	0,16480327
381	A0A4W2I9M3	Ribonucleic acid export 1	RAE1	368	40981	1	19402,71019	18947,96372	0,97656273	0,87020363
382	A0A4W2I9W7	RAB2A, member RAS oncogene family	RAB2A	212	23519	47	8151631,785	7842040,824	0,96202098	0,658865
383	A0A4W2IB22	Leucine rich repeat containing 74A	LRRC74A	486	54176	2	178013,3562	213658,123	1,20023647	0,15895247
384	A0A4W2IB54	Fructose-bisphosphate aldolase (EC 4.1.2.13)	ALDOA	445	48167	79	3990255,299	4501548,768	1,12813553	0,70500691
385	A0A4W2ICU0	Four and a half LIM domains 1	FHL1	309	35024	6	27564,95733	46484,27623	1,68635401	0,23424915
386	A0A4W2ICW9	Profilin	PFN3	137	14629	14	3274036,161	2975354,303	0,90877258	0,49619781
387	A0A4W2IDE6	UBC core domain-containing protein		147	16673	1	38717,41573	17746,46893	0,45835882	0,03864029
388	A0A4W2IEM6	Dynein light chain	DNAL4	134	14634	2	281297,8258	297627,0311	1,05804953	0,40209482
389	A0A4W2IEV4	Transmembrane protein 14C	TMEM14C	114	11721	2	99354,4328	96887,9022	0,97517443	0,87965741
390	A0A4W2IFA5	Succinate dehydrogenase [ubiquinone] iron-sulfur subunit, mitochondr	SDHB	285	31993	8	2203263,148	2056821,299	0,93353411	0,78733223
391	A0A4W2II32	Tudor domain-containing protein 3	TDRD3	736	82237	1	2531108,733	2592115,598	1,02410282	0,80519105
392	A0A4W2II81	Transmembrane protein 65	TMEM65	236	25041	1	146884,1174	167012,8665	1,1370383	0,40772514
393	A0A4W2IIC9	T-complex-associated-testis-expressed 1	TCTE1	502	55376	1	34237,69415	22481,93079	0,65664267	0,08758891
394	A0A4W2IJI3	Disintegrin and metalloproteinase domain-containing protein 20-like	LOC113897899	723	82091	22	363648,0547	453400,263	1,24681064	0,58178222
395	A0A4W2IK05	Coiled-coil domain containing 114	CCDC114	797	89300	3	44899,24747	56767,38634	1,26432824	0,20068854
396	A0A4W2IKF1	Glutamine synthetase (EC 6.3.1.2)	GLUL	373	42031	26	5076089,387	4959929,928	0,97711635	0,76481645
397	A0A4W2IKL9	3-hydroxybutyrate dehydrogenase 1	BDH1	344	38391	7	660835,3082	608275,5519	0,92046467	0,50993416
398	A0A4W2IKP9	tRNA-dihydrouridine synthase (EC 1.3.1.-)	BCAP29	369	42809	2	31609,23263	26493,95525	0,83817142	0,80223886
399	A0A4W2IL98	Synaptophysin like 1	SYPL1	297	32290	3	624421,2493	413842,3749	0,66276152	0,1329668
400	A0A4W2ILV6	Pyruvate kinase (EC 2.7.1.40)	PKM	655	71874	42	26334,08836	34981,48679	1,3283728	0,23716097
401	A0A4W2IMA0	Succinate--CoA ligase [GDP-forming] subunit beta, mitochondrial (EC 6.	SUCLG2	432	46663	39	458700,5621	476385,4179	1,03855425	0,72287096
402	A0A4W2IMG1	Keratin associated protein 6-2	KRTAP6-2	69	7021	1	1550985,912	6616,764033	0,00426617	0,41775683
403	A0A4W2IPA3	BPI fold containing family A member 1	BPIFA1	306	32258	1	44095,43205	62815,75062	1,42454099	0,63550486
404	A0A4W2IQG1	Keratin 24	KRT24	525	55115	5	107833,4785	68631,82071	0,63646116	0,49545004
405	A0A4W2IQJ0	Family with sequence similarity 81 member B	FAM81B	445	51475	5	111488,6788	83195,93257	0,74622763	0,3941872
406	A0A4W2IR05	Outer dynein arm-docking complex subunit 4 (Tetratricopeptide repeat	ODAD4 TTC25	683	78354	10	367767,8303	153815,2267	0,41824003	0,00920154
407	A0A4W2IRM1	Cilia- and flagella-associated protein 206	CFAP206	582	66645	3	14672,98852	21130,58547	1,440101	0,26373714
408	A0A4W2IRR0	Malate dehydrogenase 1B	MDH1B	526	59163	1	17099,59018	22352,83877	1,30721488	0,54682693
409	A0A4W2ITX2	NADH dehydrogenase [ubiquinone] 1 beta subcomplex subunit 3 (Com	NDUFB3	179	20284	1	124369,7025	147377,1575	1,18499244	0,42263116
410	A0JN77	Triokinase/FMN cyclase (EC 4.6.1.15) (Bifunctional ATP-dependent dihy	TKFC DAK	578	59160	1	28727,91614	20014,12725	0,6966787	0,1093267
411	A1A4J9	DnaJ (Hsp40) homolog, subfamily A, member 3 (DnaJ heat shock protei	DNAJA3	453	49254	6	200080,9584	225498,4099	1,12703583	0,3182514
412	A1A4K9	Succinyl-CoA:3-ketoacid-coenzyme A transferase (EC 2.8.3.5)	OXCT2	517	56009	3	18981,5385	40992,48912	2,15959782	0,49949545
413	A1A4P8	Family with sequence similarity 209, member A (LOC784495 protein)	LOC784495 FAM	168	19359	9	448081,3792	602449,3836	1,34450886	0,05352008
414	A2VDL2	Solute carrier family 2, facilitated glucose transporter member 3 (GLUT	SLC2A3	494	54119	15	4466920,943	4989288,887	1,11694139	0,49009688
415	A2VDZ0	Serine/threonine-protein phosphatase 2A 56 kDa regulatory subunit	PPP2R5A	489	56526	1	19632,33029	25139,6083	1,28052085	0,20961978
416	A4FV36	Spermatogenesis-associated protein 20	SPATA20	789	88360	5	154345,1501	153477,6302	0,99437935	0,87857147
417	A4IFP2	KRT4 protein (Keratin 4)	KRT4	549	58046	12	252563,8614	105156,4967	0,41635607	0,23588848
418	A5D7S5	SLC25A35 protein (Solute carrier family 25 member 35)	SLC25A35	311	33688	2	56304,93656	55637,23037	0,98814125	0,95865144
419	A5D954	Solute carrier family 25 (Mitochondrial carrier; oxoglutarate carrier), m	SLC25A11	311	33899	3	225766,8954	205768,625	0,91142071	0,55546989
420	A5D973	Alpha isoform of regulatory subunit A, protein phosphatase 2 (Protein	PPP2R1A	589	65282	1	28083,27198	15851,12879	0,56443312	0,49614471
421	A5D9E7	Mitochondrial trifunctional protein, beta subunit	HADHB	496	53704	17	2776889,516	2566960,845	0,9244015	0,56066612
422	A5D9G3	Succinate-CoA ligase subunit beta (EC 6.2.1.-)	LOC283398	432	46664	38	70925,70138	62734,64122	0,8845121	0,31038666
423	A5PJ81	LACTB protein (Lactamase beta)	LACTB	556	61653	3	93290,95823	83349,46559	0,89343563	0,43530025
424	A5PJA6	Stomatin (EPB72)-like 2 (Stomatin like 2)	STOML2	356	38733	2	18502,30425	28316,22639	1,53041621	0,80920325
425	A5PJD4	KLKBL4 protein (Serine protease 54)	PRSS54 KLKBL4	327	36921	3	322757,6035	356240,6623	1,10374057	0,59211666
426	A5PK71	Parkin coregulated gene protein	PACRG	257	29284	15	2535602,457	2212393,82	0,87253182	0,22448333
427	A5PKG4	cysteine desulfurase (EC 2.8.1.7)	NFS1	457	50344	3	34739,15368	32139,54287	0,9251677	0,8914297

428	A5PKH9	POC1 centriolar protein B (WDR51B protein)	POC1B WDR51B	478	53723	5	46278,73783	40148,47608	0,86753611	0,42660974
429	A6H746	C12H13ORF26 protein	C12H13ORF26	290	33737	1	113997,4481	119527,1736	1,04850745	0,88029867
430	A6H758	C11H9ORF9 protein	C11H9ORF9	223	25006	23	3862369,947	3848334,391	0,99636608	0,83410104
431	A6H782	Tektin-3	TEKT3 OMC45	490	56681	45	9665,114645	26588,08245	2,75093296	0,56544628
432	A6QLB8	[tau protein] kinase (EC 2.7.11.26)	GSK3A	495	52263	6	59368,99908	51741,02711	0,87151591	0,84634585
433	A6QLG3	Protein-serine/threonine kinase (EC 2.7.11.-)	PDK3	415	47913	1	132536,7613	124030,5567	0,93582004	0,6378087
434	A6QLL2	MTX2 protein (Metaxin 2)	MTX2	264	29679	3	148140,3698	176186,0317	1,18931816	0,18234541
435	A6QNM9	SLC25A12 protein (Solute carrier family 25 member 12)	SLC25A12	675	74530	8	270930,3771	183648,8255	0,67784509	0,06319588
436	A6QNX2	DPP7 protein (Dipeptidyl peptidase 7)	DPP7	488	53642	6	532552,2874	709168,5015	1,33164108	0,08822087
437	A6QNZ7	Keratin 10 (Keratin 10 (Epidermolytic hyperkeratosis; keratosis palmari	KRT10	526	54849	13	343857,2742	553006,6917	1,60824485	0,2589725
438	A6QPG6	LANCL2 protein (LanC like 2)	LANCL2	433	49166	6	226580,9259	245644,2418	1,08413469	0,51059983
439	A6QQ54	Phosphate carrier protein, mitochondrial (Phosphate transport protein	LOC616319	330	36606	13	2482869,956	2217021,103	0,89292679	0,56434098
440	A6QQK5	MMAA protein	MMAA	421	46687	1	27818,66923	26964,57414	0,96929777	0,72449954
441	A7E3P5	15-oxoprostaglandin 13-reductase (EC 1.3.1.48) (15-oxoprostaglandin 1	ZADH1	296	32091	2	62253,00139	56850,48794	0,91321682	0,48379974
442	A7E3Q2	Heat shock 70kDa protein 1A (Heat shock protein family A (Hsp70) mer	HSPA2	636	69809	22	1719488,311	1342163,892	0,78056006	0,11538186
443	A7MB90	non-specific serine/threonine protein kinase (EC 2.7.11.1)	CSNK1G2	414	47268	3	94120,48965	114894,4855	1,22071704	0,47948802
444	A7MBF3	STYXL1 protein (Serine/threonine/tyrosine interacting like 1)	STYXL1	309	35367	1	30568,59115	29999,09353	0,98136984	0,76844445
445	A7Z057	Tyrosine 3-monooxygenase/tryptophan 5-monooxygenase activation p	YWHAG	247	28303	2	19200,08045	56094,96554	2,92160055	0,05755201
446	B2KJ42	fructose-bisphosphatase (EC 3.1.3.11)		338	36740	13	245499,524	204929,6872	0,83474576	0,21942459
447	B5B3R8	Alpha-S1-casein	CSN1S1	214	24443	1	9921,915224	9768,800862	0,98456806	0,58553199
448	C9QNT9	TEPP protein (Testis, prostate and placenta expressed)	TEPP	216	24905	14	1808523,446	1821042,35	1,00692217	0,93181813
449	D5K1R5	Sperm protamine P1	PRM1	51	6713	1	21186,74417	81647,49013	3,85370633	0,03667663
450	E1B6Z7	Metabolism of cobalamin associated B	MMAB	241	26615	4	162288,1834	176994,1498	1,09061637	0,52110762
451	E1B7Q2	Adenylate kinase 7	AK7	724	83016	4	170158,0597	176985,3326	1,04012312	0,706883
452	E1B7S8	Acrosin-binding protein (Acrosin-binding protein, 60 kDa form) (Proacr	ACRBP	542	61273	118	59668232,96	67621903,48	1,13329824	0,33093893
453	E1B7X2	Actin like 11	ACTL11	1204	129465	112	824523,5024	1020570,223	1,23776972	0,28647721
454	E1B836	Enkurin	ENKUR	259	30157	8	131990,2623	126178,1864	0,95596587	0,98951086
455	E1B844	Mitochondrial import inner membrane translocase subunit TIM44	TIMM44	459	52004	2	29158,5881	16300,72265	0,55903676	0,04924022
456	E1B8N5	Sodium/potassium-transporting ATPase subunit alpha	ATP1A4	1030	113734	27	1180079,024	1077068,699	0,91270896	0,33075609
457	E1B8R6	RBR-type E3 ubiquitin transferase (EC 2.3.2.31)	ARIH2	491	57517	3	58175,36632	54693,47384	0,94014834	0,54370639
458	E1B8W3	Outer dynein arm-docking complex subunit 2 (Armadillo repeat-contair	ODAD2 ARMC4	1044	115859	12	217852,778	236725,3659	1,08663001	0,51493002
459	E1B958	ALMS1 centrosome and basal body associated protein	ALMS1	4331	482210	5	120544,8567	180052,7967	1,49365806	0,014907
460	E1B993	Ankyrin repeat and EF-hand domain containing 1	ANKEF1	776	86915	1	38083,76913	31047,98004	0,81525492	0,30860516
461	E1B9F6	Elongation factor 1-alpha		440	48049	3	332831,2605	247811,4126	0,7445557	0,13452446
462	E1B9R5	Dynein axonemal heavy chain 8	DNAH8	4730	541255	74	2138741,401	2109762,644	0,98645056	0,88313812
463	E1B9Y3	Lipocalin 12	LCN12	193	21636	2	48023,68178	41493,63417	0,86402443	0,91669801
464	E1BA21	Transcription factor A, mitochondrial	TFAM	246	28830	7	1379275,037	1027561,662	0,74500128	0,12973855
465	E1BA36	Sperm associated antigen 17	SPAG17	2229	252206	1	72731,99504	61567,99257	0,84650493	0,3214035
466	E1BAJ3	Coiled-coil domain containing 40	CCDC40	1125	129259	4	125886,8799	169323,4082	1,34504412	0,15285281
467	E1BBC4	Dynamin-like 120 kDa protein, mitochondrial (EC 3.6.5.5)	OPA1	1015	117520	2	28121,46999	49159,57184	1,7481153	0,09049913
468	E1BC58	RAB2B, member RAS onco family	RAB2B	216	24167	36	3555425,42	3831453,11	1,07763563	0,54990024
469	E1BCC9	Small nuclear ribonucleoprotein Sm D1 (snRNP core protein D1)	SNRPD1	134	14689	1	14690,43543	7874,474377	0,5360273	0,03793087
470	E1BCN8	Phosphoethanolamine/phosphocholine phosphatase 1	PHOSPHO1	267	29687	5	220456,2193	226305,9656	1,02653473	0,91999922
471	E1BCW3	ATP-dependent 6-phosphofructokinase (ATP-PFK) (Phosphofructokinas	PFKP	960	102968	10	69643,72569	95645,61422	1,37335579	0,42054935
472	E1BD64	Mitochondrial pyruvate carrier	MPC2	127	14282	1	92345,3563	63408,01656	0,68664001	0,16564852
473	E1BD83	Proteasome subunit alpha type	PSMA8	250	27915	6	503997,0409	441261,7363	0,87552446	0,36289911
474	E1BDA8	Izumo sperm-egg fusion 1	IZUMO1	322	36457	12	1832962,796	2183139,785	1,19104424	0,57033372
475	E1BDM7	Zinc finger MYND-type containing 12	ZMYND12	366	41736	3	170335,9915	164335,1376	0,96477049	0,67407747

476	E1BDQ6	NME/NM23 family member 5	NME5	209	23752	8	75335,78494	88005,5813	1,16817766	0,31973709
477	E1BE64	Testis specific 10	TSGA10	892	103566	87	4052489,748	4454761,887	1,09926543	0,4110469
478	E1BED9	Glutathione S-transferase omega (GSTO) (EC 1.20.4.2) (EC 1.8.5.1) (EC 2	GSTO2	244	28261	29	12560734,34	11257874,31	0,89627517	0,45860603
479	E1BEL9	Coenzyme A synthase	COASY	562	61800	1	43158,59616	45689,33913	1,05863821	0,52303384
480	E1BFM2	Serine protease 50	PRSS50	418	45695	3	166000,7291	150339,5263	0,90565582	0,50742204
481	E1BGD1	Translocase of outer mitochondrial membrane 34	TOMM34	309	34743	2	42521,02523	33376,60389	0,78494354	0,30857589
482	E1BHQ2	Selenoprotein V	SELENOV	381	40603	1	13749,72711	9363,758291	0,68101412	0,29002918
483	E1BHV9	Ornithine decarboxylase antizyme 3	OAZ3	117	13967	8	4062620,153	2774636,189	0,68296717	0,09988199
484	E1BHW8	UBX domain protein 11	UBXN11	475	53409	2	65482,87947	76804,60736	1,172896	0,3554212
485	E1BI52	Serine protease 42	PRSS42	337	36709	2	29436,63774	37492,75391	1,27367651	0,08987619
486	E1BIN5	Cullin 3	CUL3	768	88959	3	23511,29408	35687,29311	1,51787873	0,11245016
487	E1BJB1	Tubulin beta chain	TUBB2A	445	49907	153	17109,4613	22800,01288	1,33259677	0,56143795
488	E1BJG2	Radial spoke head 6 homolog A	RSPH6A	704	79769	19	1544604,831	1583668,929	1,02529067	0,78378898
489	E1BJG7	Chromosome 15 C11orf42 homolog	C15H11orf42	333	36540	2	5248,917357	4170,540577	0,79455253	0,60974929
490	E1BJL9	Cilia- and flagella- associated protein 210	CFAP210 CCDC17	547	65300	4	51608,62122	32876,08041	0,63702691	0,01009472
491	E1BJW4	Cilia and flagella associated protein 69	CFAP69	941	106186	1	37773,06709	19191,90555	0,50808438	0,11490161
492	E1BJX2	glutathione transferase (EC 2.5.1.18)	GSTT4	241	28076	3	25274,1854	17223,39518	0,68146193	0,29564858
493	E1BKF9	Cilia- and flagella-associated protein 52	CFAP52	623	68637	6	290931,2519	310632,5079	1,06771791	0,65733678
494	E1BLB4	Dynein axonemal heavy chain 17	DNAH17	4453	508122	81	11101,85208	10787,56953	0,97169098	0,76673857
495	E1BLI3	Ubiquitin-like protease family profile domain-containing protein		221	25106	3	7265,085643	5057,571504	0,69614754	0,15472165
496	E1BM93	Retinol dehydrogenase 11	RDH11	340	37807	9	322945,08	296824,7496	0,91911835	0,47735093
497	E1BMD1	Cilia and flagella associated protein 43	CFAP43	1665	192505	6	110543,1515	101840,5762	0,92127441	0,6031596
498	E1BMQ6	Calcium-transporting ATPase (EC 7.2.2.10)	ATP2A3	999	109321	1	25538,55471	39259,67778	1,53727093	0,67741096
499	E1BN01	Dynein axonemal light intermediate chain 1	DNALI1	258	29672	17	1320709,118	1518416,275	1,14969773	0,3899278
500	E1BNA2	X-prolyl aminopeptidase 3	XPNPEP3	507	57320	6	224499,8966	201355,2775	0,89690588	0,52889471
501	E1BNL3	Zonadhesin	ZAN	2459	269080	98	6805369,921	9474820,175	1,39225645	0,22706703
502	E1BNS6	Sperm-associated antigen 8	SPAG8	484	51542	7	39642,68448	28883,02691	0,72858403	0,16941917
503	E1BNS9	L-lactate dehydrogenase (EC 1.1.1.27)	LDHC	332	36015	63	20891229,76	20307645,49	0,97206559	0,81033816
504	E1BP40	Cilia and flagella associated protein 61	CFAP61	1236	141136	5	16201,32358	16543,09754	1,02109543	0,72459038
505	E1BPM9	Dynein axonemal intermediate chain 2	DNAI2	616	70120	5	645593,9013	616315,1098	0,95464828	0,63018184
506	E1BPT0	Leucine rich repeats and death domain containing 1	LRRD1	863	98513	4	21463,87254	19139,41868	0,89170389	0,52811076
507	E1BPV0	Armadillo repeat containing 3	ARMC3	871	95801	6	245669,5579	283880,0632	1,15553618	0,23024886
508	E1BPY2	Equatorin	EQTN	133	15118	2	9084,024636	32462,25773	3,57355457	0,00016896
509	F1MAU4	Prolylcarboxypeptidase	PRCP	499	56627	5	767105,7344	929748,1491	1,21202086	0,24607456
510	F1MB31	Peptidase S1 domain-containing protein	PRSS46P	303	33731	2	40222,98547	30724,63159	0,76385756	0,23589091
511	F1MBE1	Threonine synthase like 1	THNSL1	748	83738	2	13737,40066	15584,69966	1,13447224	0,76124087
512	F1MBL6	non-specific serine/threonine protein kinase (EC 2.7.11.1)	SMG1	3658	410232	1	1930778,299	2158031,468	1,11770029	0,45342462
513	F1MBQ1	Serine/threonine-protein phosphatase with EF-hands (EC 3.1.3.16)	PPEF1	658	75965	9	590955,5451	568508,4311	0,96201556	0,75420224
514	F1MBS4	Solute carrier family 25 member 10	SLC25A10	287	31521	1	36876,57923	18215,74197	0,49396507	0,02986606
515	F1MBU8	Dpy-19 like 2	DPY19L2	773	89528	29	4594356,154	3895034,976	0,8477869	0,24707581
516	F1MBV2	ribose-phosphate diphosphokinase (EC 2.7.6.1)	PRPS1L1	329	35658	2	154582,0142	93904,76967	0,60747539	0,0658402
517	F1MBZ1	Kelch like family member 10	KLHL10	608	68916	2	72763,88418	55270,67282	0,75958937	0,35220851
518	F1MC11	Keratin 14	KRT14	515	55895	10	19007,77332	60469,83675	3,18132144	0,25136738
519	F1MDK8	ADP/ATP translocase (ADP,ATP carrier protein)	SLC25A31	323	35654	43	1102647,551	1931280,132	1,7514936	0,3702196
520	F1ME97	5-oxoprolinase, ATP-hydrolysing	OPLAH	1210	128826	5	62382,46605	40109,44739	0,64296027	0,02382874
521	F1MEI0	Fibrous sheath CABYR binding protein	FSCB	818	87746	12	45468,98165	59061,33333	1,2989368	0,23646168
522	F1MEM9	Tetratricopeptide repeat domain 19	TTC19	268	30417	3	115136,0082	106914,5782	0,92859376	0,85052783
523	F1MES6	UBX domain protein 6	UBXN6	441	49806	3	44198,86178	26189,01717	0,59252696	0,01965288

524	F1MEX9	Acyl-CoA synthetase long chain family member 3	ACSL3	720	80282	2	58495,89026	44260,16706	0,75663721	0,5263447
525	F1MEY2	Mitochondrial trans-2-enoyl-CoA reductase	MECR	388	41759	5	37639,65006	42249,39854	1,12247055	0,51394242
526	F1MFA3	Pitrilysin metalloproteinase 1	PITRM1	1032	116381	8	406436,9823	374303,8744	0,92093951	0,84229082
527	F1MFB3	Gem-associated protein 6	GEMIN6	588	68130	3	352048,1475	301475,5179	0,85634741	0,50227938
528	F1MFR7	Solute carrier family 9 member B1	SLC9B1	513	55287	3	11390,0755	7167,17388	0,62924727	0,10071534
529	F1MGC0	Succinate--CoA ligase [ADP-forming] subunit beta, mitochondrial (EC 6.	SUCLA2	463	50146	6	445100,9761	498196,8115	1,11928942	0,43914164
530	F1MGQ1	Deoxyribonuclease	DNASE1L3	305	34936	13	575078,0238	300623,0708	0,5227518	0,12390523
531	F1MGY9	Aspartylglucosaminidase	AGA	346	36965	20	788054,8447	1103107,243	1,39978486	0,04703222
532	F1MH21	Membrane metalloendopeptidase like 1	MMEL1	779	88991	2	168294,183	184269,9734	1,09492776	0,61975318
533	F1MHK9	ABC transporter domain-containing protein	ABCA14 LOC524:	1656	188419	10	297199,8661	316993,6742	1,066601	0,84932894
534	F1MI43	Sperm surface protein Sp17 (Sperm autoantigenic protein 17)	SPA17	147	16960	8	195788,7367	300995,4103	1,53734794	0,00583448
535	F1MIC3	[tau protein] kinase (EC 2.7.11.26)	GSK3B	492	55067	4	30337,86959	28276,92725	0,93206701	0,73968853
536	F1MIH2	Septin	SEPTIN7	417	48669	7	68177,33829	70149,01032	1,02891976	0,92819865
537	F1MIH9	Phospholipase B-like (EC 3.1.1.-)	PLBD2	589	65723	3	298331,4412	410231,7766	1,3750873	0,27207814
538	F1MIM1	Prostate and testis expressed 2		113	13259	1	202145,8003	149187,6325	0,73801995	0,33398848
539	F1MJ55	Casein kinase 2 alpha 2	CSNK2A2	350	41171	6	203617,3016	176981,526	0,86918707	0,35400431
540	F1MJ59	Nitrilase family member 2	NIT2	283	31159	1	46622,46257	51858,71425	1,11231178	0,39972499
541	F1MJQ7	Fibronectin type III domain containing 8	FNDC8	321	35343	41	908821,3137	987310,3741	1,08636358	0,76614056
542	F1MJS8	A-kinase anchoring protein 3	AKAP3	858	94740	154	19972416,45	18193486,01	0,91093064	0,40816762
543	F1MJY8	Immunity related GTPase cinema (Interferon-gamma-inducible GTPase	IRGC IFGGE	465	50697	9	2052168,887	1614208,819	0,78658673	0,30629786
544	F1MK41	Short chain dehydrogenase/reductase family 39U member 1	SDR39U1	294	31306	3	48767,03214	42313,68413	0,86766986	0,49832309
545	F1MK55	Dynein axonemal heavy chain 2	DNAH2	4424	508289	14	417141,038	414731,4605	0,99422359	0,94681603
546	F1MKF8	Sulfide quinone oxidoreductase	SQOR	450	49856	20	1034871,323	901961,0955	0,87156835	0,70133043
547	F1MLB8	ATP synthase subunit alpha	ATP5F1A	553	59690	92	206025,6278	194328,1989	0,94322343	0,96284643
548	F1MLM6	ITPRIP like 1	ITPRIPL1	547	62607	2	47742,15041	45342,1208	0,94972934	0,79037703
549	F1MLS7	Membrane spanning 4-domains A13	MS4A13	176	19737	1	42484,92215	51784,14152	1,21888282	0,31767331
550	F1MM32	Sulfhydryl oxidase (EC 1.8.3.2)	QSOX1	608	67021	7	159799,0101	184259,9882	1,1530734	0,27084842
551	F1MMV1	EF-hand domain-containing family member B (Cilia- and flagella-associa	EFHB CFAP21	877	97819	10	198092,5787	216704,7947	1,09395716	0,53822638
552	F1MMZ2	Ferritin	FTMT	242	27396	12	458402,3199	488192,5668	1,06498712	0,73935717
553	F1MN23	Dehydrogenase/reductase 7B	DHRS7B	325	35104	2	24813,65252	23234,2601	0,93634986	0,78876685
554	F1MNH2	Radial spoke head component 1	RSPH1	308	34964	7	143418,7587	131333,6433	0,91573546	0,35768935
555	F1MNL6	Nucleoporin 210 like	NUP210L	1884	209653	8	247873,7841	281212,9	1,13450037	0,52666532
556	F1MNT1	Nucleoporin 155	NUP155	1367	152487	7	411602,5579	481385,1332	1,16953873	0,74917286
557	F1MP86	Tetraspanin	TSPAN8	238	26208	8	366352,8089	475375,7989	1,29759016	0,0634285
558	F1MPF5	Sperm acrosome developmental regulator	SPACDR	200	22379	13	1486745,521	2336286,622	1,57140989	0,48986028
559	F1MPK4	3-hydroxyisobutyryl-CoA hydrolase (HIB-CoA hydrolase) (HIBYL-CoA-H)	HIBCH	386	43478	2	6505,329451	11950,53579	1,83703775	0,23234811
560	F1MQE8	Synaptojanin 2 binding protein	SYNJ2BP	145	15826	1	21664,14631	22561,45426	1,04141903	0,71276643
561	F1MQJ0	Angiotensin-converting enzyme (ACE) (EC 3.4.15.1) (Dipeptidyl carboxy	ACE DCP1	1306	150097	13	2404971,063	1256105,577	0,52229551	0,03501373
562	F1MQX0	Acetyl-coenzyme A synthetase (EC 6.2.1.1)	ACSS1	675	74294	22	45921,16773	56978,03203	1,24077925	0,24210205
563	F1MR35	Angiotensin-converting enzyme (EC 3.4.-.-)	ACE3	747	86747	12	1837362,008	2047014,566	1,1141052	0,44289698
564	F1MR53	Coiled-coil domain containing 27		305	36069	3	53417,14561	69769,40793	1,30612385	0,16457814
565	F1MRQ2	Chromosome 3 C1orf185 homolog	C3H1orf185	208	23881	2	13976,10944	41966,51191	3,00273206	0,00181165
566	F1MS25	NADH dehydrogenase [ubiquinone] iron-sulfur protein 3, mitochondria	NDUFS3	266	30240	17	1094060,53	1019920,317	0,9322339	0,5277261
567	F1MSR1	Protease, serine, 45	PRSS45	316	35251	1	35385,49675	29160,95026	0,82409328	0,96381146
568	F1MSV5	glycerol kinase (EC 2.7.1.30)	GK2	554	60868	41	2156441,412	2501608,98	1,1600635	0,22589713
569	F1MUB8	Spermatosis and centriole associated 1	SPATC1	415	44819	11	3940614,76	4300357,274	1,09129096	0,52685216
570	F1MUV1	fatty acid amide hydrolase (EC 3.5.1.99)	FAAH	579	63025	7	616819,481	614089,4445	0,99557401	0,99010805
571	F1MUZ9	60 kDa heat shock protein, mitochondrial (EC 5.6.1.7) (60 kDa chaperor	HSPD1	573	60978	43	2995441,024	3253195,303	1,08604886	0,48924269

572	F1MVL2	Acyl-CoA dehydrogenase short chain	ACADS	412	44444	12	872398,1551	892140,2256	1,02262966	0,81463258
573	F1MW14	RAN guanine nucleotide release factor	RANGRF	186	20714	5	81955,15043	68150,40755	0,83155735	0,9593879
574	F1MWD3	T-complex protein 1 subunit epsilon (CCT-epsilon)	CCT5	541	59601	6	184370,9573	159019,3952	0,86249699	0,34988384
575	F1MWF0	Huntingtin interacting protein 1	HIP1	1037	116494	4	101340,4659	81753,09428	0,80671717	0,39026805
576	F1MWN8	Cilia and flagella associated protein 58	CFAP58	867	103024	7	45335,98245	64618,14198	1,4253169	0,06673712
577	F1MWR3	Electron transfer flavoprotein subunit alpha (Alpha-ETF)	ETFA	333	34957	16	8372,492999	7805,227631	0,93224654	0,68365587
578	F1MWT0	alpha-mannosidase (EC 3.2.1.24)	MAN2C1	1040	115861	2	131569,3135	88975,29696	0,67626177	0,13416536
579	F1MWX4	Histone deacetylase 11	HDAC11	385	42832	2	133875,8435	120850,4367	0,90270532	0,40321538
580	F1MWY9	Enoyl-CoA delta isomerase 2	ECI2	393	42772	18	1038769,63	1137463,639	1,09501049	0,48334598
581	F1MX05	Saccharopine dehydrogenase (putative)	SCCPDH	429	47316	65	11072092,26	10345877,72	0,93441036	0,51504898
582	F1MX68	Carboxypeptidase (EC 3.4.16.-)	CPVL	593	66281	13	1639578,299	2113223,205	1,28888215	0,07212281
583	F1MX88	Solute carrier family 25 member 13	SLC25A13	642	70566	4	23758,18353	27255,49976	1,1472047	0,65417278
584	F1MXX0	MICOS complex subunit MIC60 (Mitofilin)	IMMT	749	83044	18	894713,7983	856099,3891	0,95684161	0,68258259
585	F1MXX5	Nardilysin convertase	NRDC	1192	136076	3	85053,82248	78300,16068	0,92059543	0,48042499
586	F1MXY8	Solute carrier family 25 member 1	SLC25A1	311	33780	7	790154,5416	759040,5067	0,96062285	0,76307569
587	F1MY12	NTR domain-containing protein	LOC112442231	243	28493	1	17984,7915	17984,96172	1,00000946	0,8954317
588	F1MY24	Chaperonin containing TCP1 subunit 8 like 2	CCT8L2	534	56607	6	315398,6774	288535,0107	0,91482632	0,47049428
589	F1MY32	Lymphocyte antigen 6 complex locus protein G5c	LY6G5C	141	16190	3	29576,41874	152697,7766	5,16282171	0,13265623
590	F1MY93	Leucine rich repeat containing 34	LRRC34	451	49618	1	47636,2539	58158,17272	1,22088048	0,17261042
591	F1MYA6	IZUMO family member 4	IZUMO4	213	24375	30	10272374,94	10567245,34	1,02870518	0,88745941
592	F1MYE8	Acyl-CoA dehydrogenase family member 9	ACAD9	594	65165	18	733978,5243	679448,2063	0,92570584	0,58122889
593	F1MYG0	Ornithine aminotransferase (EC 2.6.1.13)	OAT	439	48118	9	141775,8996	116860,7422	0,8242638	0,41584589
594	F1MYH5	A-kinase anchoring protein 4	AKAP4	848	94002	690	75067438	54862309,23	0,7308403	0,45854333
595	F1MZ38	Succinate--CoA ligase [ADP/GDP-forming] subunit alpha, mitochondrial	SUCLG1	346	36139	14	1088311,328	1346730,485	1,23744966	0,0748984
596	F1MZ78	Leucine rich repeat and coiled-coil centrosomal protein 1	LRRCC1	1027	118790	1	65289,07631	84924,32438	1,30074324	0,25273737
597	F1MZB5	Testis expressed 37	TEX37	180	20322	4	143807,5013	126578,3988	0,8801933	0,38403251
598	F1MZD5	Dehydrogenase/reductase (SDR family) member 4	DHRS4	279	29454	2	159095,5479	171635,5581	1,07882062	0,84045742
599	F1MZG1	Spermatosis associated 6	SPATA6	488	56206	4	106482,9881	124062,6847	1,16509395	0,24373364
600	F1MZJ1	Tektin bundle interacting protein 1	TEKTIP1	208	24526	9	713526,5526	630561,8771	0,88372588	0,39184687
601	F1MZR7	Spermatid maturation 1	SPEM1	313	35314	4	96276,40284	67498,04739	0,7010861	0,17559328
602	F1N058	Rhophilin associated tail protein 1	ROPN1	212	23978	64	17585345,08	16178962,46	0,9200253	0,58067916
603	F1N124	Leucine rich repeat containing 23	LRRC23	342	39611	3	99232,77903	124859,7123	1,25825069	0,19042286
604	F1N191	Interleukin 4 induced 1	IL4I1	578	64479	80	43958234,93	33756854,32	0,76793016	0,04344004
605	F1N1E1	Protein phosphatase 1 regulatory subunit 32	PPP1R32	428	47685	2	158810,837	170241,9235	1,07197926	0,96982092
606	F1N1M7	Carnitine palmitoyltransferase 2	CPT2	658	74455	5	46482,30694	53368,08029	1,14813751	0,38393856
607	F1N1W5	Nitrilase 1	NIT1	328	36222	1	11514,90015	14288,73132	1,2408906	0,33861587
608	F1N206	Dihydrolipoyl dehydrogenase (EC 1.8.1.4)	DLD	509	54187	33	2080726,879	2258669,016	1,08551922	0,50479934
609	F1N2F2	Phosphoglycerate mutase (EC 5.4.2.11) (EC 5.4.2.4)	PGAM2	253	28699	12	558329,311	475425,0346	0,85151366	0,45515881
610	F1N2S7	Testis specific serine kinase 6	TSSK6	273	30211	6	1296683,541	1034644,311	0,79791582	0,08388884
611	F1N2V1	Golgi associated RAB2 interactor family member 2	GARIN2	397	44273	7	675772,9818	687842,0876	1,0178597	0,98896829
612	F1N310	NADH-cytochrome b5 reductase (EC 1.6.2.2)	CYB5R2	273	30929	1	8397,015381	8238,948736	0,98117585	0,96780364
613	F1N338	Hydroxyacyl-CoA dehydrogenase	HADH	314	34391	4	902728,9826	743206,1995	0,82328829	0,64787464
614	F1N343	Coiled-coil domain containing 136	CCDC136	1201	138257	39	1396087,235	1449985,02	1,03860632	0,75053115
615	F1N369	Zona pellucida binding protein	ZPBP	350	39484	55	39486739,09	49308894,92	1,24874568	0,14619799
616	F1N3A8	Chromosome 27 C4orf47 homolog	C27H4orf47	309	34499	3	34577,77139	33026,4513	0,95513534	0,74579881
617	F1N3F1	LETM1 domain containing 1	LETMD1	360	41929	1	48781,14514	34954,28189	0,71655312	0,20237909
618	F1N3G6	Phospholipid-transporting ATPase (EC 7.6.2.1)	ATP11C	1119	128315	3	23739,79678	43280,98587	1,82314054	0,17634876
619	F1N3Q8	MIER family member 2	MIER2	561	61272	1	304161,1856	205852,159	0,67678642	0,72142331

620	F1N3R4	Mitochondrial proton/calcium exchanger protein (Electroneutral mitoc	LETM1	786	87860	6	109896,262	100486,3375	0,91437448	0,48235888
621	F1N3X1	IQ motif containing F5	LOC100125949	177	21626	2	128404,5641	132892,779	1,0349537	0,89622798
622	F1N402	Centrosomal protein of 44 kDa	CEP44	385	43562	1	16116,60369	20456,33874	1,26927107	0,51678416
623	F1N427	Septin	SEPTIN12	361	41134	8	126561,6621	128370,2224	1,01428995	0,99071883
624	F1N4A1	Armadillo repeat containing 12	ARMC12	338	38134	22	3769314,928	4473022,082	1,18669365	0,19452205
625	F1N4M3	Pyruvate dehydrogenase phosphatase regulatory subunit	PDPR	878	98841	5	190012,0823	157490,8044	0,82884626	0,28024755
626	F1N5C9	Radial spoke head 10 homolog B (Chlamydomonas)	RSPH10B	871	99777	2	63747,25697	53566,23219	0,84029078	0,71721201
627	F1N5R7	Dynein axonemal heavy chain 7	DNAH7	4024	461307	33	441213,6594	400333,5401	0,90734621	0,37605333
628	F1N690	Acetyltransferase component of pyruvate dehydrogenase complex (EC	DLAT	647	69067	18	6315254,232	6186836,889	0,97966553	0,78684165
629	F1N6A0	Isochorismatase domain containing 2	ISOC2	204	22388	11	419126,1468	373505,99	0,89115411	0,45917126
630	F1N6I4	NADP-dependent oxidoreductase domain-containing protein	LOC788425	334	36823	1	96022,96282	101214,01	1,05406048	0,52010138
631	F1N6K8	Uncharacterized protein	LOC789612	275	29927	18	2280723,999	2044302,348	0,89633921	0,58552587
632	F1N6M5	Coiled-coil domain containing 183	CCDC183	534	62388	2	21747,92919	25209,16397	1,15915238	0,64243527
633	F1N7K1	Rhophilin associated tail protein 1 like	ROPN1L	218	24422	10	1367231,516	1519041,112	1,1110343	0,36467843
634	F1N7P3	EF-hand domain-containing protein		495	55574	3	340532,6426	212745,0556	0,62474203	0,15955434
635	F1N7X7	Golgi associated RAB2B interactor family member 3	GARIN3	587	62663	35	2535832,944	2600168,821	1,02537071	0,7865151
636	F2Z4D5	RAB11A, member RAS onco family	RAB11A	216	24394	1	31740,9423	35392,22832	1,11503395	0,7003536
637	F6QGE6	Acyl-CoA thioesterase 13	ACOT13	236	25627	3	46917,66636	46484,07746	0,99075852	0,79366612
638	F6QJG7	Zona pellucida binding protein 2	ZPBP2	326	37409	13	353129,8483	350139,7237	0,99153251	0,86663171
639	F6QQ46	Chromosome 10 C15orf48 homolog	C10H15orf48	110	12678	11	1952703,689	2251419,228	1,15297535	0,28103364
640	F6R6Q1	FAD synthase (EC 2.7.7.2) (FAD pyrophosphorylase) (FMN adenylyltrans	FLAD1	490	54273	2	59140,84766	46323,73757	0,78327821	0,18130959
641	F6RFP6	Basigin	BSG	320	34897	5	381101,7164	477133,2841	1,25198409	0,2809914
642	F6RJC2	Cilia- and flagella-associated protein 161	CFAP161	321	36470	7	412783,2998	452250,3437	1,09561202	0,50600253
643	F6RP72	Tubulin alpha chain	TUBA3E	458	50886	138	63157529,26	67435760,75	1,06773906	0,63574994
644	G1K1H0	ADAM metallopeptidase domain 2	ADAM2	745	83127	37	567894,1581	722024,124	1,27140615	0,2477989
645	G3MWG4	isoleucine--tRNA ligase (EC 6.1.1.5) (Isoleucyl-tRNA synthetase)	IARS2	1012	113608	10	199649,1835	170129,6557	0,85214301	0,33621176
646	G3MY76	Cylicin 1	CYLC1	655	73443	33	1363465,03	1584498,676	1,16211171	0,32366414
647	G3MY97	Golgi associated RAB2 interactor protein-like Rab2B-binding domain-co		794	87181	2	80616,06115	79219,52227	0,98267667	0,72144265
648	G3MZ01	Profilin		153	17049	1	77587,6946	116959,4866	1,50744892	0,10617161
649	G3MZM7	Uncharacterized protein		520	55964	9	114523,3651	204635,046	1,78684102	0,01210013
650	G3MZM8	vitamin-K-epoxide reductase (warfarin-sensitive) (EC 1.17.4.4)	VKORC1L1	176	19832	2	390918,5307	304731,8778	0,77952784	0,07694808
651	G3MZS1	Small integral membrane protein 13	SMIM13 LOC112	87	9939	1	17459,97562	18539,10647	1,06180598	0,70529037
652	G3MZX2	Proline rich 30	PRR30	398	42785	9	32424,65517	41746,77752	1,28750105	0,20129387
653	G3MZZ7	Family with sequence similarity 170 member B	FAM170B	289	32536	1	2809,838386	5431,257285	1,93294294	0,04261993
654	G3N0F7	Testis expressed 50	TEX50	179	20784	5	79435,00084	109639,3074	1,38023927	0,13195982
655	G3N0V2	Keratin, type II cytoskeletal 1 (Cytokeratin-1) (Keratin-1) (Type-II keratir	KRT1	606	63165	18	941304,4037	953502,8652	1,0129591	0,9833496
656	G3N136	Testis specific serine kinase 2	TSSK2	358	40763	13	281817,1872	427829,0931	1,51810859	0,03302128
657	G3N284	Actin related protein T1	ACTRT1	377	42131	9	89179,53603	119401,4353	1,33888828	0,09026196
658	G3N2D2	Testis specific serine kinase 1B	TSSK1B	367	41541	5	21236,25839	23356,43024	1,09983735	0,38081325
659	G3N2I2	Chromosome 11 C2orf81 homolog	C11H2orf81	606	65873	1	34025,89231	40855,26122	1,20071094	0,71519883
660	G3N3B6	Coiled-coil domain containing 188	CCDC188	497	53253	5	12855,87198	20300,15164	1,57905677	0,15872187
661	G3X6A7	Outer dense fiber of sperm tails 3	ODF3	254	27643	11	1954468,149	2052613,552	1,05021591	0,85235161
662	G3X6E2	Family with sequence similarity 166 member A	FAM166A	320	36204	32	2977767,015	3134063,122	1,05248769	0,60999753
663	G3X7X2	Solute carrier family 25 member 32	SLC25A32	317	35618	3	285432,7434	308612,935	1,0812107	0,80589574
664	G3X800	Disintegrin and metalloproteinase domain-containing protein 20-like		724	82069	18	553765,1948	685259,3121	1,23745464	0,44941896
665	G3X807	Histone H4	H4C9	98	10949	6	7348823,197	2930322,01	0,39874711	0,01103348
666	G3X8G1	ATP6V1F neighbor	ATP6V1FNB	176	20248	3	65292,84643	80119,44387	1,22707844	0,30483183
667	G5E519	Coiled-coil domain-containing protein 39	CCDC39	941	109998	2	19211,95569	15389,4312	0,80103408	0,80636175

668	G5E521	NAD-dependent protein deacetylase (EC 2.3.1.286)	SIRT3	333	36992	7	390709,1227	370426,0888	0,94808661	0,58597938
669	G5E531	T-complex protein 1 subunit alpha (CCT-alpha)	TCP1	556	60222	13	575426,3436	472141,4455	0,82050718	0,09645505
670	G5E5C6	Acrosin (EC 3.4.21.10)	ACR	414	45494	64	70208831,17	81104700,42	1,15519229	0,25864038
671	G5E6S5	Coiled-coil domain containing 159	CCDC159	337	38411	5	142788,9657	182974,7128	1,28143454	0,13502928
672	G8CY12	Beta-defensin		89	9374	5	60801,24334	110739,9307	1,82134319	0,00303262
673	I6NUJ4	Choline transporter-like protein	SLC44A5	717	81886	4	111362,3959	84785,77018	0,76135009	0,14819835
674	M0QVZ6	Keratin 5	KRT5	594	62624	10	1124151,126	1393299,077	1,23942328	0,70557748
675	M4MFL5	Trypsin-like serine protease-like protein		301	32668	1	31168,52761	23640,28229	0,75846644	0,43221724
676	M5FHL5	NADH dehydrogenase [ubiquinone] 1 beta subcomplex subunit 10 (Cor	NDUFB10 BOS_2	176	20965	4	179313,6165	175377,2854	0,97804778	0,88773849
677	M5FJY9	Phosphoglycolate phosphatase (EC 3.1.3.18)	PGP BOS_22504	321	34320	3	51506,57268	110544,9271	2,14622953	0,03430759
678	M5FK65	Protease, serine, 21-like	PRSS21 BOS_225	485	53800	1	16649,20544	17955,30087	1,07844791	0,51049215
679	M5FK83	CG2446-like	LOC516108 BOS_	232	25065	2	142625,5514	185880,5305	1,30327651	0,13058666
680	M5FKE0	Tektin	TEKT4 BOS_2246	447	51945	41	48363,17628	46063,71395	0,95245427	0,64099626
681	M5FMT6	Hydroxyacylglutathione hydrolase, mitochondrial (EC 3.1.2.6)	HAGH BOS_2245	308	34089	5	14785,89717	16954,51697	1,14666812	0,67561489
682	N0E4A8	Casein kinase II subunit beta (Phosvitin)	Csnk2b-Ly6g5b	233	26239	3	45163,40091	75536,08644	1,67250661	0,0932016
683	O77779	Fertilin alpha	ADAM 1	812	89905	20	608296,1148	1028431,16	1,69067521	0,00546362
684	P04394	NADH dehydrogenase [ubiquinone] flavoprotein 2, mitochondrial (EC 7	NDUFV2	249	27308	6	475153,7933	642133,7568	1,35142298	0,62844658
685	P16368	Metalloproteinase inhibitor 2 (Collagenase inhibitor) (Tissue inhibitor o	TIMP2	220	24355	3	281178,4456	211439,447	0,75197601	0,4340231
686	P29392	Spermadhesin-1 (Acidic seminal fluid protein) (ASFP)	SPADH1	134	15036	4	134607,3108	148618,1421	1,1040867	0,78841529
687	Q00361	ATP synthase subunit e, mitochondrial (ATPase subunit e) (ATP synthas	ATP5ME ATP5I	71	8321	4	523589,2362	504747,2674	0,96401383	0,92061751
688	Q02827	NADH dehydrogenase [ubiquinone] 1 subunit C2 (Complex I-B14.5b) (C	NDUFC2	120	14096	2	789969,0503	854993,1332	1,08231219	0,53481741
689	Q05AT5	HIG1 domain family, member 2A (HIG1 hypoxia inducible domain famil	HIGD2A	106	11575	1	21857,57129	26248,61161	1,20089333	0,54978989
690	Q08DM3	Malic enzyme	ME2	584	65413	2	15784,26725	13306,28081	0,84300909	0,46370341
691	Q08DN5	Carnitine O-acetyltransferase (Carnitine acetyltransferase)	CRAT MGC14278	626	71017	14	544201,5513	534426,833	0,98203842	0,88407373
692	Q0II40	Dual specificity phosphatase 21 (Similar to Dual specificity protein phos	DUSP21 LOC538	196	22052	6	160492,5368	179320,266	1,11731218	0,48066292
693	Q0IIB9	Uncharacterized protein LOC777692	LOC777692	106	12606	1	30726,58566	14145,02657	0,46035139	0,13119969
694	Q0V8B1	Protein Flattop (Cilia- and flagella-associated protein 126)	CFAP126 C1orf1	216	23381	1	71879,92957	94974,01473	1,32128698	0,24699116
695	Q0VCS8	glutathione transferase (EC 2.5.1.18)	GSTT3	239	27277	2	143534,8791	187513,3189	1,30639549	0,30828977
696	Q0VCU3	Cathepsin F	CTSF	460	50893	17	10298980,78	13611835,81	1,32166824	0,04631286
697	Q148D3	Fumarate hydratase, mitochondrial (EC 4.2.1.2)	FH	510	54691	30	2680883,355	2740018,872	1,02205822	0,85131699
698	Q148I4	Acyl-CoA thioesterase 7	ACOT7	338	37368	2	98948,90804	79907,40881	0,80756231	0,18587974
699	Q148J8	Isocitrate dehydrogenase [NAD] subunit, mitochondrial	IDH3A	366	39698	10	923868,973	968777,543	1,04860924	0,66023484
700	Q148N0	2-oxoglutarate dehydrogenase complex component E1 (E1o) (OGDC-E1	OGDH	1023	115808	39	1257566,771	1573398,493	1,25114509	0,1249621
701	Q17QF5	Protein kinase cAMP-dependent type I regulatory subunit beta (Protein	PRKAR1B	381	43032	4	81261,68114	87504,56861	1,07682449	0,7524437
702	Q17QY3	Nipsnap homolog 3A (C. elegans)	NIPSNAP3A	247	28540	1	7347,51802	8540,374822	1,16234826	0,99409224
703	Q1JPA2	Elongation factor 1-gamma (eEF-1B gamma)	EEF1G	439	50231	4	205918,1524	110122,2921	0,53478671	0,06114674
704	Q1JPF2	NADH dehydrogenase (Ubiquinone) 1 alpha subcomplex, 9, 39kDa	NDUFA9	369	41660	12	1150940,647	1102599,409	0,9579985	0,66870292
705	Q1LZ81	PDCD6 protein (Programmed cell death 6)	PDCD6	189	21737	3	48434,45417	42258,82914	0,87249521	0,45476915
706	Q1RMN5	Spermatogenesis associated 3 (Spermatosis associated 3)	SPATA3	230	24393	2	264189,3627	358266,4583	1,35609721	0,1247135
707	Q1RMP3	CutA divalent cation tolerance homolog (E. coli)	CUTA	153	16347	4	118319,297	93112,75178	0,78696167	0,18677566
708	Q24JZ7	Succinyl-CoA:3-ketoacid-coenzyme A transferase (EC 2.8.3.5)	OXCT1	520	56453	15	575696,2684	629189,6504	1,09291945	0,45426668
709	Q28092	Cylicin-2 (Cylicin II) (Multiple-band polypeptide II)	CYLC2 CYL2	488	53562	26	204498,6947	220442,2374	1,07796403	0,59938634
710	Q29RT2	Hypothetical LOC540061	LOC540061	242	27071	2	14161,11854	26020,43358	1,8374561	0,28982512
711	Q29RT3	MS4A13 protein	NYD-SP21	861	97385	3	41874,28747	46595,38243	1,11274448	0,51791004
712	Q29RU5	Rhabdoid tumor deletion region gene 1	RTDR1	379	41493	6	157481,0517	132237,3725	0,83970339	0,25333873
713	Q2HJI1	Succinate dehydrogenase [ubiquinone] flavoprotein subunit, mitochon	SDHA	678	74407	35	2511838,674	3254633,401	1,29571753	0,15264883
714	Q2KIC7	Serine/threonine-protein phosphatase (EC 3.1.3.16)	PPP6C	305	35144	5	54968,34602	32513,04298	0,59148665	0,09418213
715	Q2KII5	Histone H2B	H2BC5 HIST1H2E	126	13936	5	5685283,227	1526045,591	0,26842033	0,02965578

716	Q2KIK5	LOC767871 protein	LOC767871	392	43229	7	132644,1284	163949,8903	1,23601318	0,2612949
717	Q2KIV7	inorganic diphosphatase (EC 3.6.1.1)	PPA2	327	36963	3	20772,37272	14387,99795	0,69265067	0,27870464
718	Q2KIV8	Glutathione S-transferase (EC 2.5.1.18)	GSTM3	225	26850	105	60121864,61	56657677,62	0,94238058	0,54945774
719	Q2KIZ0	TSBP protein	TSBP	573	62599	2	26952,59351	24843,73982	0,92175693	0,75718001
720	Q2KJH9	4-trimethylaminobutyraldehyde dehydrogenase (TMABA-DH) (TMABAI	ALDH9A1	494	53977	5	313819,2532	284700,1649	0,90721064	0,66759205
721	Q2NKS5	Chromosome 22 C3orf84 homolog (Uncharacterized protein MGC1370	C22H3orf84 MG(	204	23462	3	155221,6695	174369,4407	1,12335759	0,44788848
722	Q2NKS8	peptidylprolyl isomerase (EC 5.2.1.8)	LOC526524	108	11945	3	95169,28294	100347,4622	1,0544102	0,6489797
723	Q2NKT6	Uncharacterized protein MGC137036	MGC137036	112	12953	1	53651,82309	42991,96053	0,80131407	0,7343498
724	Q2NKZ3	ADAM metallopeptidase domain 32	ADAM32	747	82546	22	8494384,2	10420177,5	1,2267137	0,22307392
725	Q2NKZ8	glycerol kinase (EC 2.7.1.30)	LOC538702	553	60602	43	9951232,871	9506304,284	0,9552891	0,69317218
726	Q2NL07	Farnesyl pyrophosphate synthase ((2E,6E)-farnesyl diphosphate syntha	FDPS	421	48333	11	45663,48825	31148,3042	0,68212713	0,15968556
727	Q2T9Y3	Pyruvate dehydrogenase E1 component subunit alpha (EC 1.2.4.1)	PDHA2	391	43292	22	950440,8629	1302376,796	1,37028704	0,06593439
728	Q2TA15	Chromosome 15 open reading frame 23 ortholog (Kinetochore localize	KNSTRN C10H15	234	25676	1	37563,49824	43325,67275	1,15339824	0,88075926
729	Q2TA22	Long-chain-fatty-acid--CoA ligase (EC 6.2.1.15) (EC 6.2.1.3) (Acyl-CoA sy	ACSL6	697	78043	15	696620,7581	531733,291	0,76330383	0,05648963
730	Q2TA28	Signal-regulatory protein delta	SIRPD	286	31467	7	179703,4234	245900,5494	1,36836875	0,09623644
731	Q2TA33	Membrane cofactor protein	LOC616002	397	45796	8	307977,6303	373317,0349	1,21215633	0,37699091
732	Q2TBH3	Actin related protein M1 (Actin related protein T3)	ACTRT3 ARPM1	368	40943	26	4110372,157	4258714,798	1,03608983	0,79290588
733	Q2TBP9	Coilin	COIL	571	63122	3	73816,91455	79996,15096	1,0837103	0,54067459
734	Q2TBR1	5'-nucleotidase, cytosolic IB	NT5C1B	564	64011	90	4087811,175	4397359,974	1,07572483	0,71347186
735	Q2TBS5	Serine peptidase inhibitor, Kazal type 2 (Acrosin-trypsin inhibitor) (Serir	SPINK2B SPINK2	84	9391	3	357170,716	715065,8866	2,00202831	0,09522712
736	Q2TBW4	Polycystic kidney disease 2-like 2 (Polycystin 2 like 2, transient receptor	PKD2L2	600	71610	1	45709,15155	48569,25503	1,06257179	0,99184768
737	Q2YDK3	Hyaluronidase (EC 3.2.1.35)	SPAM1	553	62299	49	13558901,45	15101405,78	1,11376322	0,5323296
738	Q2YDK7	Thioredoxin domain containing 3 (Spermatozoa)	TXNDC3	410	46919	7	231403,4081	250426,4706	1,08220736	0,42017896
739	Q2YDL8	Uncharacterized protein LOC780846	LOC780846	243	27324	7	149434,7079	126327,6539	0,84537023	0,98140476
740	Q2YDN8	Inactive serine/threonine-protein kinase VRK3 (Serine/threonine-prote	VRK3	451	50549	15	2328092,259	2371979,586	1,0188512	0,83441031
741	Q2YDP2	Sad1 and UNC84 domain containing 5 (Sperm associated antigen 4-like	SUN5 SPAG4L	372	42629	8	11659,00038	12250,81276	1,05076013	0,71060116
742	Q32KP0	Spermatid-specific manchette-related protein 1 (Ciliated bronchial epit	SMRP1 CBE1	304	35066	37	3130000,783	2077357,668	0,66369238	0,34312026
743	Q32KP4	Abhydrolase domain containing 16B (Chromosome 20 open reading fra	ABHD16B C13H2	470	52624	4	444116,2039	335432,2034	0,75528026	0,04443936
744	Q32KP8	Serine peptidase inhibitor-like, with Kunitz and WAP domains 1 (Eppin)	SPINLW1	134	15093	2	68170,43159	32619,37481	0,47849741	0,09912688
745	Q32KR0	TBC1 domain family, member 21	TBC1D21	299	34883	1	32670,28211	15293,96486	0,46813079	0,00675641
746	Q32KR2	Acrosomal vesicle protein 1	ACRV1	273	28953	26	348619,3419	339539,2866	0,97395424	0,81717866
747	Q32KR7	Hypothetical LOC539526	LOC539526	477	54623	20	710987,188	840834,881	1,18263014	0,35639334
748	Q32KS3	F-actin-capping protein subunit alpha	CAPZA3	299	35066	67	17588876,91	19649060,44	1,11712991	0,52253531
749	Q32L04	Late cornified envelope-like proline-rich protein 1	LELP1	110	11767	11	76879,04789	150618,4139	1,95916076	0,00263338
750	Q32L54	Sperm associated antigen 6	SPAG6	509	55443	24	1929561,268	1707127,721	0,88472325	0,25677625
751	Q32L61	Calcium binding tyrosine phosphorylation regulated (Calcium binding ty	CABYR	454	48564	29	1262442,185	1154331,145	0,91436357	0,63491607
752	Q32L64	Similar to DnaJ (Hsp40) homolog, subfamily B, member 3	LOC528549	244	26915	9	203683,888	170752,4529	0,83832086	0,36605105
753	Q32L74	Aquaporin 7	AQP7	330	35948	7	689542,9133	708731,4075	1,02782785	0,79477052
754	Q32L87	DDB1 and CUL4 associated factor 7 (WD repeat domain 68)	DCAF7 WDR68	342	38926	1	25954,42967	32100,85965	1,23681622	0,32987008
755	Q32LB7	V-type proton ATPase subunit E 2 (V-ATPase subunit E 2) (Vacuolar pro	ATP6V1E2	226	26171	3	192019,3583	144080,8506	0,75034544	0,14744666
756	Q32LI7	Testis expressed 33 (Uncharacterized protein MGC133880)	TEX33 MGC1338	272	30275	1	6363,414511	11139,19649	1,75050619	0,29146961
757	Q32LN4	Cilia- and flagella-associated protein 45	CFAP45 CCDC19	549	65666	6	176545,2512	236435,4402	1,33923421	0,06950622
758	Q32LN6	Protein SPATA31F3 (Protein FAM205C)	SPATA31F3 FAM	408	45146	27	4500310,283	4577455,605	1,01714222	0,94182811
759	Q32LP8	Cysteine-rich secretory protein 2	CRISP2	244	27143	6	74273,0965	111885,0465	1,50640073	0,07534665
760	Q32P61	Histone H2A	H2AL1Q LOC523(	117	13150	11	216335,06	431177,6457	1,9931011	0,00319239
761	Q32PA1	CD59 molecule (CD59 molecule, complement regulatory protein)	CD59	121	13663	2	52188,95779	47967,0979	0,91910435	0,97135024
762	Q32S21	FabG-like protein (Hydroxysteroid (17-beta) dehydrogenase 8) (Hydrox	HSD17B8 FABGL	259	26468	3	154862,0144	142035,4237	0,91717407	0,47228279
763	Q3MHX0	sulfite oxidase (EC 1.8.3.1)	SUOX	547	60501	8	570363,6397	634662,7813	1,11273359	0,97929205

764	Q3SWX2	Acyl-coenzyme A thioesterase 9, mitochondrial (Acyl-CoA thioesterase	ACOT9	437	49853	15	317211,5745	294451,77	0,9282504	0,53274181
765	Q3SZ00	enoyl-CoA hydratase (EC 4.2.1.17)	HADHA	763	83249	24	4115373,187	3698308,522	0,8986569	0,36371648
766	Q3SZ15	Eukaryotic translation elongation factor 1 epsilon 1	EEF1E1	174	19757	2	88089,51466	62396,97043	0,70833595	0,17600935
767	Q3SZA4	Solute carrier family 25 (Carnitine/acylcarnitine translocase), member 2	SLC25A20	301	32928	2	173929,5444	156737,7513	0,90115657	0,91470155
768	Q3SZK3	Growth hormone inducible transmembrane protein	GHITM	345	37029	4	330734,7818	352814,1864	1,06675864	0,66311328
769	Q3SZT4	Testis-expressed protein 29	TEX29	147	16331	9	114473,9312	148708,1289	1,29905671	0,07794344
770	Q3SZW9	DnaJ (Hsp40) related, subfamily B, member 13 (DnaJ heat shock protei	DNAJB13	316	36077	13	896627,7212	917948,3346	1,02377867	0,77521249
771	Q3SZX1	Cytochrome c oxidase subunit 7B2, mitochondrial (MGC127695 protei	MGC127695 CO)	78	8983	1	27702,16878	44661,28964	1,6121947	0,43079536
772	Q3T010	Phosphatidylethanolamine-binding protein 4	PEBP4	223	25146	15	1530282,599	1816646,446	1,18713135	0,49187462
773	Q3T034	Serine rich single-pass membrane protein 1 (Uncharacterized protein M	SSMEM1 MGC12	241	27622	5	8710,770403	17351,4713	1,99195599	0,00253596
774	Q3T056	L-lactate dehydrogenase A-like 6B (EC 1.1.1.27)	LDHAL6B	381	41592	27	9415377,732	11412686,67	1,21213264	0,16943379
775	Q3T0U3	Es1 protein (Glutamine amidotransferase class 1 domain containing 3)	ES1 GATD3	274	28699	6	348889,3126	401390,3401	1,15048047	0,51548864
776	Q3T0Z0	WAP four-disulfide core domain 2	WFDC2	123	12729	2	44021,72213	29810,61187	0,67717959	0,19816685
777	Q3T172	ECH1 protein	ECH1	347	37572	3	88985,37768	102231,0992	1,14885279	0,51347879
778	Q3ZBG1	Ras-related protein Rab-14	RAB14	215	23897	3	18450,69594	29648,31269	1,606894	0,29596947
779	Q3ZBY4	Fructose-bisphosphate aldolase (EC 4.1.2.13)	ALDOC	364	39382	21	32561,89181	83455,88333	2,56299246	0,01910574
780	Q4R0H2	Spermadhesin 2	spadh2 SPADH2	134	15221	4	263806,7552	170953,9033	0,64802701	0,07927892
781	Q58D70	Serine/threonine-protein phosphatase (EC 3.1.3.16)	PPP2CA	309	35580	1	10430,81783	8750,728939	0,83893028	0,25324408
782	Q58DM0	Isocitrate dehydrogenase [NAD] subunit, mitochondrial	IDH3G	392	42873	5	656874,9655	476879,7529	0,72598254	0,02445196
783	Q5E974	Deoxyguanosine kinase isoform a	DGUOK	277	32043	3	52574,66881	40091,54107	0,76256383	0,65106991
784	Q5RZ69	Sperm mitochondria-associated cysteine-rich protein	SMCP	103	10977	4	30573,54222	51955,97715	1,69937709	0,12242877
785	Q7JAT1	ATP synthase subunit a	ATP6 atp6 ATPas	226	24788	1	8886,580513	6048,965241	0,68068536	0,0852094
786	Q7JAT2	ATP synthase protein 8	ATP8 atp8 ATPas	66	7937	3	92826,16146	111763,5757	1,20400945	0,17444057
787	Q862J3	V-type proton ATPase subunit D (V-type proton ATPase subunit d) (Vac		165	18391	1	11293,54956	14806,7821	1,31108311	0,18296844
788	Q862K7	Dynein light chain		78	9252	24	1756246,665	1291651,114	0,73546111	0,23371645
789	Q8HZY1	Serine protease inhibitor clade E member 2 (Serine protease inhibitor-E	SERPINE2	397	43877	5	168736,3716	127812,5076	0,75746863	0,22541858
790	Q8M444	Cytochrome c oxidase subunit 2	COX2	227	26020	24	1865299,623	2160408,286	1,15820979	0,28296273
791	Q9N273	Kappa-casein	CSN3	160	17879	1	16910,25899	15167,1773	0,89692164	0,82099294
792	Q9N2J2	Phospholipid hydroperoxide glutathione peroxidase (PHGPx) (EC 1.11.1	GPX4	197	22240	82	133405312,2	142378444,1	1,06726218	0,56601998
793	Q9XSJ4	Alpha-enolase (EC 4.2.1.11) (2-phospho-D-glycerate hydro-lyase) (Enola	ENO1	434	47326	53	9040832,556	11146672,59	1,23292545	0,22134477
794	U3GR08	NADH-ubiquinone oxidoreductase chain 4 (EC 7.1.1.2)	ND4	459	52113	2	9227,945155	6747,490729	0,73120187	0,63438698
795	U3GU87	Cytochrome c oxidase subunit 1 (EC 7.1.1.9)	COX1	514	57062	1	65098,04796	57047,49287	0,87633185	0,25506102
796	U3GXK6	Cytochrome c oxidase subunit 3	COX3	260	29876	2	45925,3674	50647,20105	1,10281537	0,47376425
797	U3GXM3	NADH-ubiquinone oxidoreductase chain 1 (EC 7.1.1.2)	ND1	318	35710	1	15529,14995	23779,26588	1,53126642	0,1442694
798	V6F7X8	Oligoribonuclease, mitochondrial	REXO2 BOS_143	237	26885	1	23090,70873	12611,60813	0,54617674	0,03329023
799	V6F959	ATP synthase subunit	ATP5L BOS_1436	104	11621	6	1710541,143	1786144,832	1,0441987	0,85817615
800	V9VJQ3	Sperm flagella 2 protein (Sperm flagellar 2)	SPEF2	1713	196868	1	30328,06173	25032,72117	0,82539799	0,26565193
801	W0UV03	Ribonuclease A C1	RAC1	150	16377	13	79150,668	77857,62984	0,98366359	0,80690483
802	W0UVF3	Ribonuclease A M1	RAM1	146	17714	4	349020,1182	227205,881	0,65098219	0,25251651
